# Supplementary material for: Stretchable phosphorescent polymers by multiphase engineering
Source: Nat Commun. 2024 May 15;15:4113. doi: 10.1038/s41467-024-47673-y (PMC11096371; doi:10.1038/s41467-024-47673-y)
Supplement: Supplementary file 1 — Supplementary Information [file 41467_2024_47673_MOESM1_ESM.pdf]

Supplementary Information

Stretchable phosphorescent polymers by multiphase engineering

Nan Gan[1], Xin Zou[1], Zhao Qian[2], Anqi Lv[3], Lan Wang[3], Huili Ma[3], Hu-Jun Qian[2], Long Gu[1,4*], Zhongfu An[3*], Wei Huang[1,3*]

[1]*Frontiers Science Center for Flexible Electronics (FSCFE), MIIT Key Laboratory of Flexible Electronics (KLoFE), Northwestern Polytechnical University, Xi'an 710072, P.R. China.*
[2]*State Key Laboratory of Supramolecular Structure and Materials, Institute of Theoretical Chemistry, College of Chemistry, Jilin University, Changchun 130012, P.R. China.*
[3]*Key Laboratory of Flexible Electronics (KLoFE) & Institute of Advanced Materials (IAM), Nanjing Tech University (NanjingTech), 30 South Puzhu Road, Nanjing, 211816, P.R. China.*
[4]*Research and Development Institute of Northwestern Polytechnical University in Shenzhen, Shenzhen 518057, P. R. China.*
**Correspondence to: iamlgu@nwpu.edu.cn (L.G.); iamzfan@njtech.edu.cn (Z.A.); vc@nwpu.edu.cn (W.H.)*

Contents

I. Supplementary Methods

II. Supplementary Discussion

2.1 Additional photophysical and mechanical characterizations

2.2 Mechanism investigation of stretchable phosphorescent polymers

2.3 Theoretical calculations

2.4 Additional experiments for the universality of multiphase engineering

2.5 Applications

III. Supplementary References

I. Supplementary Methods

Measurements

Nuclear magnetic resonance (^1H and ^{13}C NMR) spectra were recorded on a Bruker Advance Neo 500 MHz spectrometer using $CDCl_3$ or DMSO-d_6 as the solvent. The purity and molecular weight of ATRP initiators were confirmed by high-performance liquid chromatography-mass spectrometry (Thermofisher, HPLC-MS). The flow rate was 1.0 mL min^{-1}, the injection volume was 10 μL and each sample was run for 25 min. The absorption wavelength was set at 254 nm. 75% of acetonitrile and 25% of water were used as the mobile phase. Elemental analyses were performed on a Vario EL Cube. Gel permeation chromatography (GPC) measurements were performed on an Agilent 1260 HPLC system equipped with a G7110B pump and a G7162A refractive index detector, tetrahydrofuran was used as the eluent at 0.5 mL min^{-1} flow rate and PMMA as the standard. Fourier transform infrared spectroscopy (FTIR) spectra were recorded on a Nicolet 560 spectrometer. Steady-state fluorescence and phosphorescence spectra and lifetimes were measured using a fluorescence spectrophotometer (Edinburgh FLS1000) equipped with a xenon arc lamp (Xe900), a nanosecond hydrogen flash-lamp (nF920), or a microsecond flash-lamp (μF900), and all the phosphorescence spectra were recorded with a delay time of 8 ms. Photoluminescence efficiencies were collected on a Hamamatsu absolute PL quantum yield spectrometer C11347. The ultraviolet-visible (UV-vis) optical absorption and transmission spectra were measured by a spectrophotometer (U-3900H, Hitachi, Japan). The mechanical properties of polymers were tested by a universal testing machine (Instron 3344) with rectangular specimens of polymer films with dimensions of 30 mm × 10 mm × 0.5 mm at a tensile rate of 5 mm min^{-1} at room temperature. Transmission electron microscopy (TEM) was carried out using a JEOL 2000 EX electron microscope operated at 200 kV. Wide-angle X-ray scattering (WAXS) patterns were achieved from a Xenocs Nanoinxider with Cu-Kα micro source (40 mm) at 30 W. Scattering data were obtained from a small-angle X-ray scattering (SAXS) apparatus Xeuss 2.0. Powder X-ray diffraction patterns were recorded on a Bruker D8 Advance X-ray diffractometer with Cu-Kα radiation. The thermal analysis data of TGA and DSC was tested by DSC 214 and TG 209 F3 instruments under a nitrogen atmosphere. Luminescent photographs and movies were taken by a Canon EOS 850D camera.

General procedure for the synthesis of initiators and block copolymers

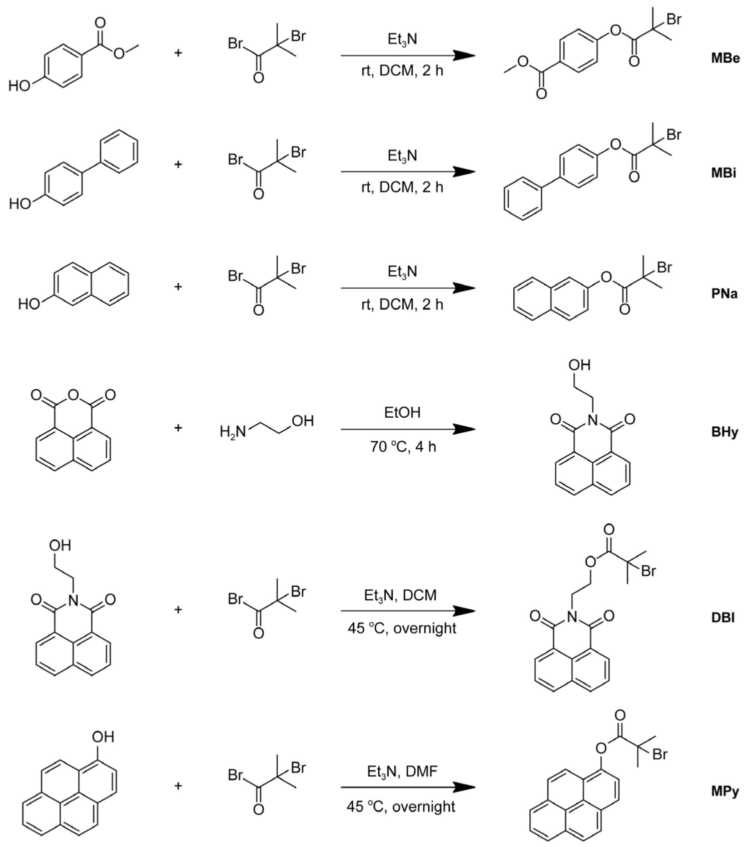

Supplementary Figure 1. Synthetic routes of initiators MBe, MBi, PNa, DBI, and MPy.

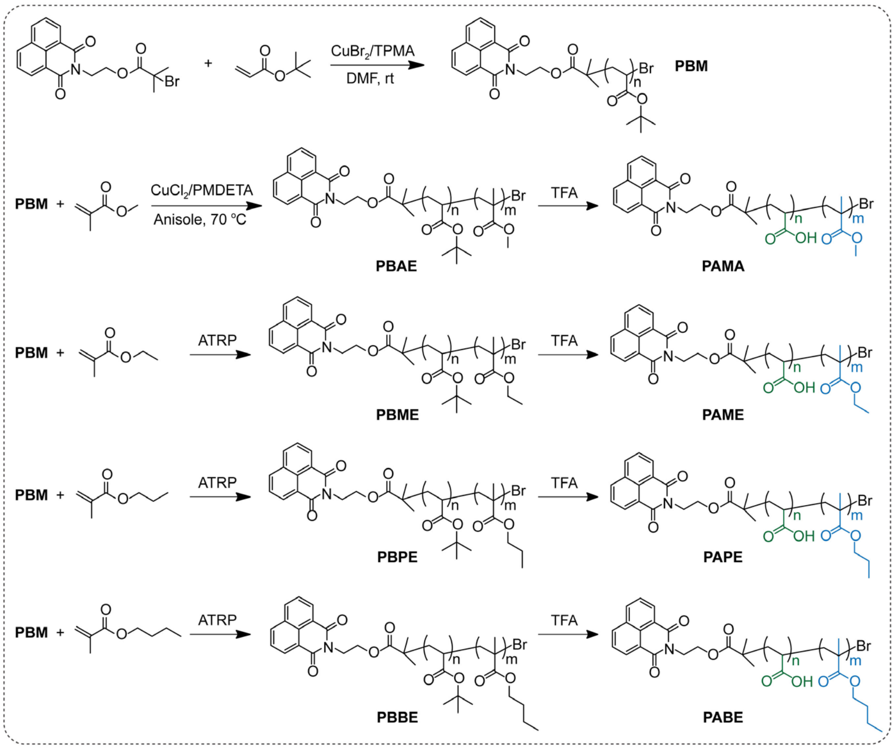

Supplementary Figure 2. Synthetic routes of ATRP macroinitiator PBM, copolymers PBAE, PBME, PBPE, and PBBE, and amphiphilic block copolymers PAMA, PAME, PAPE, and PABE.

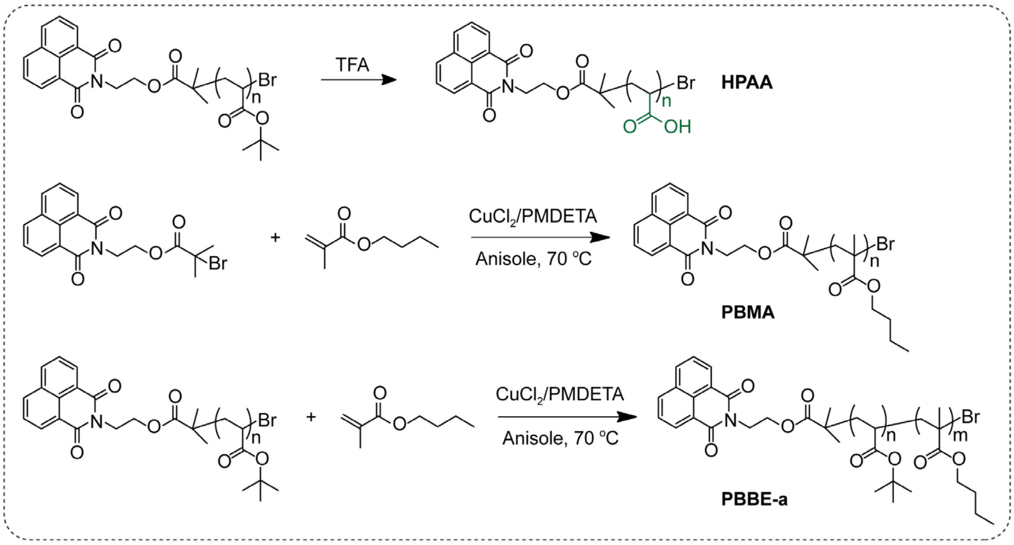

Supplementary Figure 3. Synthetic routes of control polymers HPAA, PBMA, and PBBE-a.

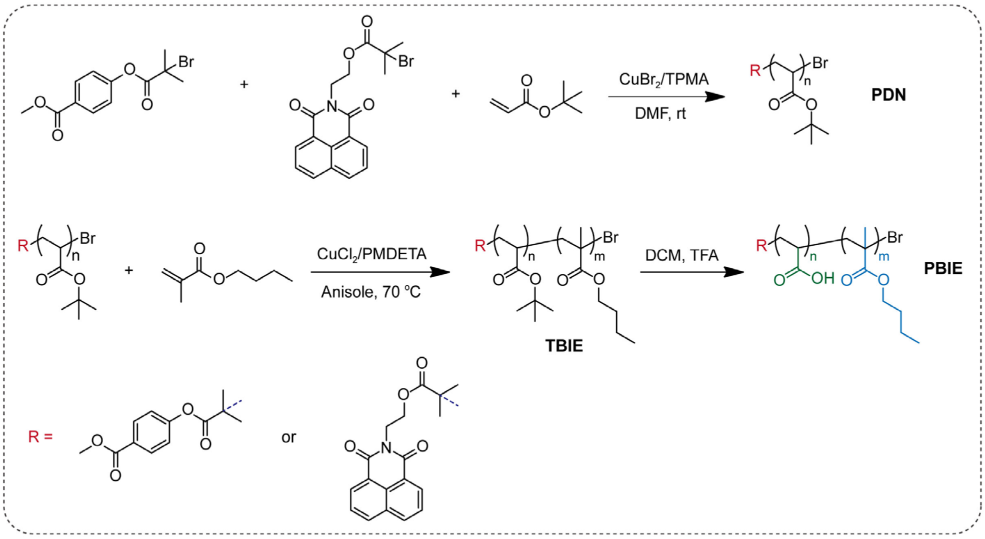

Supplementary Figure 4. Synthetic routes of ATRP macroinitiator PDN, and binary initiator system PBIE.

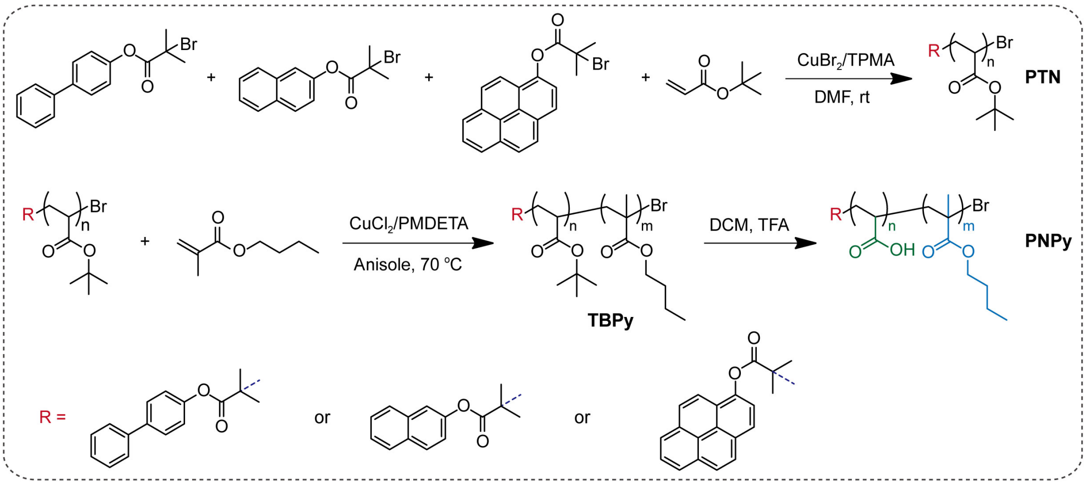

Supplementary Figure 5. Synthetic routes of ATRP macroinitiator PTN, and ternary initiator system PNPy.

Materials synthesis and characterizations

Synthesis of ATRP initiators

Herein, the ATRP initiator also acts as a phosphorescence chromophore. Therefore, we have chosen a set of aromatic units that contain hydroxy, carbonyl, and amide. These units containing hetero atoms (N and O) with lone-pair electrons can facilitate the ISC process to effectively populate triplet excitons. Moreover, these chromophores possess relatively rigid and planar molecular skeletons that are favorable for reducing molecular motions for suppressing non-radiative decay. Meanwhile, the hydroxy on the molecular skeleton can act as a reaction site with 2-bromoisobutyryl bromide, enabling the chromophore unit to convert to a chromophore initiator for the following ATRP process.

Methyl 4-((2-bromo-2-methylpropanoyl)oxy)benzoate (MBe). Methylparaben (2.50 g, 16.43 mmol) was dissolved in 60 mL extra dry dichloromethane and degassed by nitrogen for 20 min. After adding triethylamine (2.00 g, 19.72 mmol) and 2-bromoisobutyryl bromide (6.80 g, 29.57 mmol) in an ice bath, the solution was stirred for 2 h at room temperature. The solvent was removed by rotary evaporation, and the residue was purified by column chromatography to give MBe (4.60 g, 92.93%) as a white solid. ^{1}H NMR (500 MHz, $CDCl_3$) δ 8.10 (d, 2H), 7.22 (d, 2H), 3.92 (s, 3H), 2.07 (s, 6H). ^{13}C NMR (126 MHz, $CDCl_3$) δ 169.77, 166.25, 154.33, 131.26, 128.11, 121.16, 55.08, 52.28, 30.55. LC-MS (m/z): $[M+H]^+$ calcd. for $C_{12}H_{13}BrO_4$, 300.00; found, 301.04. Elemental analysis (calcd., found for $C_{12}H_{13}BrO_4$): C (47.86, 47.80), H (4.35, 4.34).

[1,1'-biphenyl]-4-yl 2-bromo-2-methylpropanoate (MBi). 4-Phenylphenol (2.00 g, 11.75 mmol) was dissolved in 50 mL extra dry dichloromethane and degassed by nitrogen for 20 min. After adding triethylamine (1.43 g, 14.10 mmol) and 2-bromoisobutyryl bromide (4.86 g, 21.15 mmol) in an ice bath, the solution was stirred for 2 h at room temperature. The solvent was removed by rotary evaporation and the residue was purified by column chromatography to give MBi (3.50 g, 93.31%) as a white solid. ^{1}H NMR (500 MHz, $CDCl_3$) δ 7.64-7.59 (m, 2H), 7.57 (dt, 2H), 7.47-7.41 (m, 2H), 7.38-7.32 (m, 1H), 7.23-7.17 (m, 2H), 2.09 (s, 6H). ^{13}C NMR (126 MHz, $CDCl_3$) δ 170.35, 150.21, 140.28, 139.37, 128.85, 128.26, 127.47, 127.17, 121.34, 55.41, 30.68. LC-MS (m/z): $[M]^+$ calcd. for $C_{16}H_{15}BrO_2$, 318.03; found, 318.21. Elemental analysis (calcd., found for $C_{16}H_{15}BrO_2$): C (60.21, 60.21), H (4.74, 4.80).

Nnaphthalen-2-yl 2-bromo-2-methylpropanoate (PNa). 2-Naphthol (1.80 g, 12.49 mmol) was dissolved in 40 mL extra dry dichloromethane and degassed by nitrogen for 20 min. After adding triethylamine (1.52 g, 14.98 mmol) and 2-bromoisobutyryl bromide (5.17 g, 22.48 mmol) in an ice bath, the solution was stirred for 2 h at room temperature. The solvent was removed by rotary evaporation, and the residue was purified by column chromatography to give PNa (3.20 g, 87.43%) as a white solid. ^{1}H NMR (500 MHz, $CDCl_3$) δ 7.93-7.84 (m, 2H), 7.82 (dd, 1H), 7.60 (d, 1H), 7.55-7.45 (m, 2H), 7.26 (dd, 1H), 2.11 (s, 6H). ^{13}C NMR (126 MHz, $CDCl_3$) δ 170.48, 148.41, 133.72, 131.62, 129.59, 127.77, 126.72, 125.91, 120.42, 118.19, 55.46, 30.71. LC-MS (m/z): $[M]^+$ calcd. for $C_{14}H_{13}BrO_2$, 292.01; found, 292.56. Elemental analysis (calcd., found for $C_{14}H_{13}BrO_2$): C (57.36, 57.41), H (4.47, 4.49).

2-(1,3-dioxo-1H-benzo[de]isoquinolin-2(3H)-yl)ethyl 2-bromo-2-methylpropanoate (DBI). 1,8-Naphthalic

anhydride (2.00 g, 10.09 mmol) and ethanolamine (1.54 g, 25.23 mmol) were dissolved in 100 mL ethanol. After stirring at 70 °C for 4 h, the mixture was cooled to room temperature and poured into deionized water (200 mL). Next, the precipitate was washed with ethanol and dried under a vacuum to give the intermediate product BHy as a light-yellow solid (1.50 g, 61.73%). ^{1}H NMR (500 MHz, $CDCl_3$) δ 8.59 (d, 2H), 8.21 (d, 2H), 7.75 (t, 2H), 4.46 (dd, 2H), 3.99 (dd, 2H). ^{13}C NMR (126 MHz, $CDCl_3$) δ 165.09, 134.25, 131.53, 128.19, 126.99, 122.38, 61.84, 42.81. Then, BHy (1.50 g, 6.22 mmol) was dissolved in 60 mL extra dry tetrahydrofuran and degassed by nitrogen for 20 min. After adding triethylamine (0.76 g, 7.46 mmol) and 2-bromoisobutyryl bromide (2.57 g, 11.20 mmol) in an ice bath, the solution was stirred at 45 °C overnight. The solvent was removed by rotary evaporation, and the residue was purified by column chromatography to give DBI (2.00 g, 82.30%) as a white solid. ^{1}H NMR (500 MHz, $CDCl_3$) δ 8.60 (d, 2H), 8.23 (d, 2H), 7.83-7.69 (m, 2H), 4.60-4.46 (m, 4H), 1.88 (s, 6H). ^{13}C NMR (126 MHz, $CDCl_3$) δ 171.60, 164.25, 134.19, 131.67, 131.40, 128.26, 127.01, 122.46, 63.34, 55.89, 38.76, 30.71. LC-MS (m/z): $[M+H]^+$ calcd. for $C_{18}H_{16}BrNO_4$, 389.03; found, 390.12. Elemental analysis (calcd., found for $C_{18}H_{16}BrNO_4$): C (55.40, 55.41), H (4.13, 4.19), N (3.59, 3.53).

Pyren-2-yl 2-bromo-2-methylpropanoate (MPy). 1-Hydroxypyrene (2.00 g, 9.16 mmol) was dissolved in 60 mL extra dry DMF and then degassed by nitrogen for 20 min. After adding triethylamine (1.11 g, 11.00 mmol) and 2-bromoisobutyryl bromide (3.79 g, 16.49 mmol) in an ice bath, the solution was stirred at 45 °C overnight. The solvent was removed by rotary evaporation, and the residue was purified by column chromatography to give MPy (2.50 g, 74.40%) as a light-yellow solid. ^{1}H NMR (500 MHz, $CDCl_3$) δ 8.27-8.20 (m, 4H), 8.16 (d, 1H), 8.10 (s, 2H), 8.06 (t, 1H), 7.83 (d, 1H), 2.29 (s, 6H). ^{13}C NMR (126 MHz, $CDCl_3$) δ 170.70, 144.08, 131.13, 130.97, 129.57, 128.43, 127.31, 127.07, 126.40, 125.62, 125.40, 124.99, 124.49, 123.28, 120.02, 119.13, 55.51, 30.96. LC-MS (m/z): $[M+H]^+$ calcd. for $C_{20}H_{15}BrO_2$, 366.03; found, 367.11. Elemental analysis (calcd., found for $C_{20}H_{15}BrO_2$): C (65.41, 65.39), H (4.12, 4.13).

Synthesis of macroinitiators

PDN

The macroinitiator PDN was prepared in the binary initiator system by ATRP. DBI (5.00 mg, 12.81 μmol, 1 eq), MBe (0.116 g, 0.385 mmol, 30 eq), TBA (15.27 g, 119.14 mmol, 9300 eq), $CuBr_2$ (2.66 mg, 0.012 mmol, 0.93 eq), TPMA (13.84 mg, 0.048 mmol, 3.72 eq) and DMF (6.5 mL) were degassed by purging with nitrogen for 30 mins. Polymerization commenced upon the addition of a piece of Cu(0) to the degassed reaction mixture. The solution was stirred at room temperature for 16 h and then precipitated in $MeOH/H_2O$ mixtures to give the macroinitiator PDN. M_n:25080; PD:1.09.

PTN

The macroinitiator PTN was prepared in the ternary initiator system by ATRP. MPy (6.00 mg, 16.34 μmol, 1 eq), PNa (0.019 g, 0.065 mmol, 4 eq), MBi (0.083g, 0.261 mmol, 16 eq), filtered TBA (13.19 g, 102.93 mmol, 6300 eq), $CuBr_2$ (2.30 mg, 10.29 μmol, 0.63 eq), TPMA (11.90 mg, 0.041 mmol, 2.52 eq) and DMF (5.5 mL) were degassed by purging with nitrogen for 30 mins. Polymerization commenced upon the addition of a piece of Cu(0) to the degassed reaction mixture. The solution was stirred at room temperature for 16 h and then precipitated in $MeOH/H_2O$ mixtures to give the macroinitiator PTN. M_n:25150; PD:1.10.

Synthesis of block copolymers

PAMA, PAME and PAPE

The block copolymers were synthesized through the next chain extension reactions according to Supplementary Figures 2-5. For synthesizing PAMA, macroinitiator PBM (1.5 g, 0.057 mmol, 1 eq), methyl methacrylate (MMA, 2.27 g, 22.67 mmol, 400 eq), $CuCl_2$ (4.60 mg, 0.034 mmol, 0.6 eq), and pentamethyldiethylenetriamine (PMDETA, 11.85 mg, 0.068 mmol, 1.2 eq) were dissolved in anisole (4 mL). The mixture was degassed by purging with nitrogen for 20 mins. After adding degassing stannous octoate (13.86 mg, 0.034 mmol, 0.6 eq), the mixture was stirred at 70 °C for 20 h. Upon cooling, the catalyst was removed by passing a solution of the polymer in THF over a neutral alumina plug, and then the filtered polymer solution was precipitated in MeOH/H_2O mixtures to give the as-prepared PBAE block copolymer (M_n: 56510; PD: 1.25). Finally, the polymer PBAE was dissolved in DCM (80 mL), and 10 mL TFA was slowly added to the mixture. After stirring at room temperature for 24 h, the solution was removed by rotary evaporation to yield the amphiphilic block copolymer PAMA film, which was further heated under a vacuum at 65 °C for 12 h to obtain the dried polymer film. To obtain regular specimens for photophysical and mechanical tests, the above polymer film was further hot pressed in a mold at 120 °C. Following the same synthetic procedure, PAME and PAPE copolymers were prepared with ethyl methacrylate (EMA) and propyl methacrylate (PMA) as the second block monomers.

PBIE

The macroinitiator PDN (1.2 g, 0.048 mmol, 1 eq), BMA (3.41 g, 24.00 mmol, 500 eq), $CuCl_2$ (3.90 mg, 0.029 mmol, 0.6 eq), and PMDETA (10.05 mg, 0.058 mmol, 1.2 eq) were dissolved in anisole (5 mL). The mixture was degassed by purging with nitrogen for 20 mins. After adding degassing stannous octoate (11.75 mg, 0.029 mmol, 0.6 eq), the mixture was stirred at 70 °C for 20 h. Upon cooling, the catalyst was removed by passing a solution of the polymer in THF over a neutral alumina plug, and then the filtered polymer solution was precipitated in MeOH/H_2O mixtures to give the TBIE-1 block copolymer (M_n: 85270; PD: 1.22). Finally, polymer TBIE-1 was dissolved in DCM (80 mL), and 10 mL TFA was slowly added to the mixture. After stirring at room temperature for 24 h, the solution was removed by rotary evaporation to yield the amphiphilic block copolymer PBIE-1 film, which was further heated under vacuum at 65 °C for 12 h to obtain the dried polymer film. Following the same synthetic procedure as PBIE-1, copolymers PBIE-2 and PBIE-3 were prepared by changing the molar feed ratio of PDN/BMA to 1/1100 and 1/2000, respectively.

PNPy

The macroinitiator PTN (1.2 g, 0.048 mmol, 1 eq), BMA (3.41 g, 24.00 mmol, 500 eq), $CuCl_2$ (3.90 mg, 0.029 mmol, 0.6 eq), and PMDETA (10.05 mg, 0.058 mmol, 1.2 eq) were dissolved in anisole (5 mL). The mixture was degassed by purging with nitrogen for 20 mins. After adding degassing stannous octoate (11.75 mg, 0.029 mmol, 0.6 eq), the mixture was stirred at 70 °C for 20 h. Upon cooling, the catalyst was removed by passing a solution of the polymer in THF over a neutral alumina plug, and then the filtered polymer solution was precipitated in MeOH/H_2O mixtures to give the TBPy-1 block copolymer (M_n: 87090; PD: 1.14). Finally, polymer TBPy-1 was dissolved in DCM (80 mL), and 10 mL TFA was slowly added to the mixture. After stirring at room temperature for 24 h, the solution was removed by rotary evaporation to yield the amphiphilic block copolymer PNPy-1 film, which was further heated under vacuum at 65 °C for 12 h to obtain the dried polymer film. Following the same

synthetic procedure as PNPy-1, copolymers PNPy-2 and PNPy-3 were prepared by changing the molar feed ratio of PTN/BMA to 1/1100 and 1/2000, respectively.

Synthesis of control polymers

HPAA

Homopolymer HPAA was prepared by hydrolyzation of macroinitiator PBM. Polymer PBM (1 g) was dissolved in DCM (100 mL), and 15 mL TFA was slowly added to the mixture. After stirring at room temperature for 24 h, the solution was removed by rotary evaporation to yield HPAA film, which was further heated under a vacuum at 65 °C for 12 h to obtain the dried polymer film.

PBMA

DBI (0.03 g, 0.077 mmol, 1 eq), filtered BMA (8.75 g, 61.5 mmol, 800 eq), $CuCl_2$ (10.36 mg, 0.077 mmol, 1 eq), PMDETA (26.69 mg, 0.154 mmol, 2 eq) were dissolved in anisole (5 mL). The mixture was degassed by purging with nitrogen for 20 mins. After the addition of degassing stannous octoate (31.00 mg, 0.077 mmol, 1 eq), the mixture was stirred at 70 °C for 20 h. Upon cooling, the catalyst was removed by passing a solution of the polymer in THF over a neutral alumina plug. Then, the filtered polymer solution was precipitated in MeOH/H_2O mixtures to give homopolymer PBMA, which was further heated under vacuum at 65 °C for 12 h to obtain the dried polymer film. M_n: 99770; PD: 1.26.

PBBE-a

The macroinitiator PBM (1.2 g, 0.044 mmol, 1 eq), BMA (3.75 g, 26.40 mmol, 600 eq), $CuCl_2$ (3.55 mg, 0.026 mmol, 0.6 eq), and PMDETA (9.15 mg, 0.053 mmol, 1.2 eq) were dissolved in anisole (5 mL). The mixture was degassed by purging with nitrogen for 20 mins. After adding degassing stannous octoate (10.45 mg, 0.026 mmol, 0.6 eq), the mixture was stirred at 70 °C for 20 h. Upon cooling, the catalyst was removed by passing a solution of the polymer in THF over a neutral alumina plug. Then the filtered polymer solution was precipitated in MeOH/H_2O mixtures to give PBBE-a copolymer, which was further heated under vacuum at 65 °C for 12 h to obtain the dried polymer film. M_n: 105120; PD: 1.2.

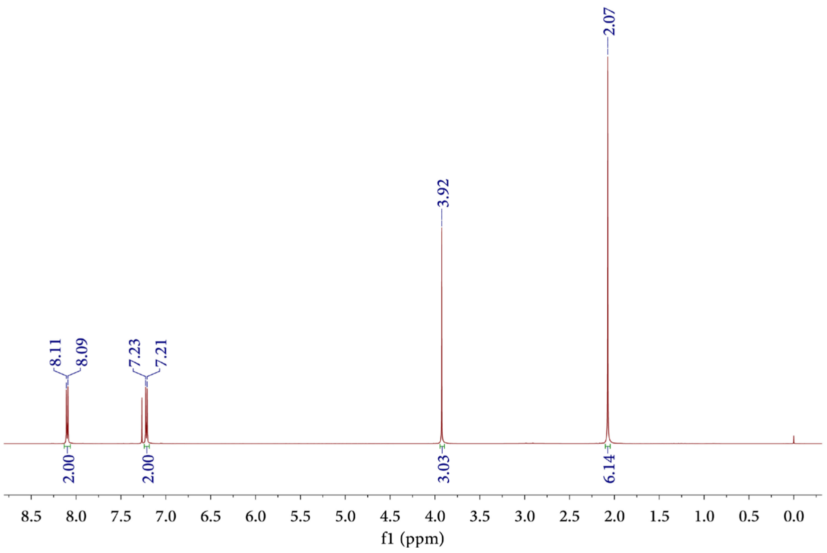

Supplementary Figure 6. ^1H NMR spectrum of MBe initiator in $CDCl_3$.

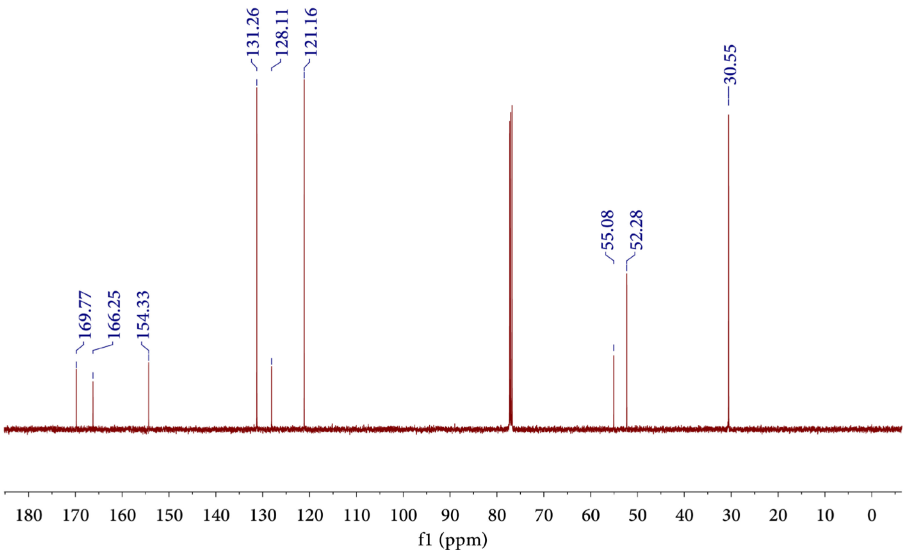

Supplementary Figure 7. ^{13}C NMR spectrum of MBe initiator in $CDCl_3$.

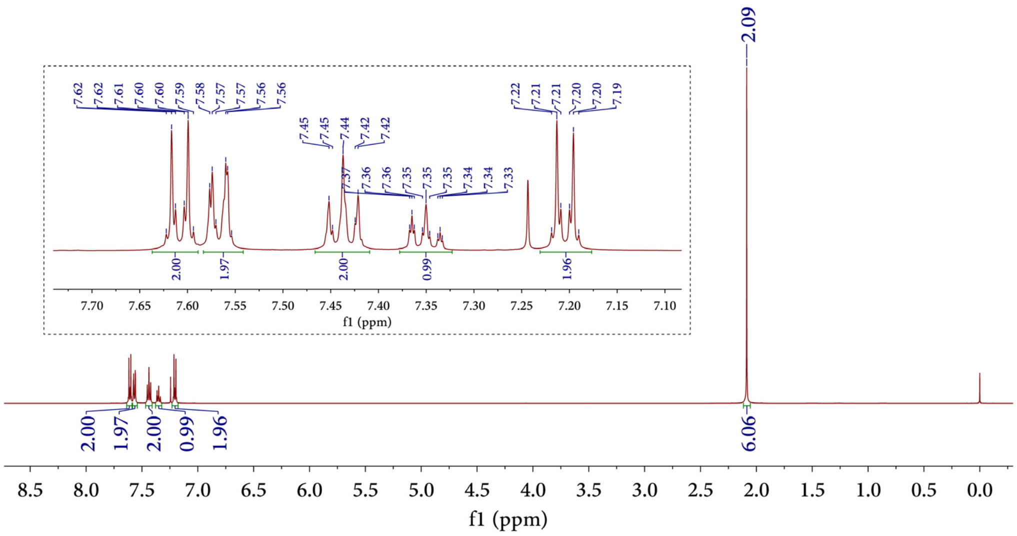

Supplementary Figure 8. ^{1}H NMR spectrum of MBi initiator in $CDCl_3$.

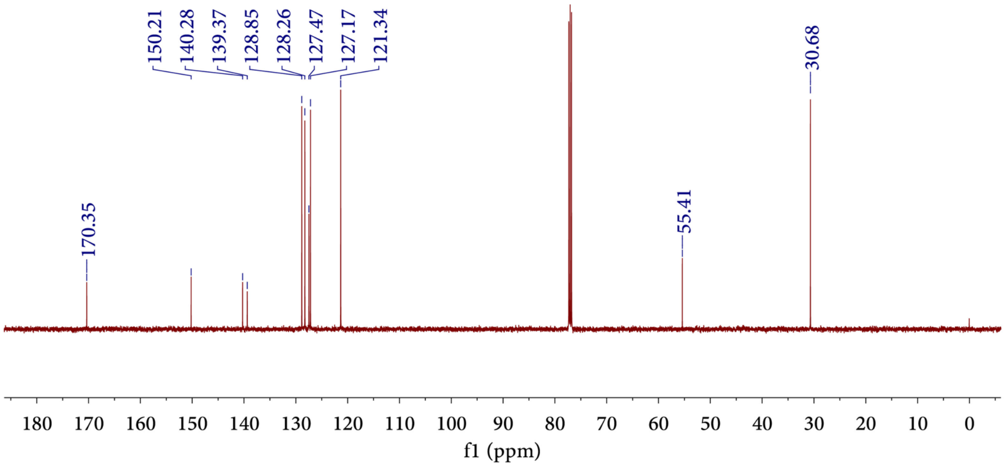

Supplementary Figure 9. ^{13}C NMR spectrum of MBi initiator in $CDCl_3$.

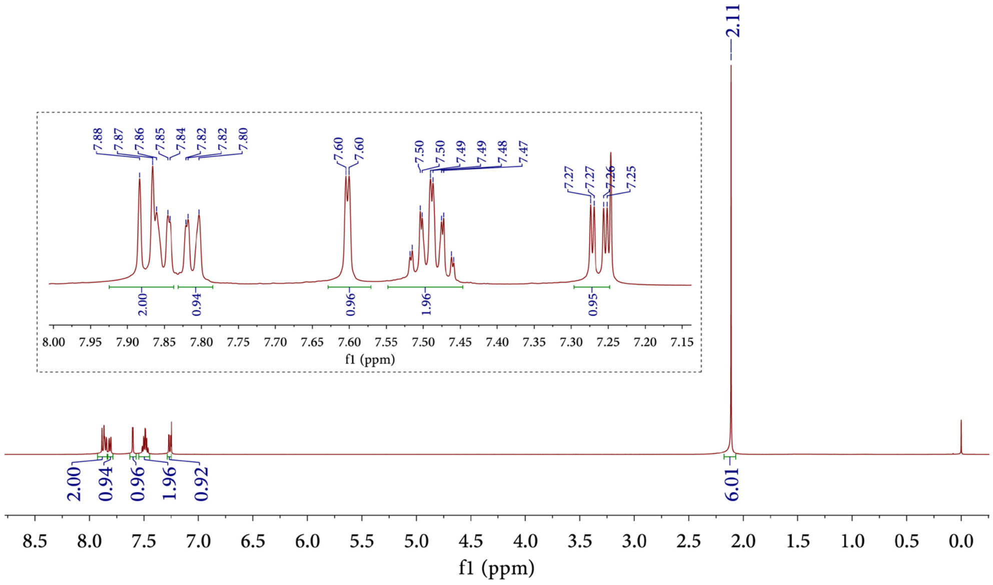

Supplementary Figure 10. ^{1}H NMR spectrum of PNa initiator in $CDCl_3$.

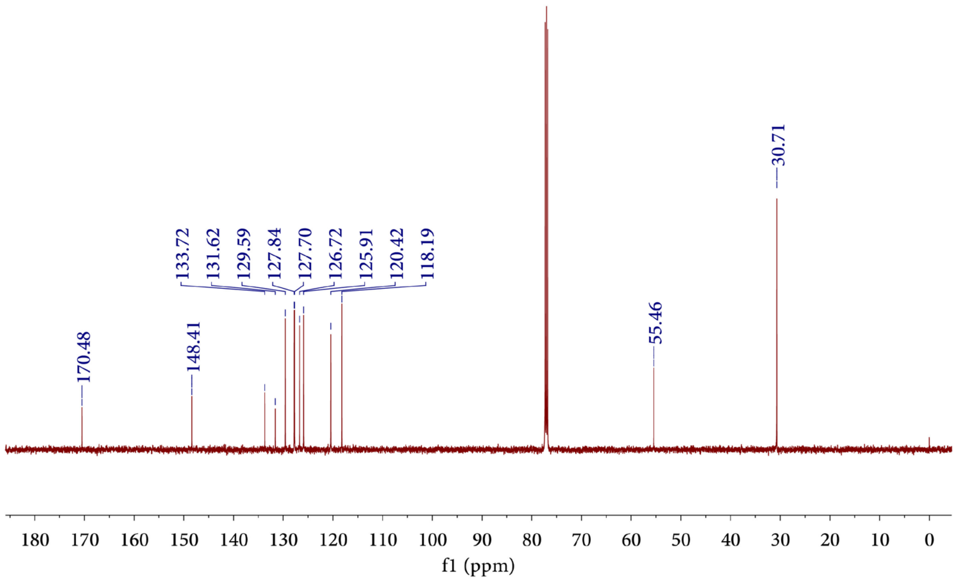

Supplementary Figure 11. ^{13}C NMR spectrum of PNa initiator in $CDCl_3$.

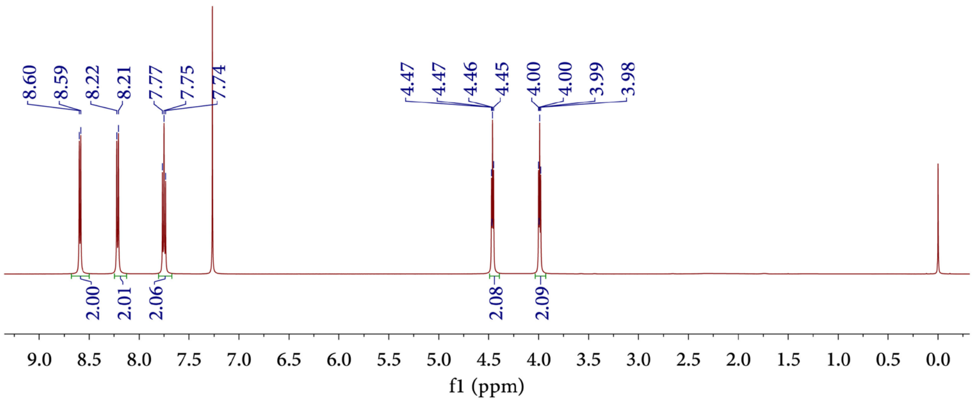

Supplementary Figure 12. ^{1}H NMR spectrum of compound BHy in $CDCl_3$.

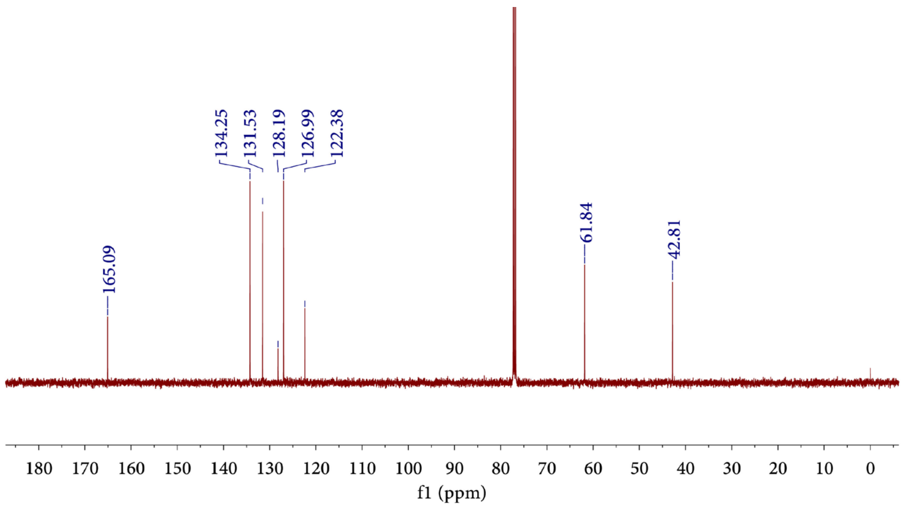

Supplementary Figure 13. ^{13}C NMR spectrum of compound BHy in $CDCl_3$.

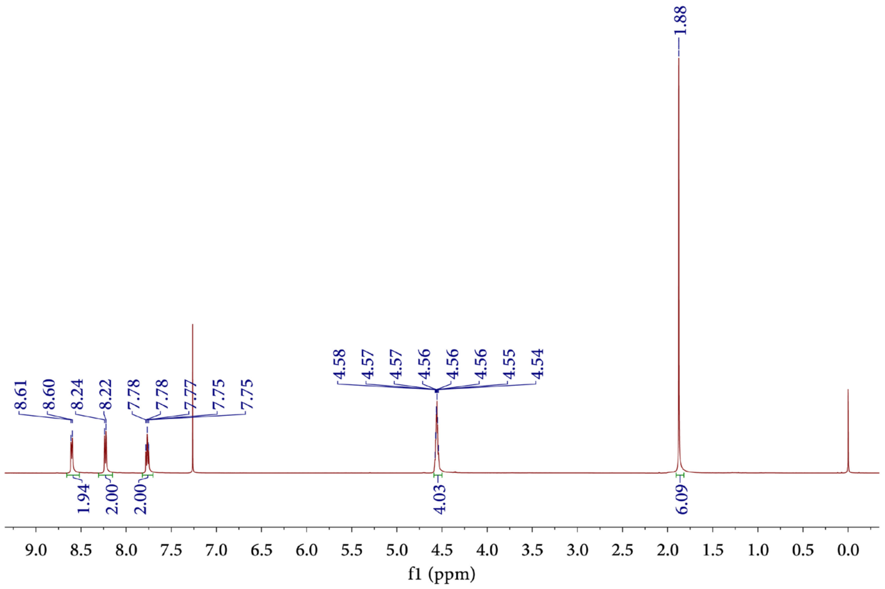

Supplementary Figure 14. ^{1}H NMR spectrum of DBI initiator in $CDCl_3$.

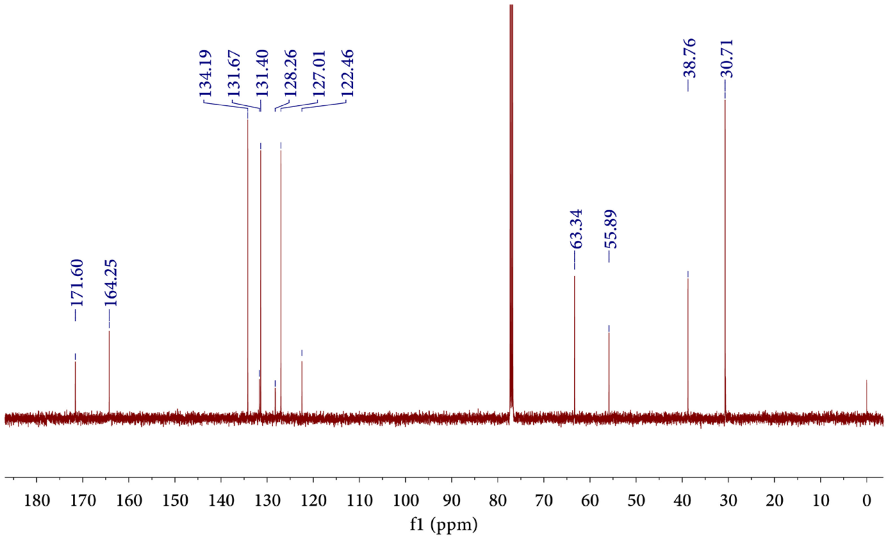

Supplementary Figure 15. ^{13}C NMR spectrum of DBI initiator in $CDCl_3$.

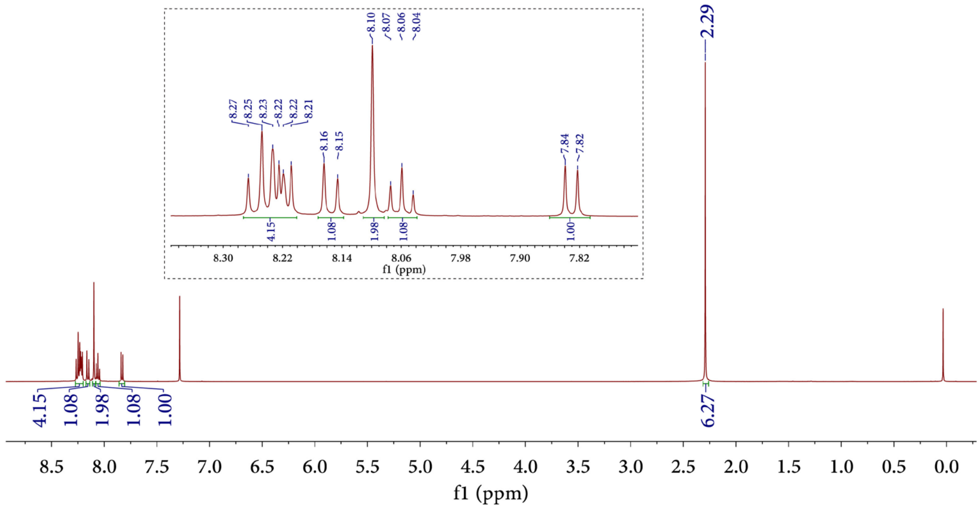

Supplementary Figure 16. ^{1}H NMR spectrum of MPy initiator in $CDCl_3$.

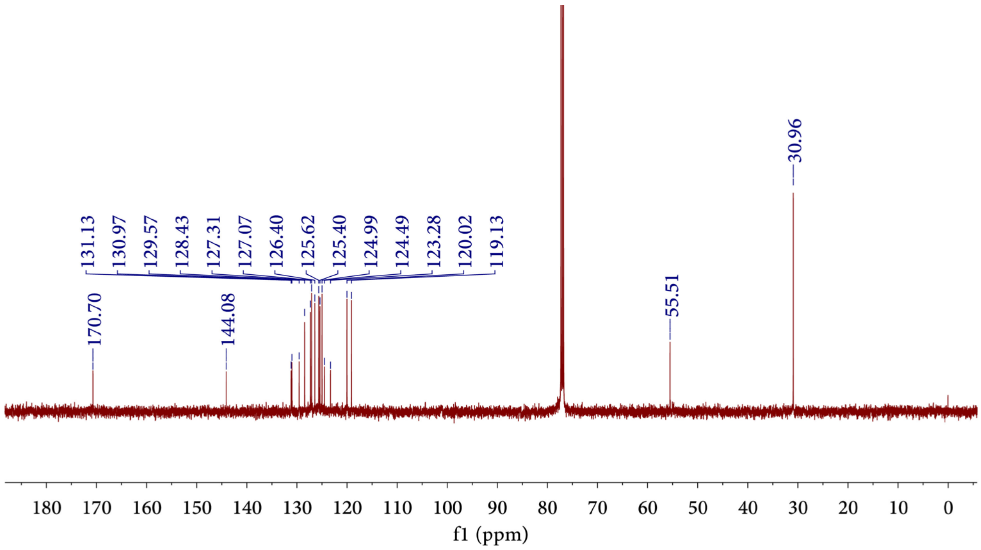

Supplementary Figure 17. ^{13}C NMR spectrum of MPy initiator in $CDCl_3$.

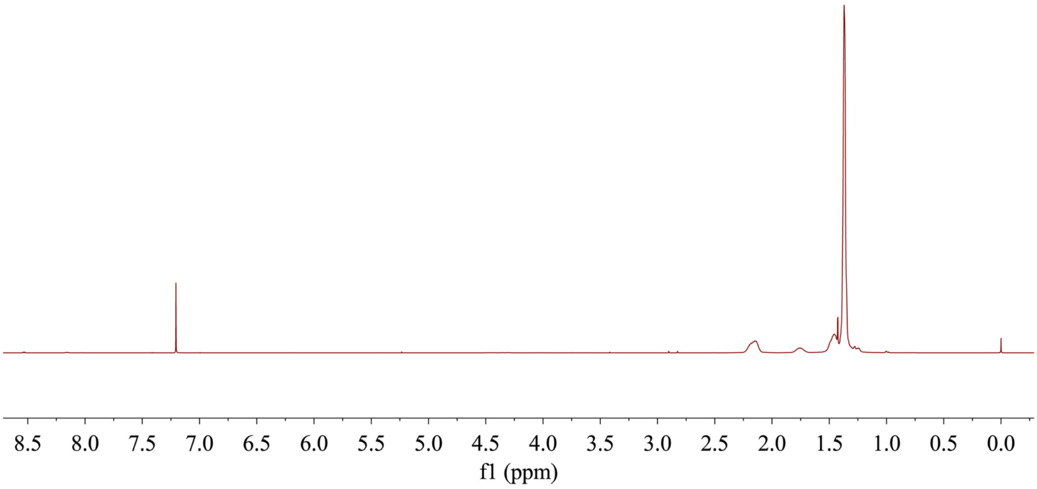

Supplementary Figure 18. ^{1}H NMR spectrum of PBM macroinitiator in $CDCl_3$.

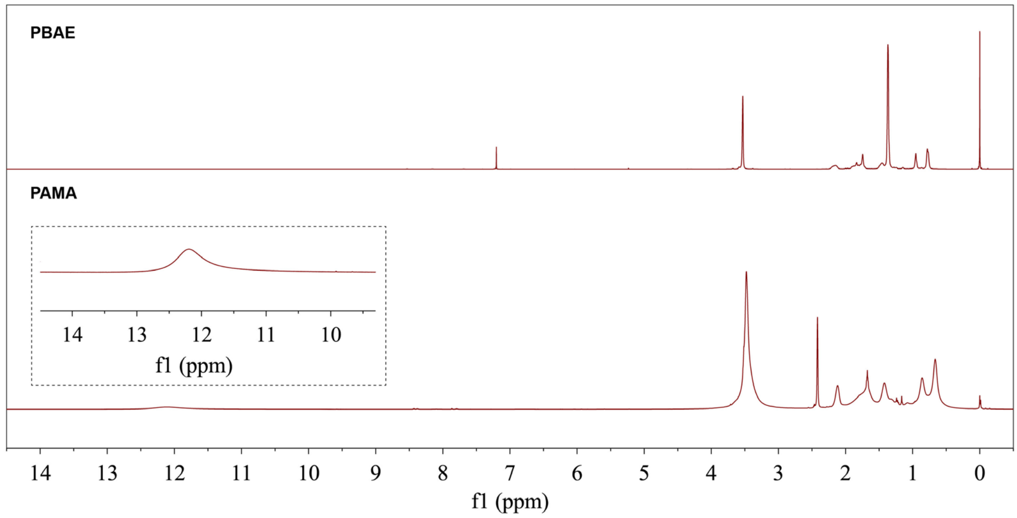

Supplementary Figure 19. ^{1}H NMR spectra of PBAE in $CDCl_3$ and PAMA in DMSO-d_6.

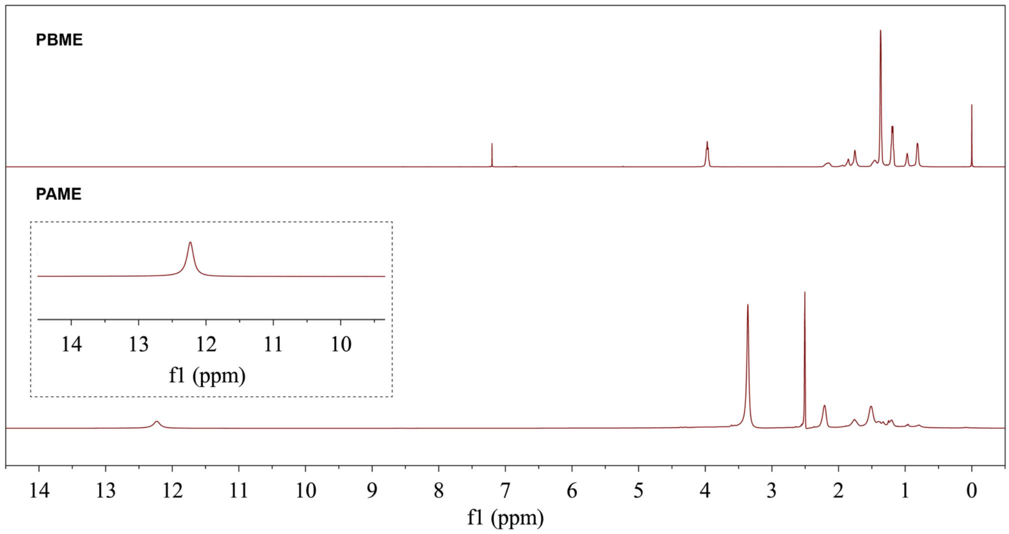

Supplementary Figure 20. ^{1}H NMR spectra of PBME in $CDCl_3$ and PAME in DMSO-d_6.

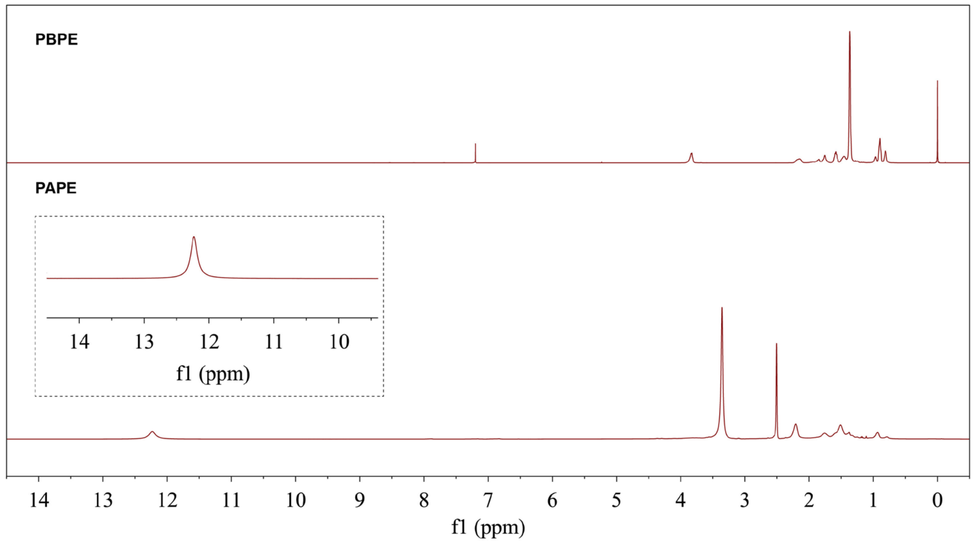

Supplementary Figure 21. ^{1}H NMR spectra of PBPE in $CDCl_3$ and PAPE in DMSO-d_6.

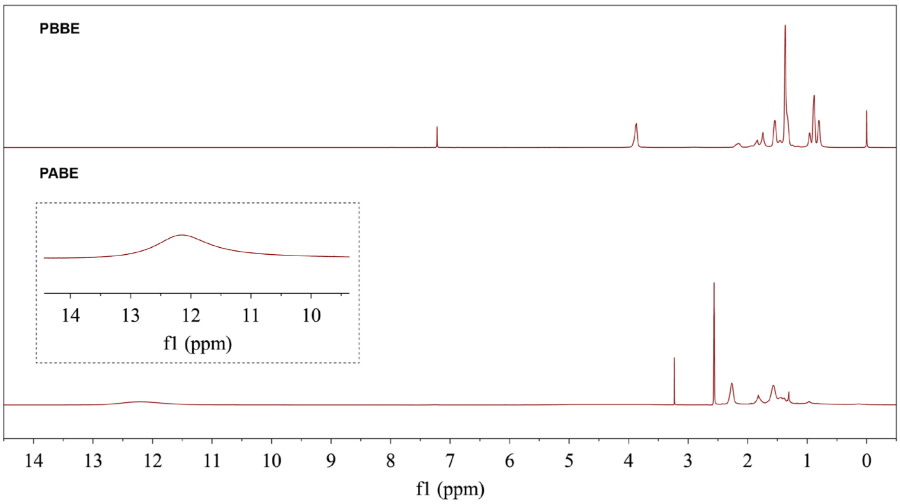

Supplementary Figure 22. ^{1}H NMR spectra of PBBE in $CDCl_3$ and PABE in DMSO-d_6.

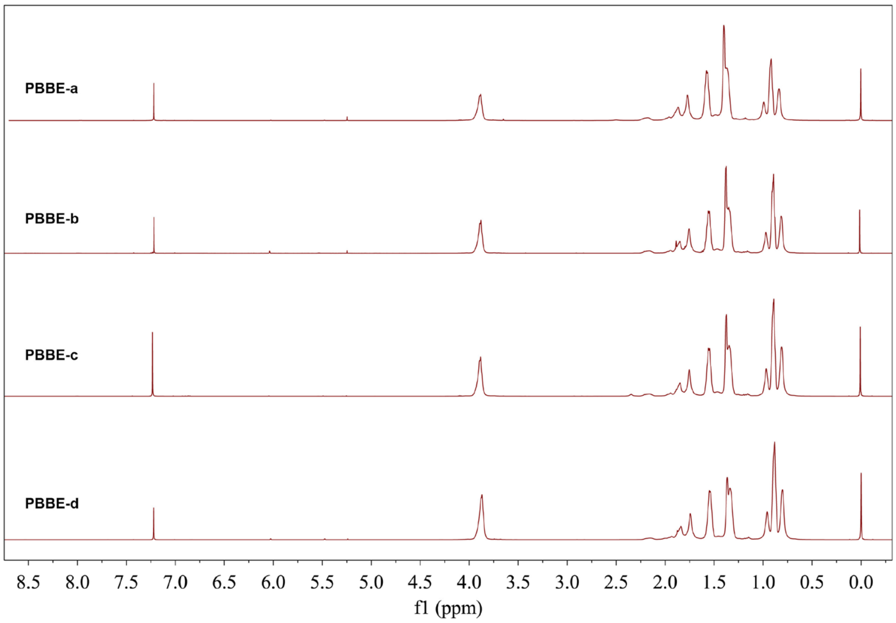

Supplementary Figure 23. ^{1}H NMR spectra of copolymers PBBE-a, PBBE-b, PBBE-c, and PBBE-d in $CDCl_3$.

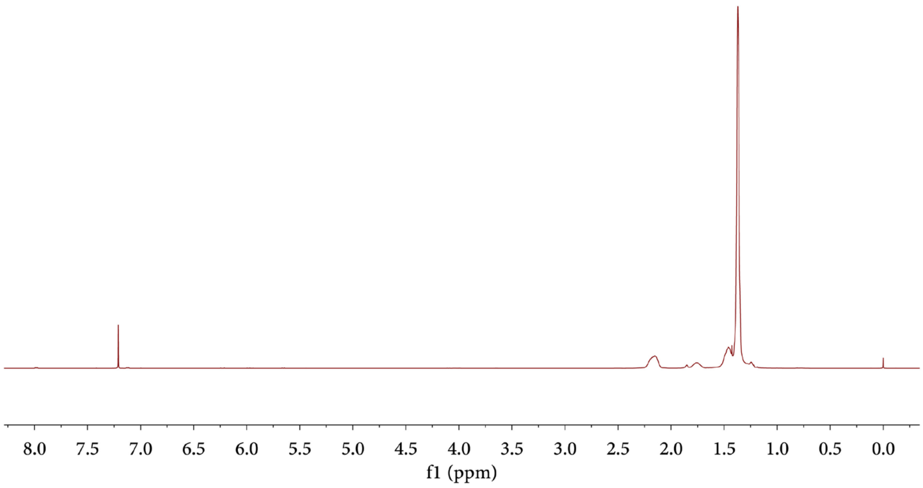

Supplementary Figure 24. ^{1}H NMR spectrum of PDN macroinitiator in $CDCl_3$.

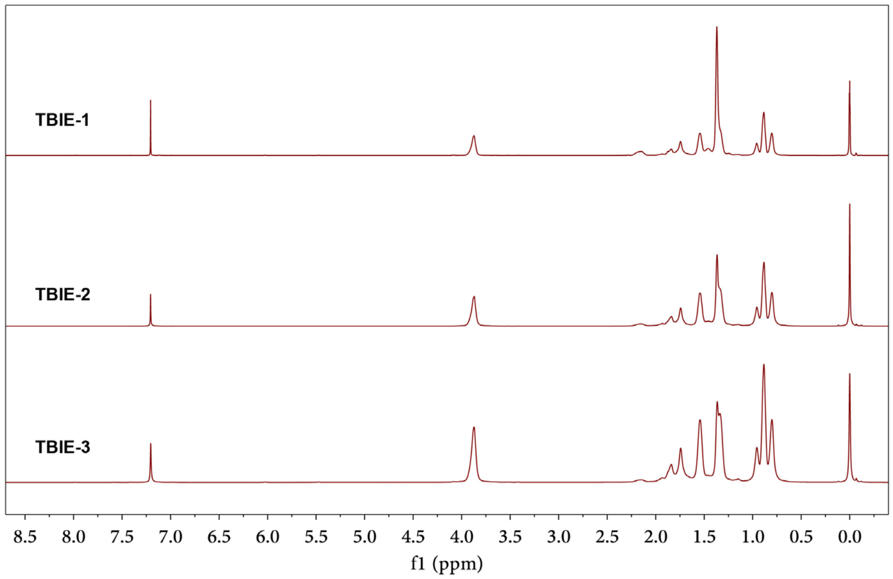

Supplementary Figure 25. ^{1}H NMR spectra of copolymers TBIE-1, TBIE-2, and TBIE-3 in $CDCl_3$.

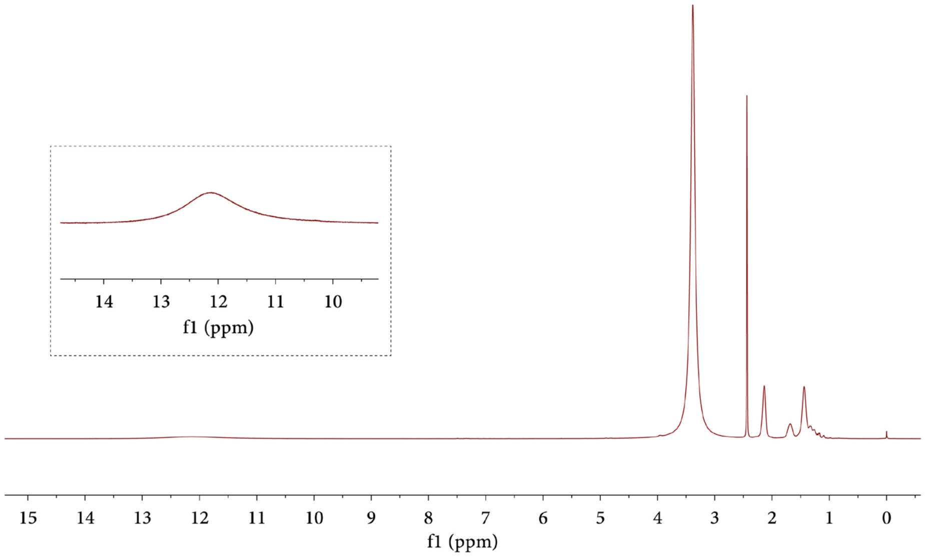

Supplementary Figure 26. ^{1}H NMR spectrum of amphipathic block copolymer PBIE-1 in DMSO-d_6.

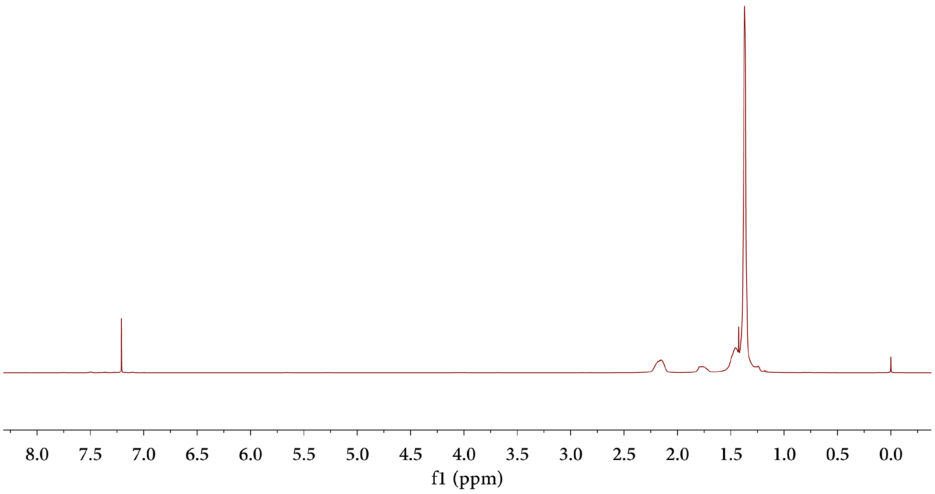

Supplementary Figure 27. ^{1}H NMR spectrum of PTN macroinitiator in $CDCl_3$.

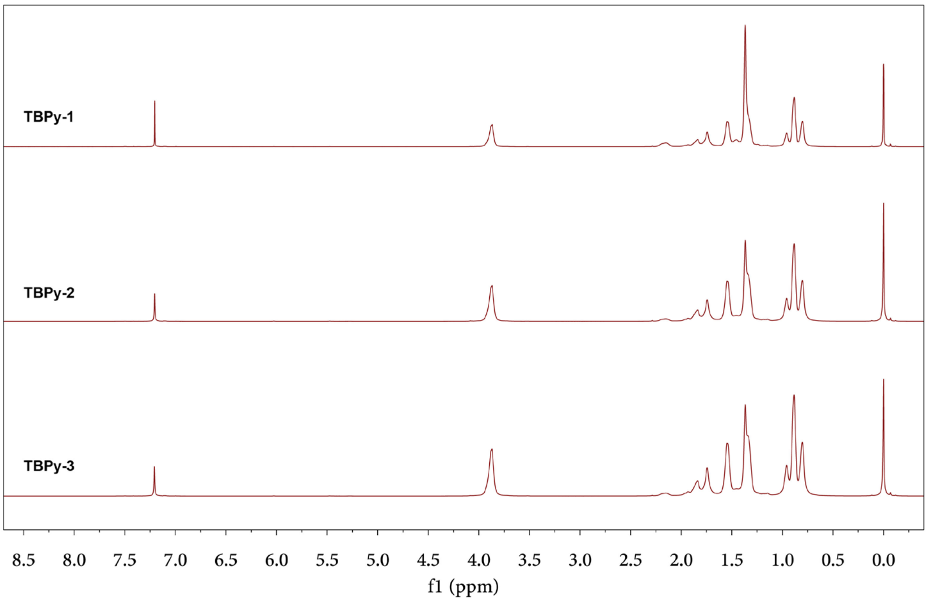

Supplementary Figure 28. ^1H NMR spectra of copolymers TBPy-1, TBPy-2, and TBPy-3 in $CDCl_3$.

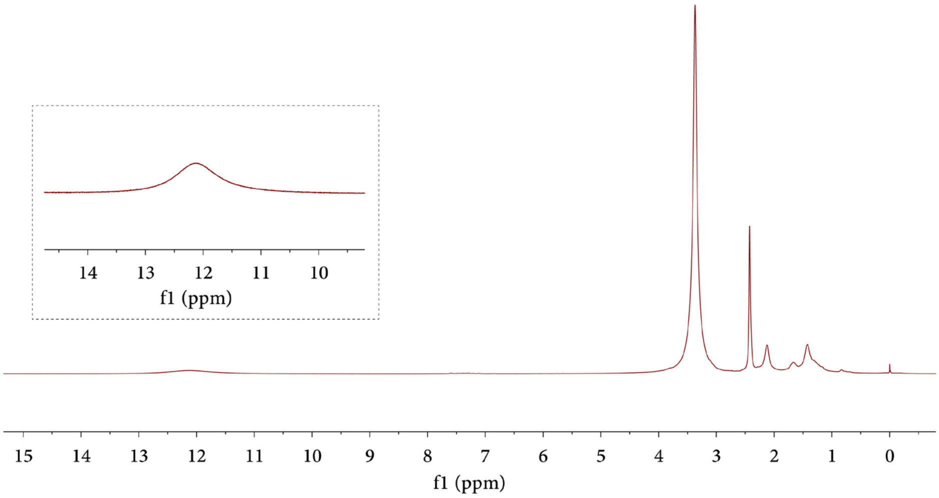

Supplementary Figure 29. ^1H NMR spectrum of amphipathic block copolymer PNPy-1 in DMSO-d_6.

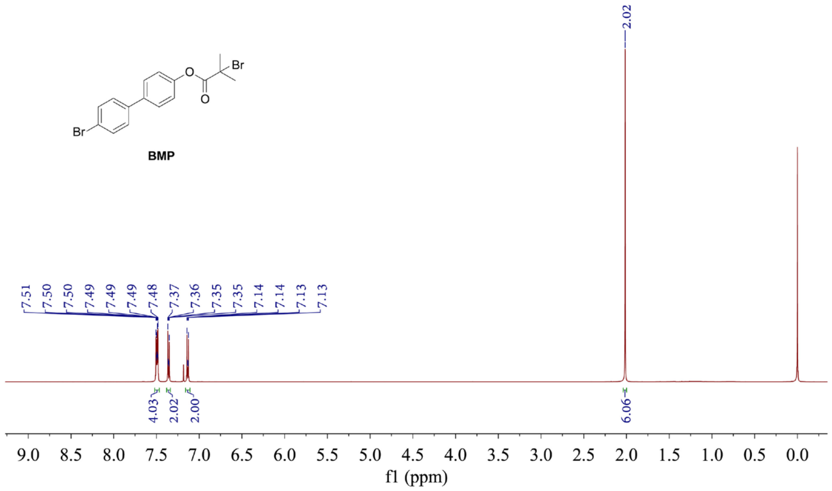

Supplementary Figure 30. Chemical structure of BMP initiator and the ^1H NMR spectrum in $CDCl_3$.

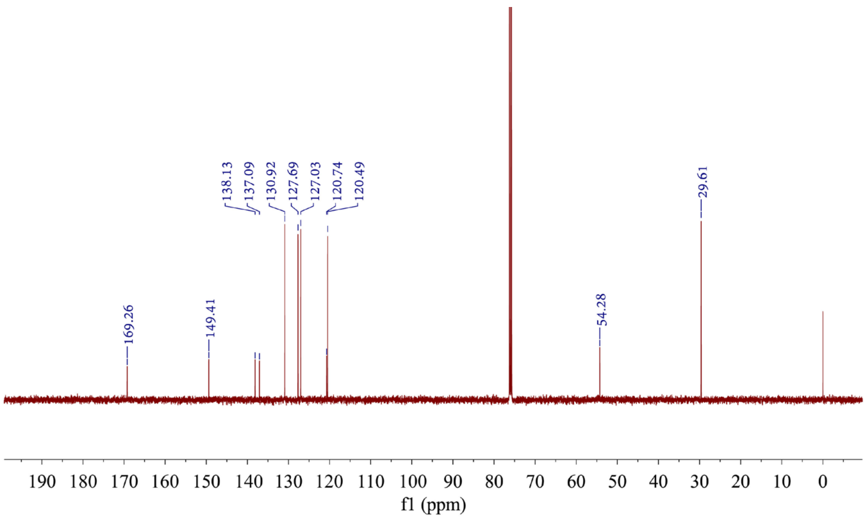

Supplementary Figure 31. ^{13}C NMR spectrum of BMP initiator in $CDCl_3$.

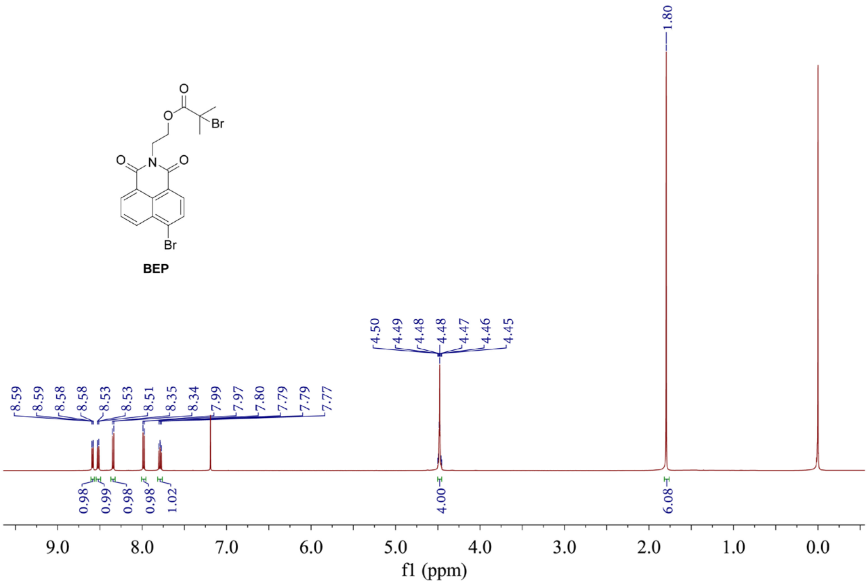

Supplementary Figure 32. Chemical structure of BEP initiator and the ^{1}H NMR spectrum in $CDCl_3$.

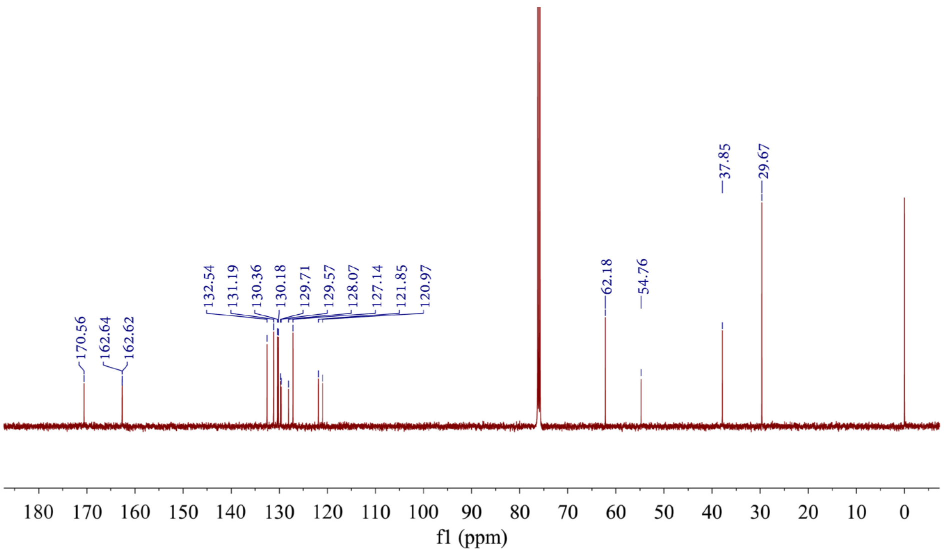

Supplementary Figure 33. ^{13}C NMR spectrum of BEP initiator in $CDCl_3$.

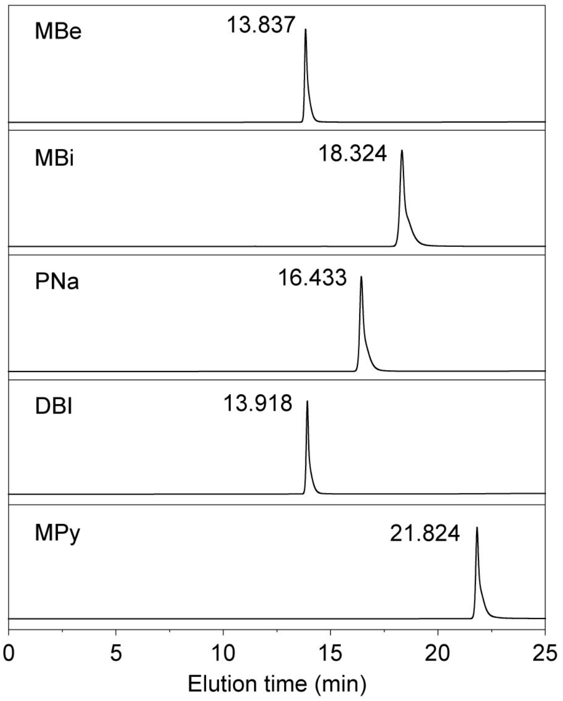

Supplementary Figure 34. High-performance liquid chromatogram spectra of MBe, MBi, PNa, DBI, and MPy initiators in acetonitrile solution.

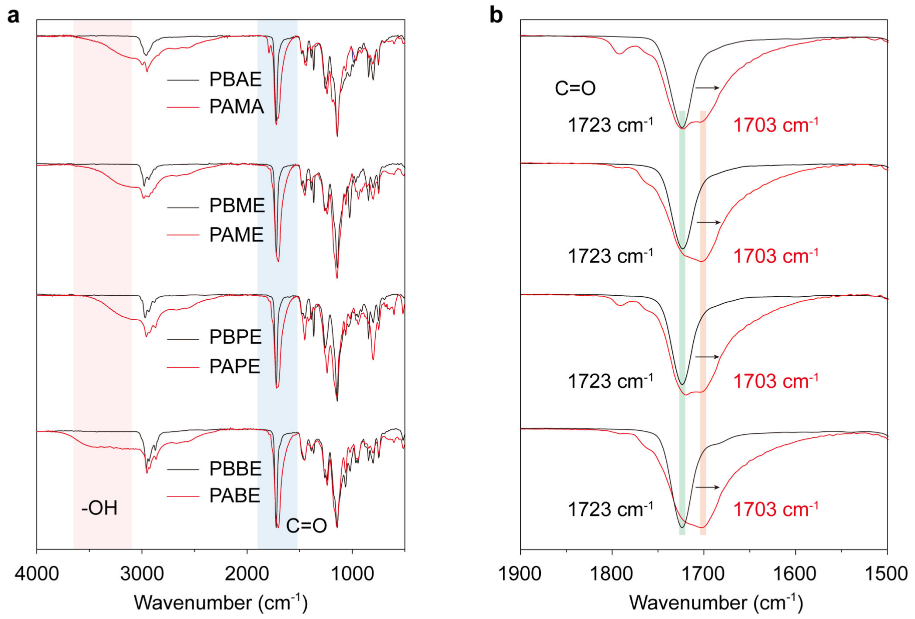

Supplementary Figure 35. (a) FTIR spectra of unhydrolyzed copolymers PBAE, PBME, PBPE, and PBBE (black lines), and hydrolyzed block copolymers PAMA, PAME, PAPE, and PABE (red lines). The characteristic peaks of the hydroxy group at around 3100-3600 cm^{-1} indicate that the *tert*-butyl ester groups were converted into carboxyl moieties after hydrolyzation. (b) The shift of carbonyl stretching resonance for copolymers before (black lines) and after (red lines) hydrolyzation demonstrates the formation of C=O···H–O hydrogen bonds.

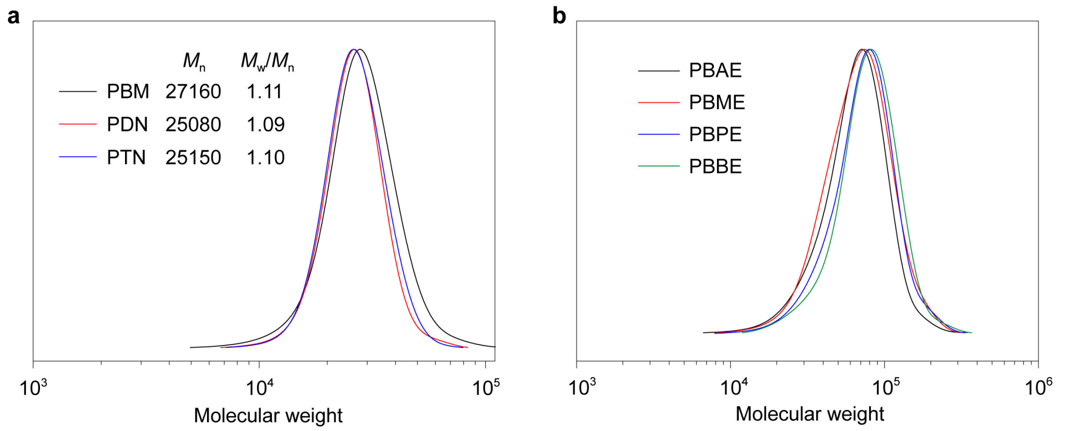

Supplementary Figure 36. GPC traces of (a) macroinitiators PBM, PDN, and PTN, and (b) copolymers PBAE, PBME, PBPE, and PBBE.

Supplementary Table 1. GPC characterizations of copolymers PBAE to PBBE. n and m represent the degree of polymerization for each block, which was calculated by M_n.

Sample	PBAE	PBME	PBPE	PBBE
M_w	71110	75520	83060	85230
M_n	56510	59060	61320	68800
PD	1.25	1.28	1.29	1.24
n	210	210	210	210
m	293	280	267	293
n/m	1:1.4	1:1.3	1:1.3	1:1.4

Supplementary Table 2. GPC characterizations of copolymers PBBEs. n and m represent the degree of polymerization for each block, which was calculated by M_n.

Sample	PBBE-a	PBBE-b	PBBE-c	PBBE-d
M_w	126660	185330	265440	328840
M_n	105120	163290	222620	283800
PD	1.20	1.13	1.19	1.16
n	210	210	210	210
m	548	958	1375	1805
n/m	1:2.6	1:4.6	1:6.6	1:8.6

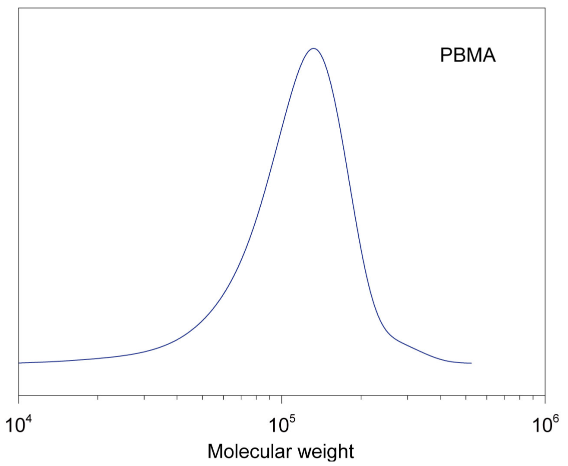

Supplementary Figure 37. GPC trace of control polymer PBMA. M_n: 99770, M_w/M_n: 1.26.

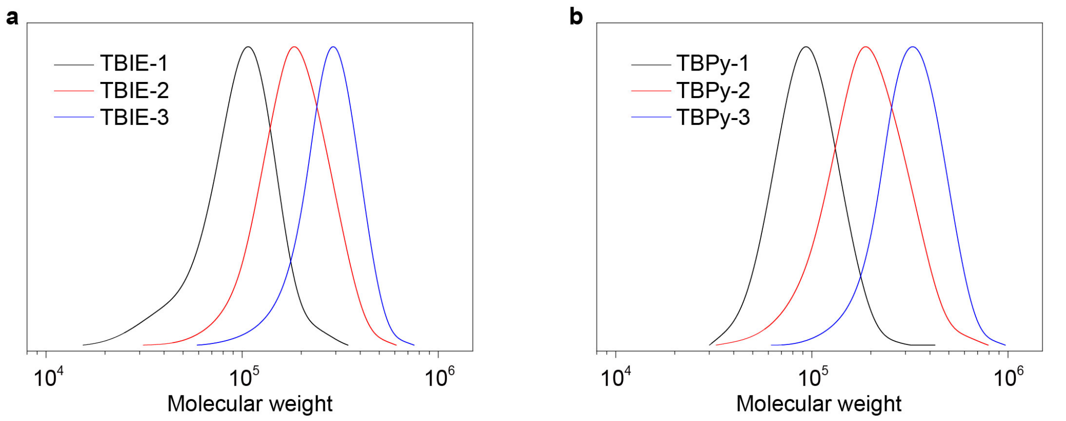

Supplementary Figure 38. GPC traces of copolymers TBIEs (a) and TBPys (b).

Supplementary Table 3. GPC characterizations of TBIEs and TBPys.

Sample	TBIE-1	TBIE-2	TBIE-3	TBPy-1	TBPy-2	TBPy-3
M_w	104300	198260	294000	99560	209250	342190
M_n	85270	168350	261630	87090	168850	298350
PD	1.22	1.18	1.12	1.14	1.24	1.15

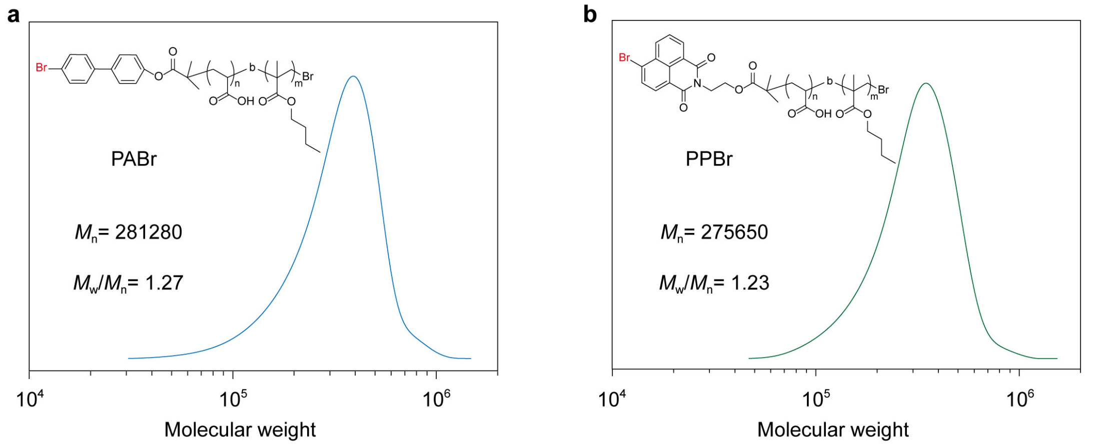

Supplementary Figure 39. Chemical structures and GPC traces of block copolymers PABr and PPBr.

II. Supplementary Discussion

2.1 Additional photophysical and mechanical characterizations

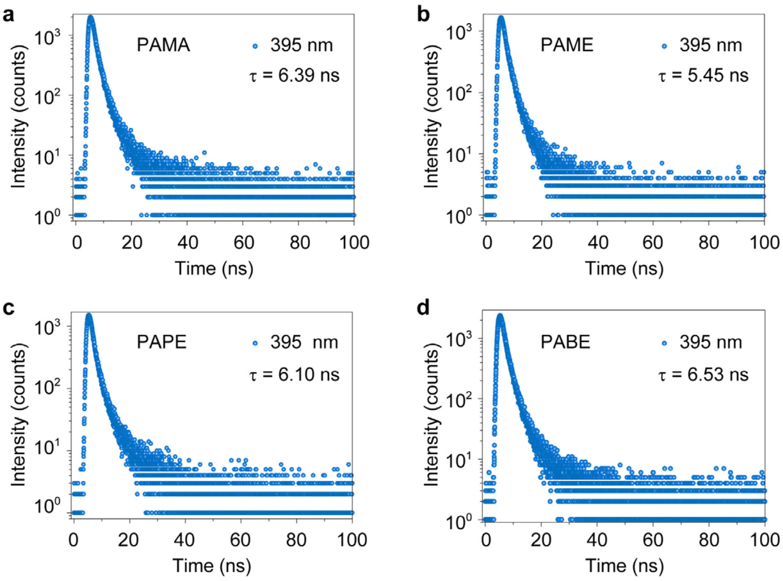

Supplementary Figure 40. Lifetime profiles of the emission bands at 395 nm for PAMA to PABE films.

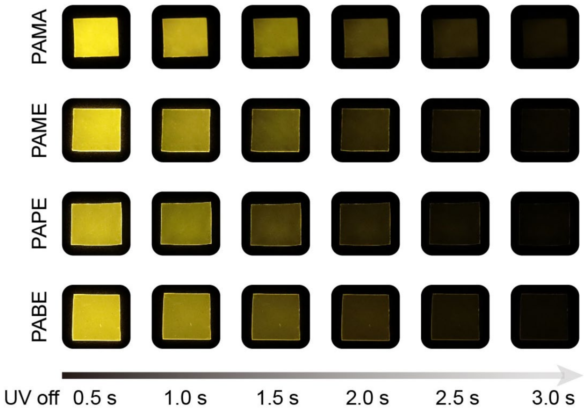

Supplementary Figure 41. Ultralong phosphorescence photographs of block copolymers PAMA, PAME, PAPE, and PABE after turning off the 365 nm UV lamp.

Supplementary Table 4. Luminescence lifetimes (τ) of PAMA, PAME, PAPE, and PABE films under ambient conditions.[a]

Polymer	Wavelength (nm)	Fluorescence				Phosphorescence	
		τ_1 (ns)	A_1 (%)	τ_2 (ns)	A_2 (%)	τ (ms)	A (%)
PAMA	395	1.68	84.07	6.39	15.93		
	548					232.02	100%
PAME	395	1.62	82.23	5.45	17.77		
	548					223.86	100%
PAPE	395	1.53	78.15	6.10	21.85		
	548					232.25	100%
PABE	395	1.72	85.97	6.56	14.03		
	548					231.36	100%

[a] Determined from the fitting function of $I(t) = A_1e^{-t/\tau1} + A_2e^{-t/\tau2}$ according to the luminescence decay curves.

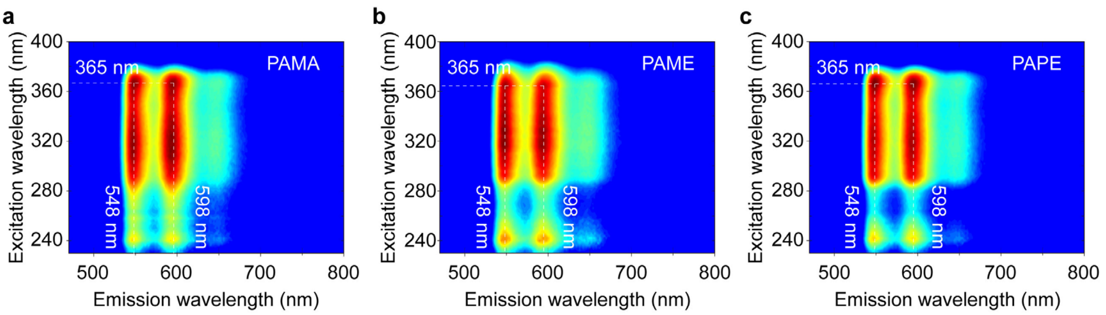

Supplementary Figure 42. Excitation-phosphorescence mappings of amphiphilic block copolymers PAMA, PAME, and PAPE under ambient conditions, respectively.

To reveal the effect of terminal bromine atom at the polymer chain on phosphorescence performance, we prepared another control polymer NPAA (without bromine atoms) through conventional radical polymerization of acrylic acid (AA) and naphthalimide (NA) using 2-azoisobutyronitrile (AIBN) as an initiator. As shown in Supplementary Figures 43-44, polymer NPAA displayed similar photophysical properties to polymer HPAA prepared by ATRP, including PL and RTP emission wavelengths, lifetimes, and quantum efficiencies, respectively. Therefore, the terminal bromine atom at the polymer chain has an insignificant influence on the RTP performance of the copolymer.

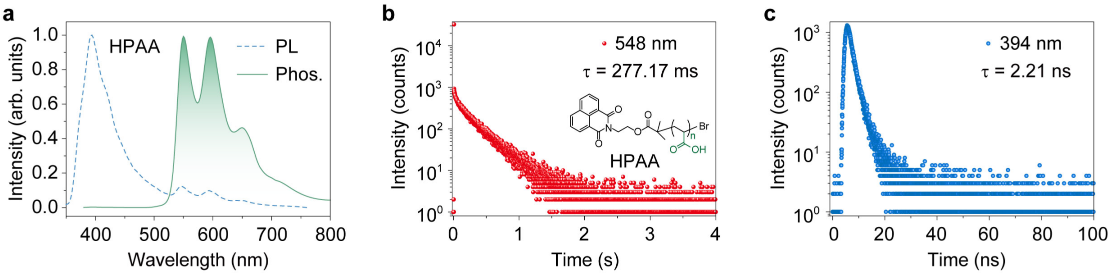

Supplementary Figure 43. (a) Normalized steady-state photoluminescence (PL) and phosphorescence (Phos.) spectra of HPAA film under 340 nm excitation. (b) Lifetime decay curve of emission band at 548 nm. (c) Lifetime profile of emission band at 394 nm.

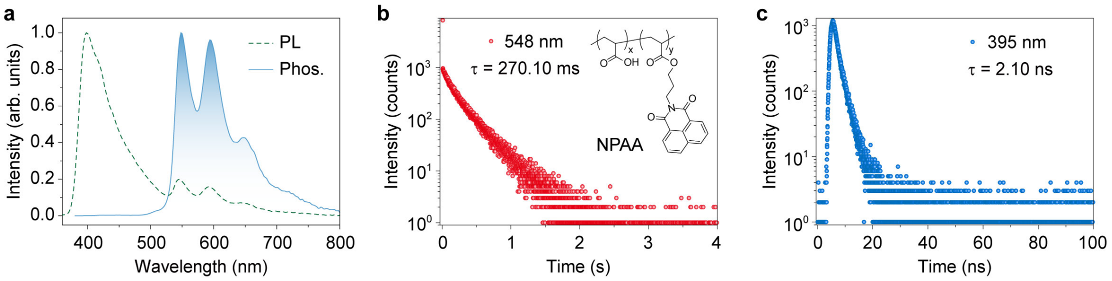

Supplementary Figure 44. (a) Normalized steady-state photoluminescence (PL) and phosphorescence (Phos.) spectra of NPAA film under 340 nm excitation. (b) Lifetime decay curve of emission band at 548 nm. (c) Lifetime profile of emission band at 395 nm.

Supplementary Table 5. Photoluminescence efficiencies of PAMA, PAME, PAPE, and PABE films under ambient conditions.

Polymers	$\lambda_{Fluor.}$ [nm]	$\Phi_{Fluor.}$ [%]	$\lambda_{Phos.}$ [nm]	$\Phi_{Phos.}$ [%]
PAMA	395	11.18	548	1.62
PAME	395	17.54	548	1.96
PAPE	395	9.89	548	1.21
PABE	395	15.90	548	2.20

Supplementary Table 6. Photophysical parameters of control polymers NPAA and HPAA under ambient conditions.

Polymer	$\lambda_{Fluor.}$ [nm]	$\tau_{Fluor.}$ [ns]	$\Phi_{Fluor.}$ [%]	$\lambda_{Phos.}$ [nm]	$\tau_{Phos.}$ [ms]	$\Phi_{Phos.}$ [%]
NPAA	395	2.10	11.43	548	270.10	3.17
HPAA	394	2.21	15.12	548	277.17	2.88

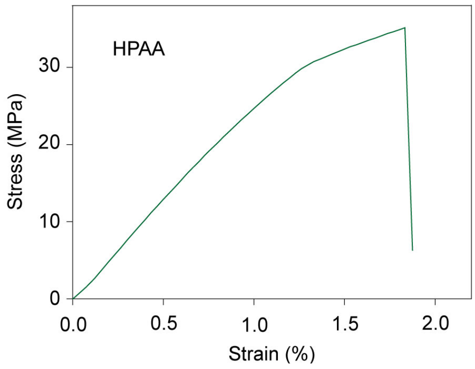

Supplementary Figure 45. Stress-strain curve of control polymer HPAA film.

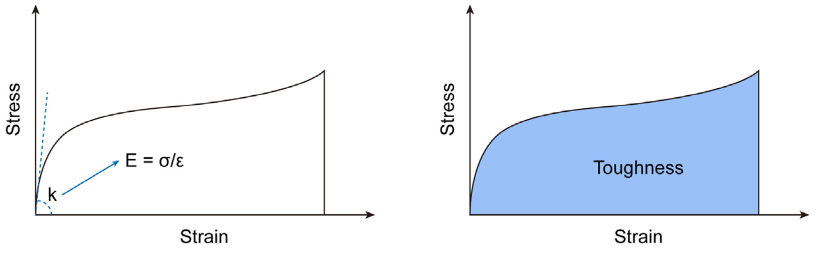

Supplementary Figure 46. Illustration of the calculation for Young's modulus and toughness values.

Supplementary Table 7. Summary of mechanical properties of PAMA, PAME, PAPE, and PABE copolymers with different second block structures and control polymer HPAA.

Sample	Young's modulus (MPa)	Strain-at-break (%)	Strength-at-break (MPa)	Toughness (MJ m^{-3})
PAMA	676.6	10.5	48.7	3.2
PAME	514.0	51.9	31.4	16.1
PAPE	283.2	100.3	26.5	21.2
PABE	112.8	188.0	14.4	23.6
HPAA	2529.2	1.8	35.1	0.4

Note: Young's modulus ($E = \sigma/\varepsilon$) is calculated from the low-strain region (<5% strain) of the stress-strain curve. Herein, we select the σ value corresponding to the ε at 5%. Except for HPAA with strain-at-break below 5%, we select its σ value with ε at 1%. The toughness is calculated by the integral area of the stress-strain curves.

Upon increasing the alkyl side chain length, the intermolecular distance and free volume of polymer chains increased, and the molecular interaction weakened, thus the molecular motility increased, which resulted in decreased glass transition temperature and much-improved deformability of corresponding polymer films (Supplementary Figure 47)[1-3]. Besides, reducing the steric hindrance of the side group would decrease the block of molecular chain internal rotation, resulting in a lower T_g. Additionally, reducing the number of asymmetric substituents would lower the inside rotating barrier and reduce the T_g of the polymer.

To reduce the T_g further, we introduced hexyl methacrylate with a longer flexible alkyl side chain and butyl acrylate with lower steric hindrance to prepare the soft block. The copolymer structures, T_g values, and photophysical properties were further investigated (Supplementary Figures 48-49). Compared with copolymer PABE, polymers PAHE and PABA displayed further reduced T_g of -5 and -54 °C, respectively, and identical phosphorescence emission centers at 548 nm. However, the phosphorescence lifetimes dramatically decreased from 231.36 ms in PABE to 208.59 ms in PAHE and 158.36 ms in PABA films, respectively. Because a too-low T_g will cause intense polymer chain motions at room temperature, which will induce strong non-radiative transition and weaken the phosphorescence. Therefore, choosing a soft block with a suitable T_g is crucial for reducing the non-radiative loss from the soft polymeric motions and maintaining the good optical properties of copolymers. Based on these data, the soft block with a T_g close to room temperature may be beneficial to achieving RTP copolymers with simultaneously long-lived emission and decent stretchability.

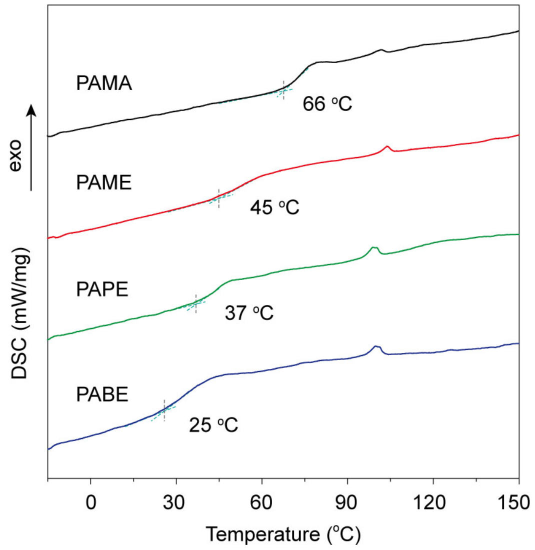

Supplementary Figure 47. DSC curves of amphiphilic block copolymers PAMA, PAME, PAPE, and PABE.

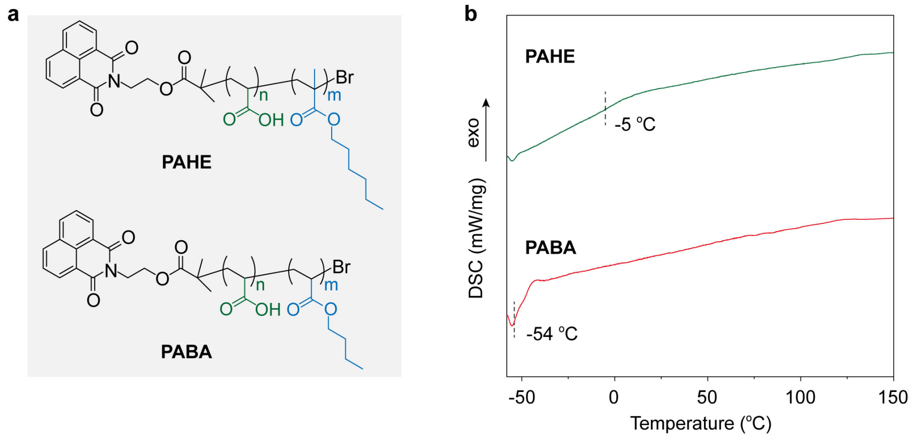

Supplementary Figure 48. (a) Chemical structures and (b) DSC curves of copolymers PAHE and PABA.

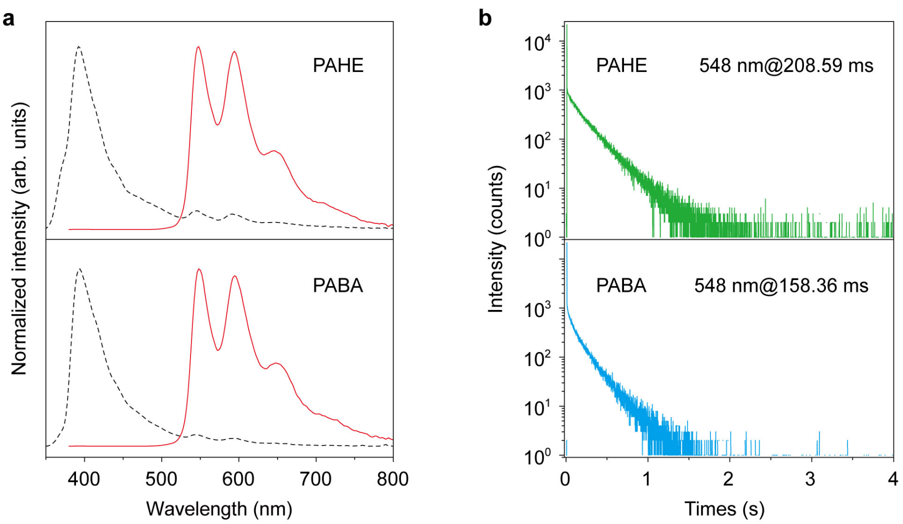

Supplementary Figure 49. (a) Photoluminescence (black lines) and phosphorescence (red lines) spectra and (b) Lifetime profiles of emission bands at 548 nm for copolymers PAHE and PABA under ambient conditions.

The living character of ATRP allows for precise control of molecular weight, giving rise to further tuning the mechanical performance of block copolymers. Considering the superior stretchability and simultaneous ultralong phosphorescence of PABE, we further prepared PABE-a to PABE-d films with varied molecular weights to systematically investigate the effect of the mass fraction of the soft blocks on the photophysical and mechanical properties. It was found that the mechanical properties, including elongation, toughness, and Young's modulus of the copolymers all depended on the content of soft blocks (Supplementary Table 11). A longer soft segment (in the range of M_n=300 KDa) can offer more effective strain dissipation and, thus, higher deformability, without sacrificing ultralong RTP lifetimes.

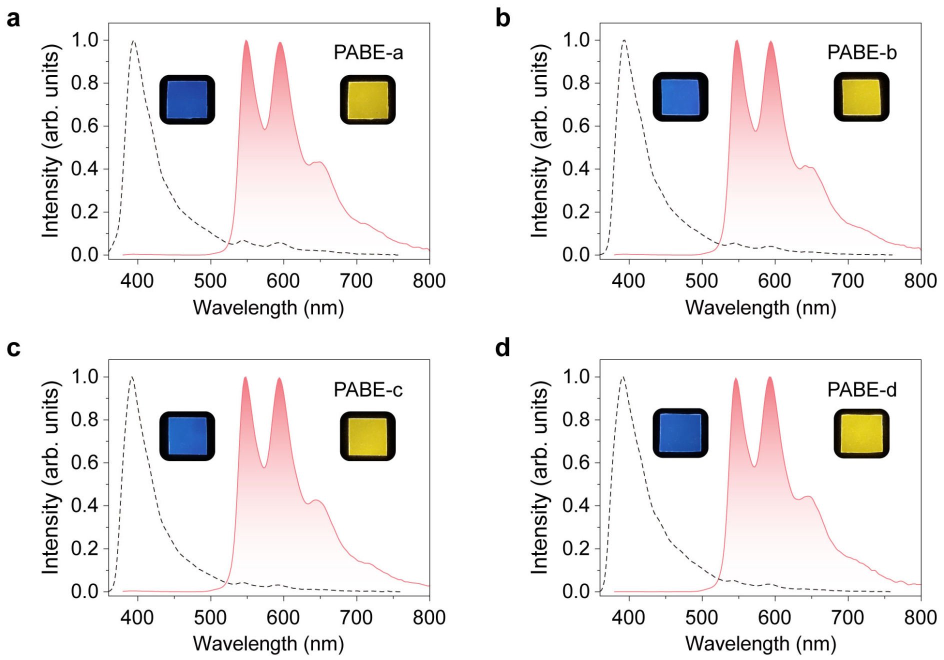

Supplementary Figure 50. Normalized steady-state photoluminescence (black lines) and phosphorescence (red lines) spectra of PABE-a to PABE-d films, respectively. Insets are the corresponding luminescence photographs under the 365 nm UV lamp on (right) and off (left).

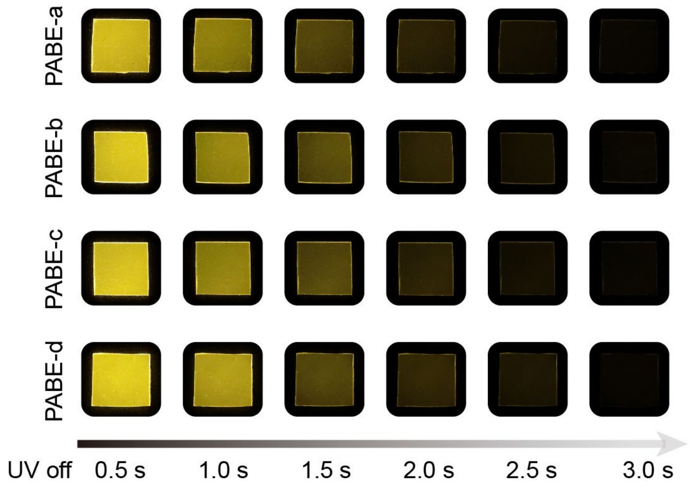

Supplementary Figure 51. The ultralong phosphorescence photographs of PABE-a to PABE-d films after turning off the 365 nm UV lamp.

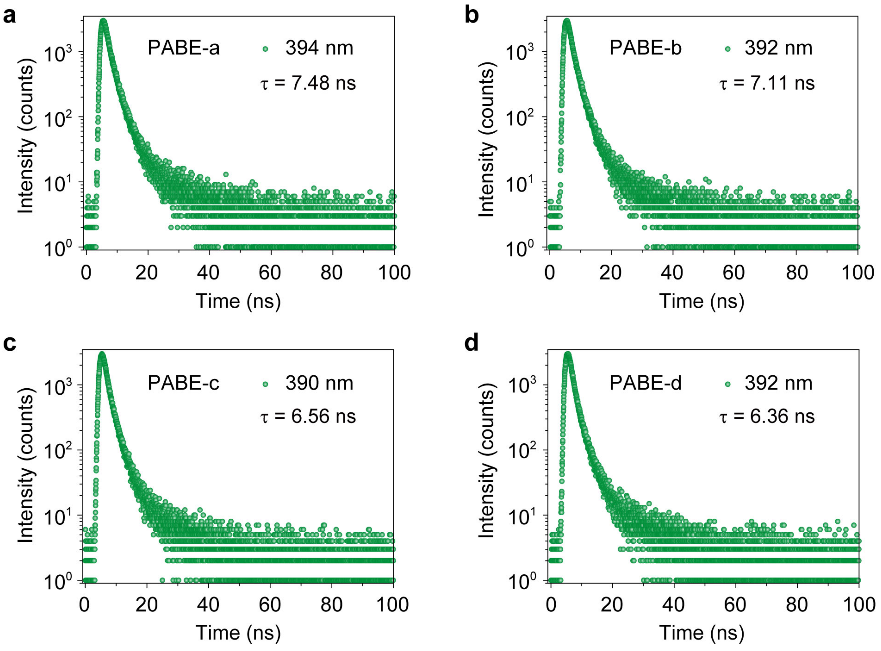

Supplementary Figure 52. Lifetime profiles of the emission bands at around 390 nm for PABE-a to PABE-d films, respectively.

Supplementary Table 8. Luminescence lifetimes (τ) of various PABEs films under ambient conditions.[a]

Polymer	Wavelength (nm)	Fluorescence				Phosphorescence	
		τ_1 (ns)	A_1 (%)	τ_2 (ns)	A_2 (%)	τ_1 (ms)	A_1 (%)
PABE-a	394	1.89	85.23	7.48	14.77		
	548					230.08	100
PABE-b	392	1.81	85.69	7.11	14.31		
	548					221.84	100
PABE-c	390	1.72	85.97	6.56	14.03		
	548					219.64	100
PABE-d	392	1.60	82.53	6.36	17.47		
	548					220.85	100

[a] Determined from the fitting function of $I(t) = A_1e^{-t/\tau 1} + A_2e^{-t/\tau 2}$ according to the luminescence decay curves.

Supplementary Table 9. Photoluminescence efficiencies of PABE-a to PABE-d under ambient conditions.

Polymer	$\lambda_{Fluor.}$ [nm]	$\Phi_{Fluor.}$ [%]	$\lambda_{Phos.}$ [nm]	$\Phi_{Phos.}$ [%]
PABE-a	394	19.90	548	2.10
PABE-b	392	15.97	548	1.23
PABE-c	390	20.50	548	1.20
PABE-d	392	15.73	548	0.97

We further conducted stress-strain curves of copolymer PABE-d at faster tensile rates. As shown in Supplementary Figure 53, as the tensile rate increased from 10 to 80 mm min^{-1}, the strength at break increased and the strain at break decreased. When the tensile rate was below 50 mm min^{-1}, the resulting copolymer could maintain the elongation of beyond 600%. While when the tensile rate further increased to 80 mm min^{-1}, the elongation dropped significantly to 423.6%.

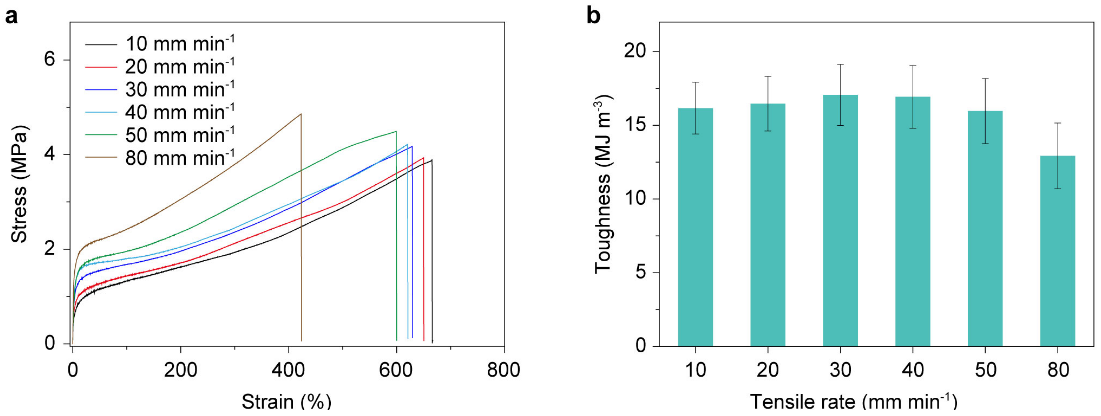

Supplementary Figure 53. (a) Stress-strain curves and (b) Toughness variation of PABE-d film at different tensile rates. Error bars represent mean ± standard deviation (n=3).

Supplementary Table 10. Summary of mechanical properties of PABE-d film at different tensile rates.

Tensile rate (mm min^{-1})	Young's modulus (MPa)	Strain-at-break (%)	Strength-at-break (MPa)	Toughness (MJ m^{-3})
10	13.92	665.1	3.87	16.17
20	17.27	650.9	3.92	16.47
30	22.06	629.3	4.18	17.06
40	25.11	620.5	4.21	16.95
50	28.68	600.2	4.49	15.97
80	33.64	423.6	4.85	12.92

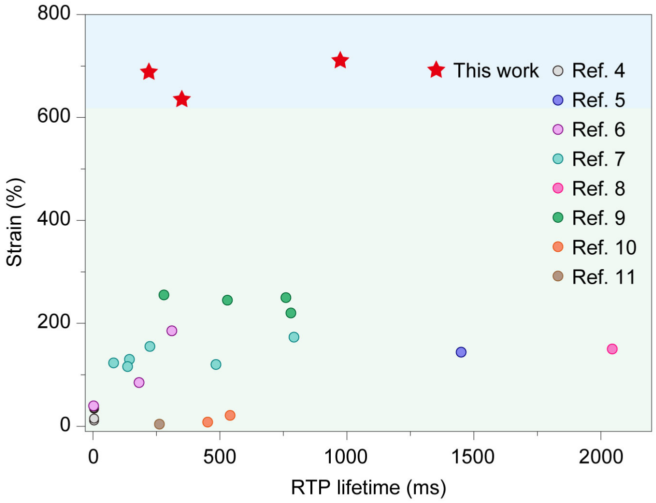

Supplementary Figure 54. Performance comparison between this work and reported works[4-11]. The elongations for most reported RTP polymer films are below 300%, while the stretchability of this work can reach up to 700%, maintaining an ultralong RTP lifetime of up to 981 ms.

Supplementary Table 11. Summary of mechanical properties of PABE-a to PABE-d films.

Sample	Young's modulus (MPa)	Strain-at-break (%)	Strength-at-break (MPa)	Toughness (MJ m^{-3})
PABE-a	73.4	350.4	11.4	33.8
PABE-b	42.6	517.8	9.2	32.1
PABE-c	25.4	606.8	6.0	25.6
PABE-d	15.4	689.5	3.4	17.4

Taking PABE-d as an example, we studied the recovery and residual strain properties by cyclic stress-strain tests (Supplementary Figure 55). The curves showed a pronounced hysteresis, even for a lower applied strain (10%). After the mechanical stress was removed, only partial mechanical properties of the film could be restored. With increasing the mechanical strain, the residual strain after unloading was larger. The considerable residual strain indicated that the polymer film was not able to recover to its initial state within a short time. The rigid molecular structure of the polymer film was possibly affected by mechanical stretching, causing increased non-radiative transition[12]. As a result, the phosphorescence lifetime decreased from 220.85 to 201.36 ms (Supplementary Figure 56). However, this lifetime decrease is only slight, and the polymer film is still able to maintain ultralong RTP after stretching, suggesting good optical stability of the copolymer.

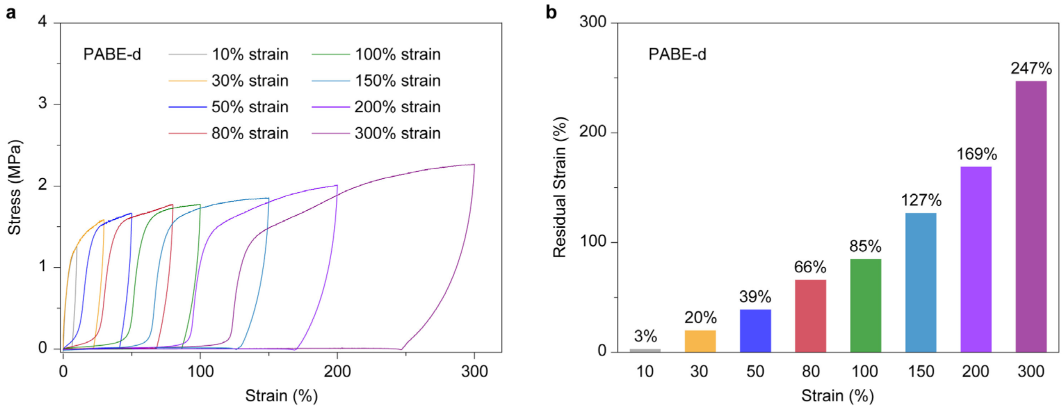

Supplementary Figure 55. (a) Stress-strain curves of PABE-d film in successive stretching to different strains under cyclic loading (loading rate: 10 mm min^{-1}). (b) Residual strains after unloading in different cycles.

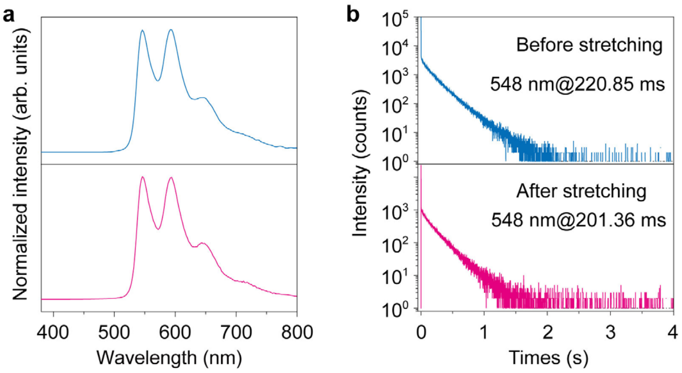

Supplementary Figure 56. (a) Phosphorescence spectra and (b) Lifetime profiles of the emission bands at 548 nm for polymer PABE-d film before and after stretching, respectively.

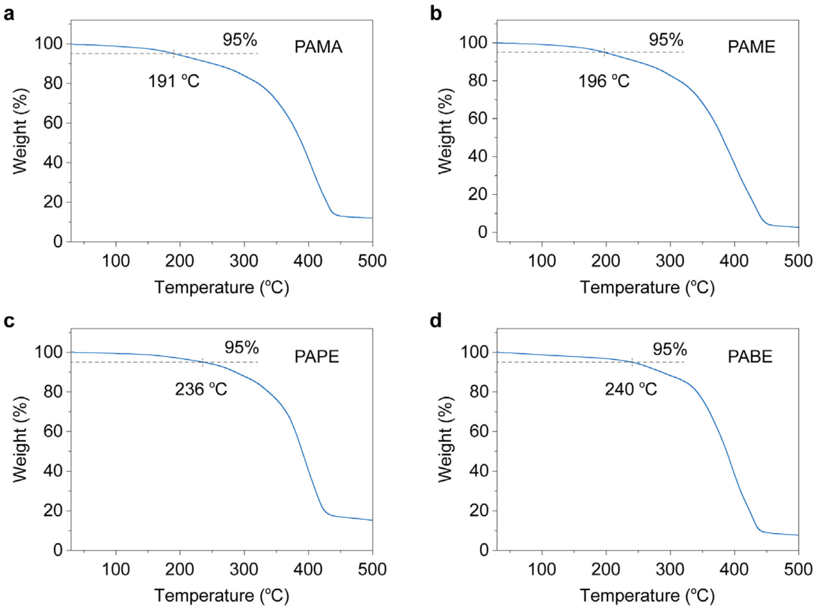

Supplementary Figure 57. TGA curves of PAMA, PAME, PAPE, and PABE films, respectively.

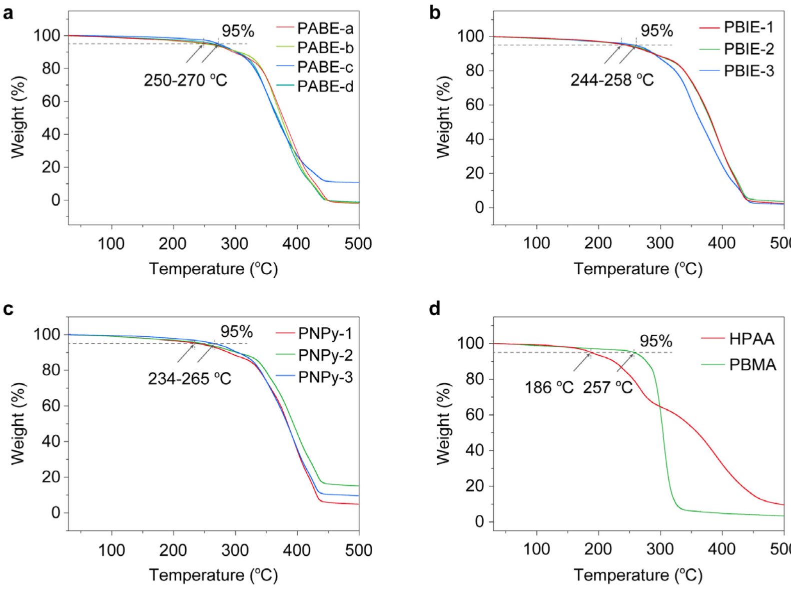

Supplementary Figure 58. TGA curves of copolymers PABEs, PBIEs, PNPys, and control polymers HPAA and PBMA, respectively. The introduction of a soft PBMA segment can improve the thermal performance of copolymer films, which are thermally stable over 230 °C exceeding HPAA film.

The soft polymer PBMA is also a hydrophobic segment, which can further improve the water resistance of PABEs copolymers with a maximum water contact angle of up to 92.6° (Supplementary Figure 59). RTP performance of PABE-d film in water solution was further investigated (Supplementary Figure 60). Although the phosphorescence intensity and lifetime of PABE-d film decreased gradually, it could retain ultralong phosphorescence with a lifetime of 171.0 ms even after being immersed in water for 2 h. This benefitted from the hydrophobic PBMA segment, which could resist and delay the entry of water molecules into the PAA segment and thus protected phosphors for improving the water resistance of block copolymers. Impressively, the RTP lifetime of the soaked film can be recovered to 220.6 ms after being heated under a vacuum at 70 °C for 2 h. After heating, the immersed water molecules were removed from the polymer matrix, and the rigid hydrogen-bond networks between polymer chains and chromophores were restored. Thus, the ultralong RTP lifetime of PABE-d can be recovered to the initial state.

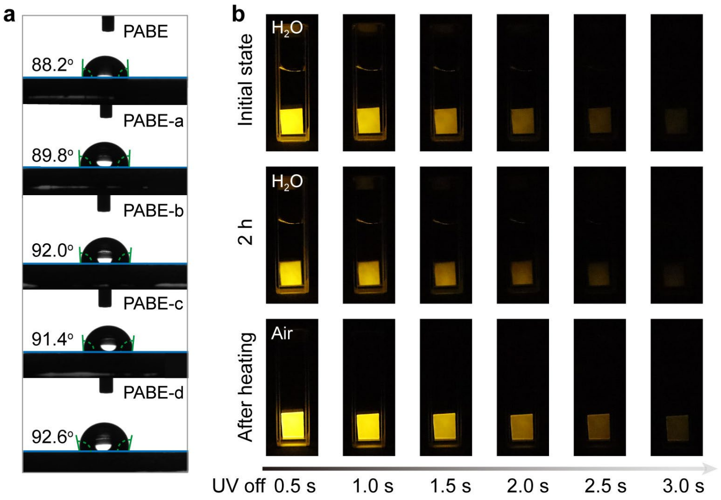

Supplementary Figure 59. (a) The water contact angles of PABEs films. (b) Ultralong phosphorescence photographs of PABE-d film at the initial state, after soaking in water for 2 h, and after heating, respectively.

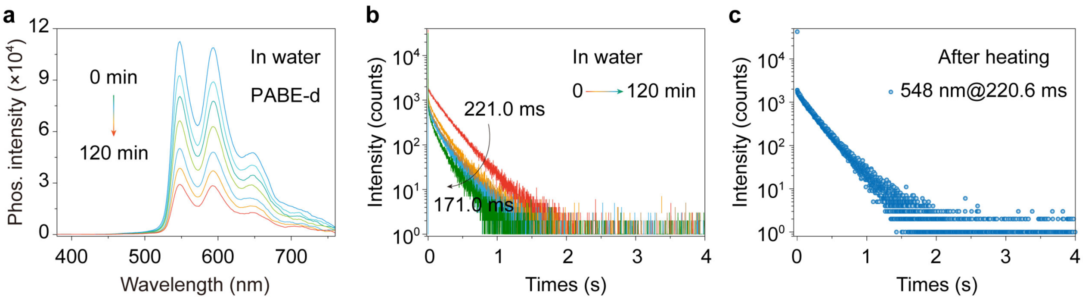

Supplementary Figure 60. (a) Phosphorescence spectra and (b) lifetime profiles of the emission band at 548 nm of PABE-d film after being soaked in water with different times ranging from 0 to 120 min. (c) Lifetime profile of the soaked film after being heated under vacuum at 70 °C for 2 h.

2.2 Mechanism investigation of stretchable phosphorescent polymers

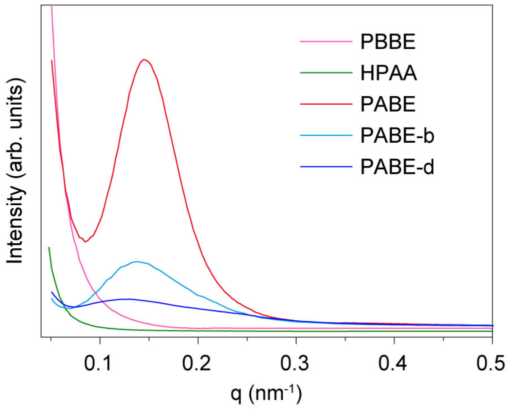

Supplementary Figure 61. SAXS profile plots (not normalized) of PABEs films and control polymers PBBE (unhydrolyzed) and HPAA (*q* represents the scattering factor). The interdomain distance (*d*) can be assessed by SAXS results ($d = 2\pi/q$, where *q* is the position of the SAXS scattering peak). Polymers PBBE and HPAA showed no obvious scattering peak, suggesting the absence of microphase separation in these films.

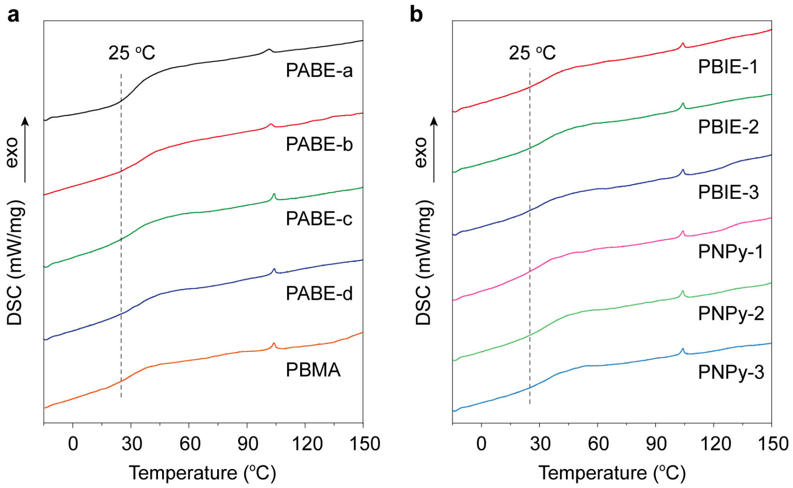

Supplementary Figure 62. DSC curves of (a) PABE-a to PABE-d and control polymer PBMA and (b) PBIE-1 to PBIE-3 and PNPy-1 to PNPy-3 films, respectively.

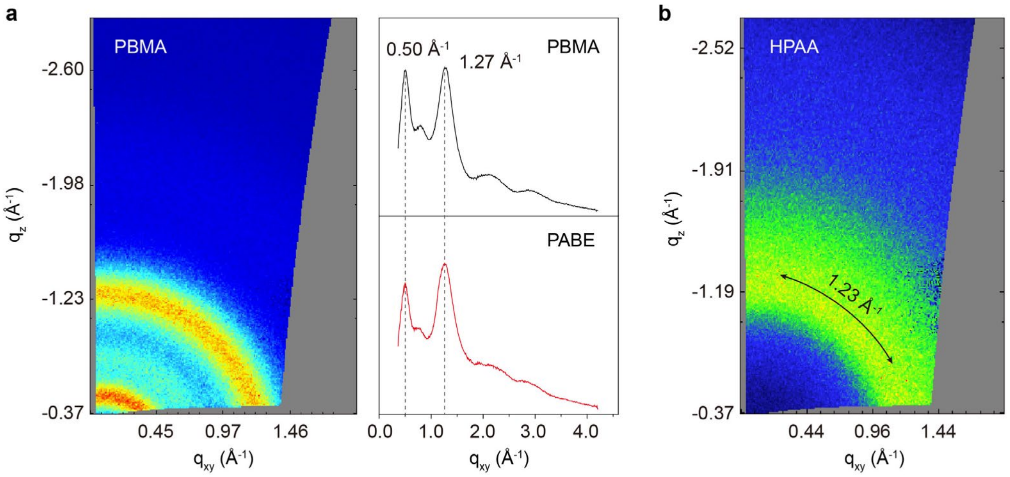

Supplementary Figure 63. Wide angle X-ray scattering patterns of control polymers (a) PBMA and (b) HPAA.

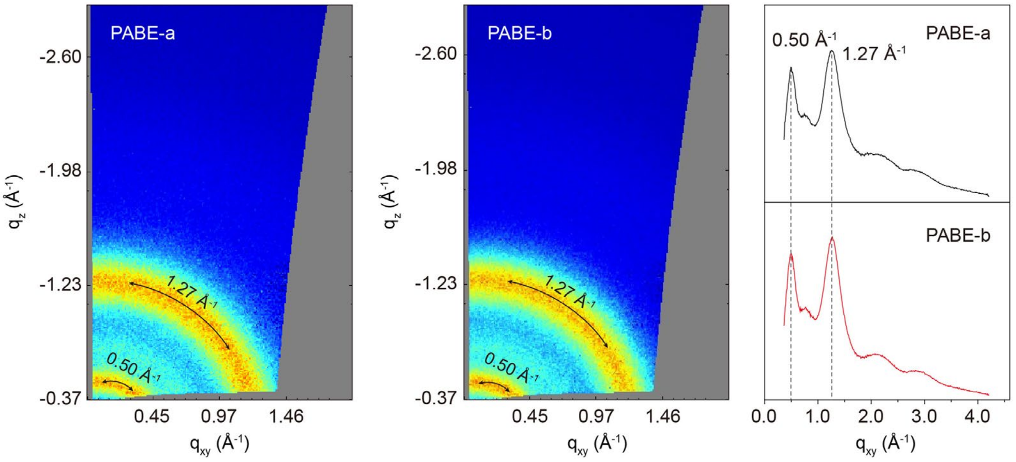

Supplementary Figure 64. Wide angle X-ray scattering patterns of amphiphilic block copolymers PABE-a and PABE-b.

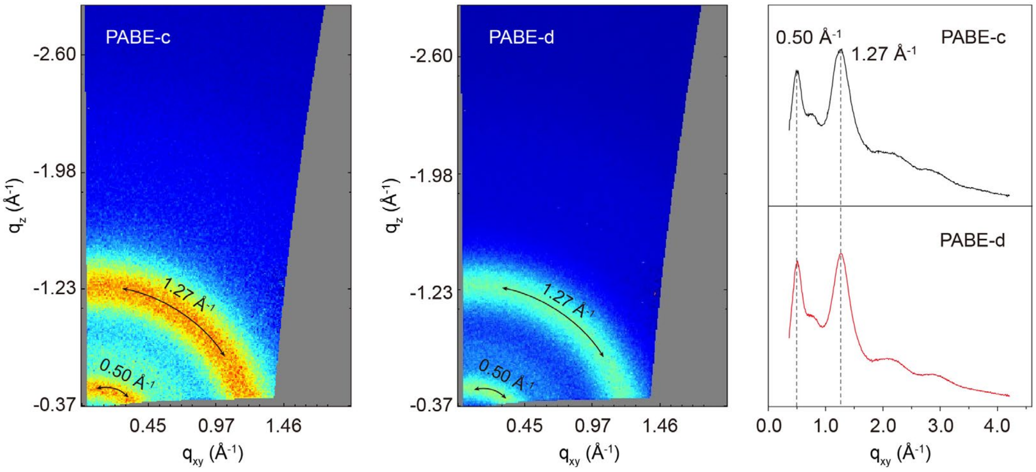

Supplementary Figure 65. Wide angle X-ray scattering patterns of amphiphilic block copolymers PABE-c and PABE-d.

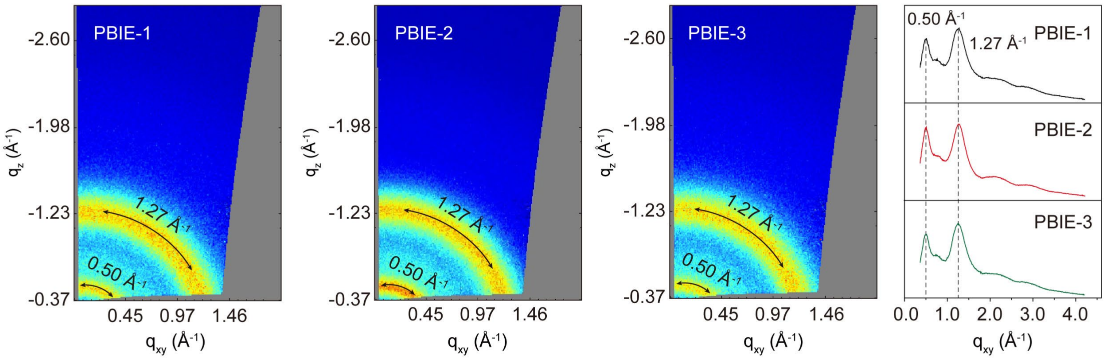

Supplementary Figure 66. Wide angle X-ray scattering patterns of PBIE-1 to PBIE-3 films.

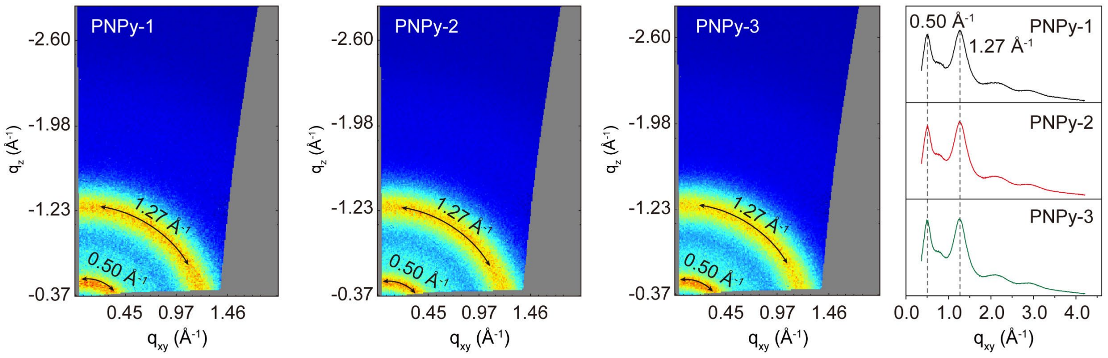

Supplementary Figure 67. Wide angle X-ray scattering patterns of PNPy-1 to PNPy-3 films.

To get deep insights into the origin of afterglow, we prepared a series of hybrid polymer films by doping the phosphor-based initiators into a polyacrylic acid (PAA) matrix with a doped concentration of 0.5%. The photophysical properties of these films were investigated by the photoluminescence spectra and lifetime decay profiles. As depicted in Supplementary Figure 68, the hybrid systems showed RTP with peaks at 416, 479, 512, 548, and 602 nm for MBe-PAA, MBi-PAA, PNa-PAA, DBI-PAA, and MPy-PAA, respectively. Moreover, the phosphorescence bands all exhibited long lifetimes ranging from 219.32 to 1172.56 ms (Supplementary Figure 69). These results are well consistent with the corresponding block copolymers. We also collected the phosphorescence spectra of these initiators in a dilute 2-methyl-tetrahydrofuran solution at 77 K (Supplementary Figure 70). The phosphorescence spectra of these initiators are consistent with block copolymers and PAA-doped hybrid systems. These data demonstrated that the phosphorescence of copolymers originated from the isolate molecule phosphorescence of initiator chromophores in the PAA matrix.

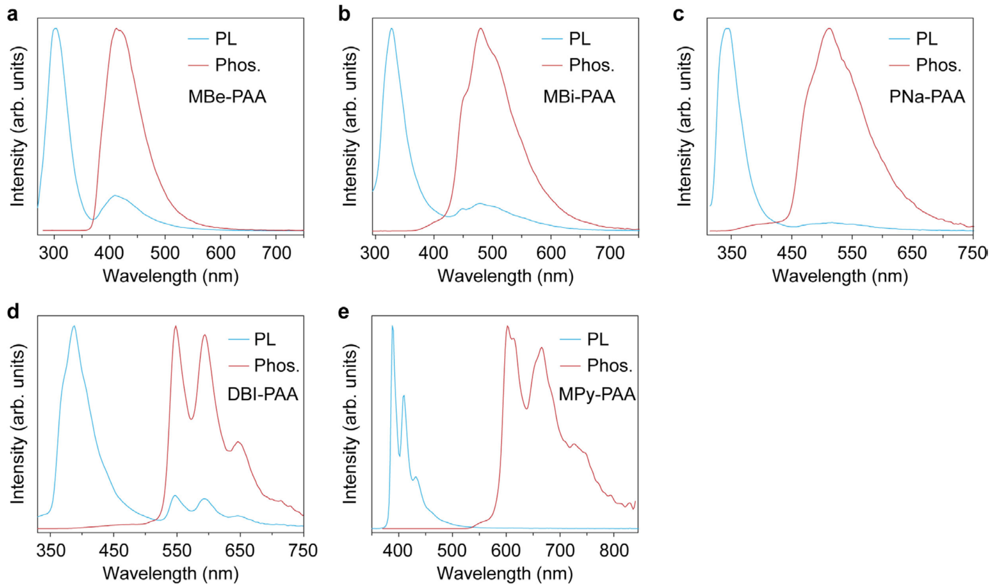

Supplementary Figure 68. Photoluminescence (blue lines) and phosphorescence (red lines) spectra of different initiator-PAA doping systems under ambient conditions.

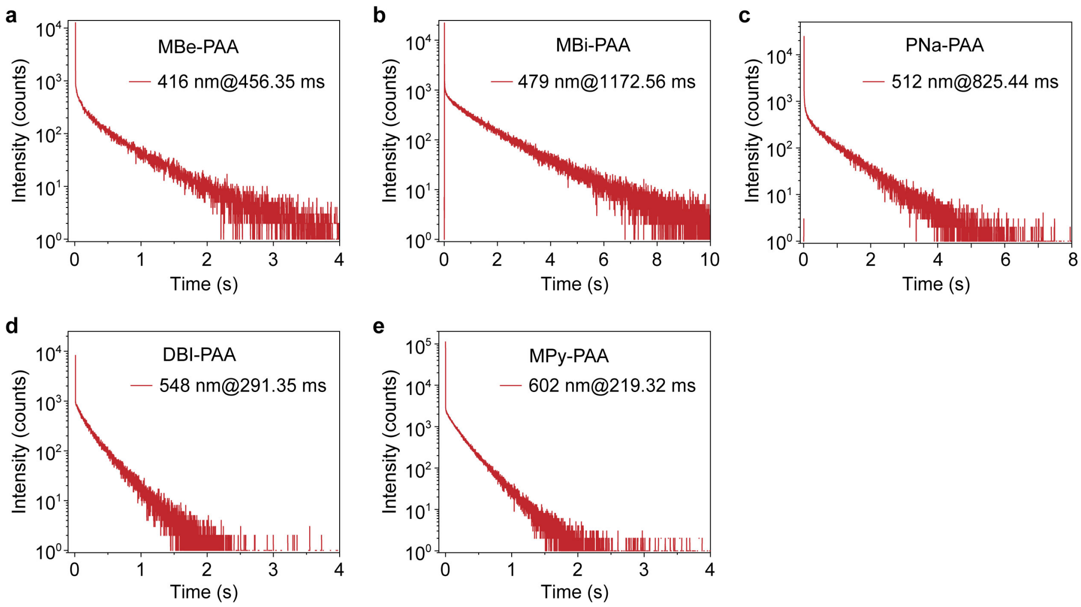

Supplementary Figure 69. Phosphorescence decay profiles of different hybrid systems (a) MBe-PAA, (b) MBi-PAA, (c) PNa-PAA, (d) DBI-PAA, and (e) MPy-PAA under ambient conditions.

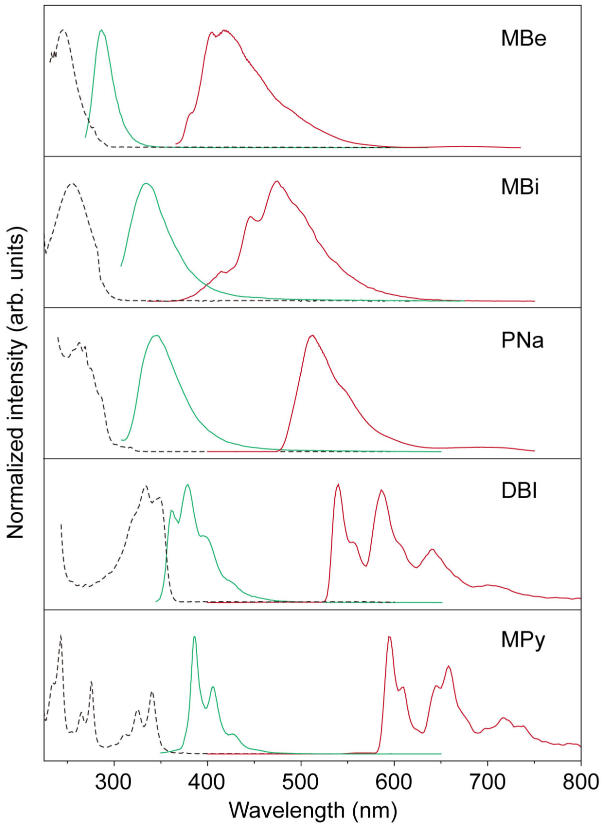

Supplementary Figure 70. UV-vis absorption (black lines) and photoluminescence (green lines) spectra of initiators in dilute DCM solution (10^{-5} M) under ambient conditions, and phosphorescence (red lines) spectra of initiators in dilute 2-methyltetrahydrofuran (10^{-5} M) at 77 K.

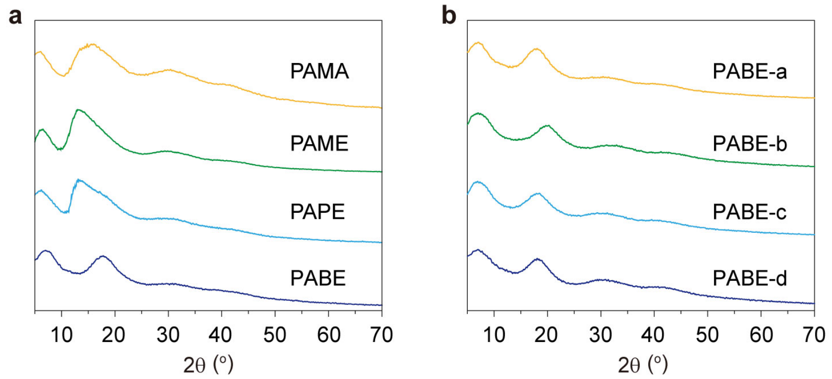

Supplementary Figure 71. XRD patterns of PAMA, PAME, PAPE, and PABEs films. There was no fine scattering peak, demonstrating the amorphous nature of these copolymer films.

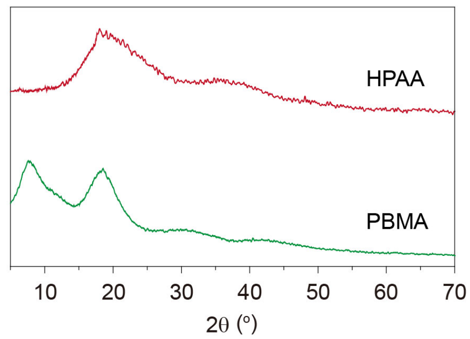

Supplementary Figure 72. XRD patterns of control polymers HPAA and PBMA.

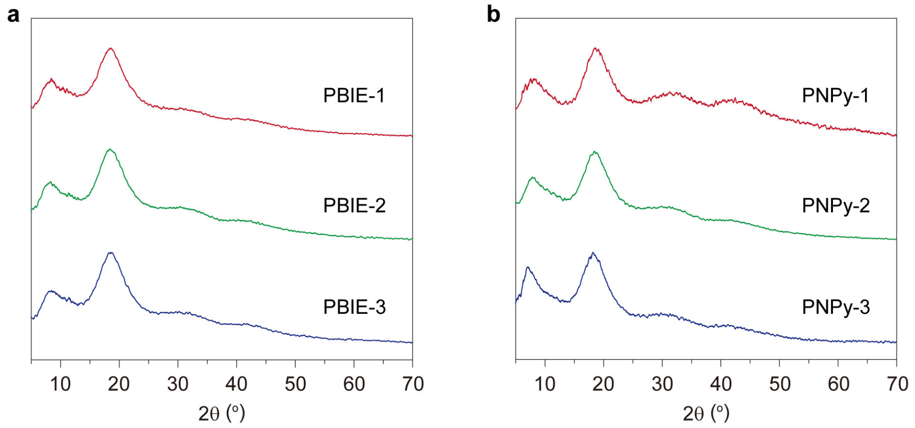

Supplementary Figure 73. XRD patterns of PBIE-1 to PBIE-3 and PNPy-1 to PNPy-3 films, respectively.

To demonstrate the function of hydrogen bonds in enhancing RTP emission, we performed a set of control experiments. Before hydrolyzing, almost no RTP phenomenon can be observed from PBM under ambient conditions (Supplementary Figure 74). This result demonstrates that the PBM matrix cannot effectively protect triplet excitons due to the lack of carboxyl groups to form strong hydrogen bonds for suppressing non-radiative decay. In contrast, after hydrolyzing, ultralong phosphorescence can be easily observed from polymer HPAA under ambient conditions (Supplementary Figure 43). Because after hydrolyzing, there formed numerous carboxyl groups that can generate strong hydrogen bonds between polymer chains and phosphors. Such a hydrogen bonding network can provide a rigid microenvironment to confine molecular motions of chromophores

and decrease the quenching from surrounding oxygen, thus stabilizing the triplet excitons and suppressing non-radiative transition of chromophores for generating ultralong RTP (Supplementary Figure 75). Similarly, due to the lack of a PAA hard block, control polymers PBBE-a and PBMA films almost exhibited no RTP under ambient conditions despite the decent stretchability (Supplementary Figures 76-77). These data demonstrated the importance of hard-soft multiphase design strategy for obtaining stretchable RTP materials.

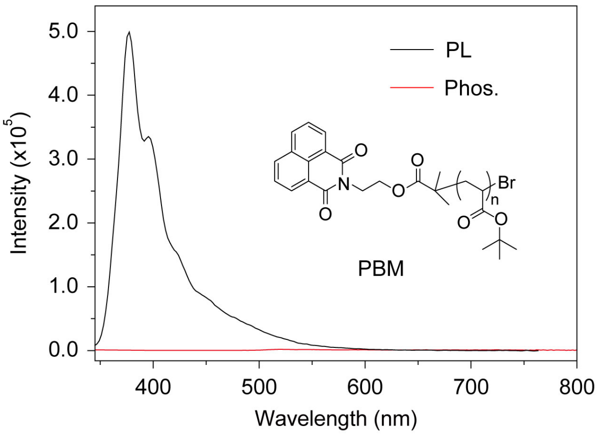

Supplementary Figure 74. Steady-state photoluminescence (black line) and phosphorescence (red line) spectra of polymer PBM under ambient conditions.

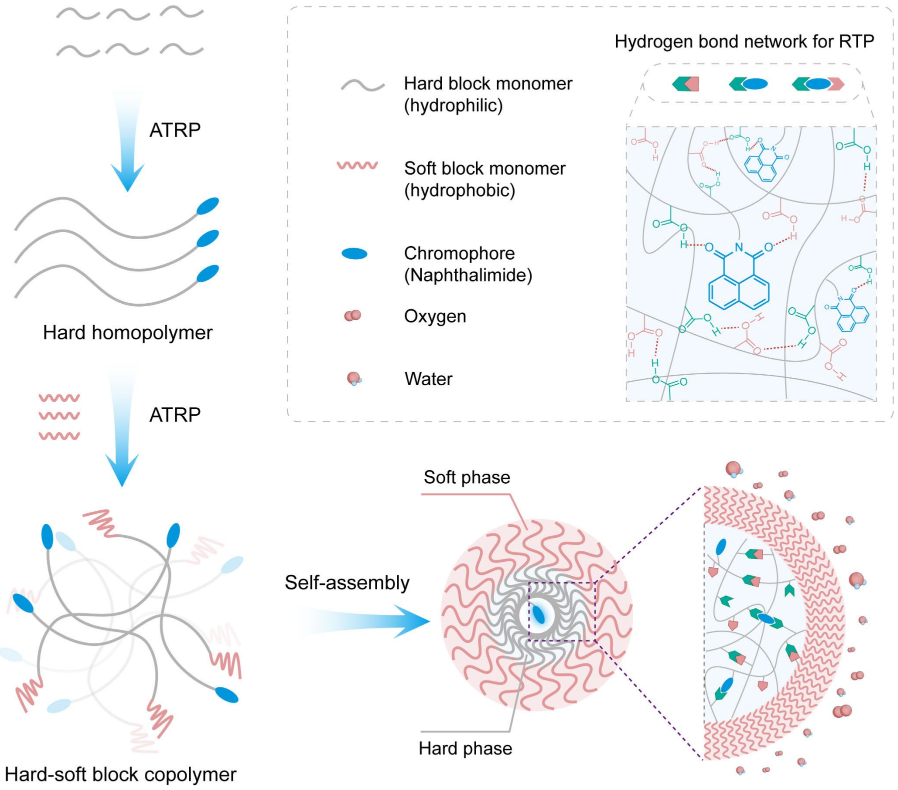

Supplementary Figure 75. Mechanism illustration for the effect of hydrogen bonds on promoting ultralong RTP by multiphase engineering.

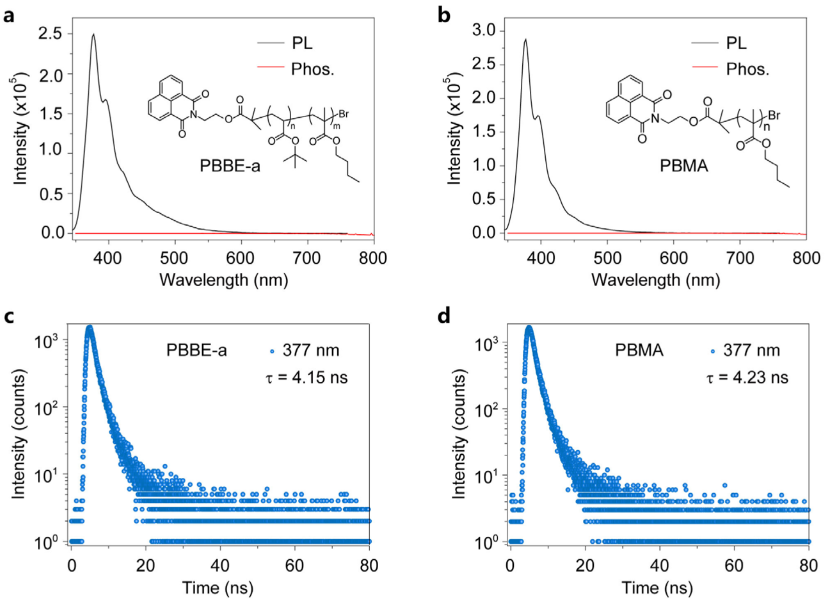

Supplementary Figure 76. Photoluminescence (black lines) and phosphorescence (red lines) spectra of control polymers (a) PBBE-a and (b) PBMA under ambient conditions. Lifetime profiles of the emission band at 377 nm for (c) PBBE-a and (d) PBMA films.

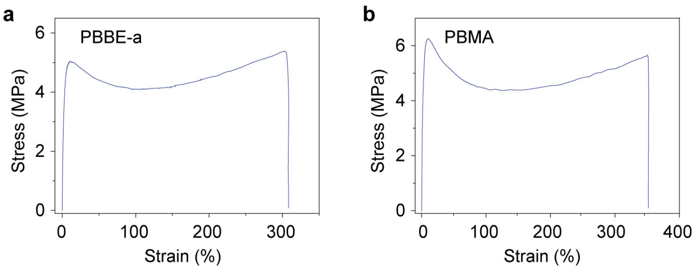

Supplementary Figure 77. Typical stress-strain curves of (a) PBBE-a and (b) PBMA films.

2.3 Theoretical calculations

Calculation details of excited states

Using a Time-Dependent Density Functional Theory (TD-DFT) approach[13], the equilibrium configurations of the T_1 state and the excitation energies, as well as the natural transition orbitals (NTOs) of the low-lying excited states, were performed at the M06-2X/def2-SVP level implemented in Gaussian 09 package[14]. Based on the T_1-optimizted geometries, the spin-orbit coupling (SOC) matrix elements between singlet and triplet excited states were evaluated by Beijing Density Function (BDF) program[15-16].

Details of large-scale coarse-grained molecular dynamics simulations

To further reveal the self-assembly behavior of these block copolymers, we also conducted large-scale coarse-grained molecular dynamics simulations. The model, force field, and simulation details are as follows:

Firstly, the ratio of the DP (degree of polymerization) in the hard and soft blocks is an essential basis for modeling. At the same time, it can be seen that the DBI unit is only a tiny part of the entire polymer chain, which has little effect on the final self-assembly morphology, so the DBI unit is ignored in the simulation model. Based on the above considerations, PABE, PABE-b, and PABE-d in experimental systems are mapped as A6-b-B8, A6-b-B29, and A6-b-B49, respectively.

Secondly, the particle size difference of the two coarse-grained monomers of A (acrylic acid) and B (butyl methacrylate) also needs to be considered. Based on their molar mass (*M*) and density (*ρ*) (Supplementary Table 12), the volume ratios of the two monomers are calculated to be:

$$V_{\mathrm{A}} : V_{\mathrm{B}} = \frac{m_{\mathrm{A}}}{\rho_{A}} : \frac{m_{\mathrm{B}}}{\rho_{\mathrm{B}}} = \frac{72.06}{1.44} : \frac{142.2}{1.06} = 1 : 2.681 \quad \text{(Equation a)}$$

so $D_{\mathrm{A}} : D_{\mathrm{B}} \approx 1.39$.

For such block copolymer chains, the most commonly used bead-spring model has been selected. In this model, a Kuhn unit is coarse-grained as an elementary particle. The neighboring coarse-grained particles on the same chain are constrained by the harmonic bond potential, that is $V_{\mathrm{bond}}(r) = \frac{1}{2}k(r - r_0)^2$, wherein *k* is the spring constant and r_0 is the equilibrium length. It should be noted that the spring bond here is the bond connecting the two adjacent coarse-grained particles, which is different from the chemical bond in the experimental system. Non-bonded interactions between the coarse-grained particles are described by shift Lennard-Jones potential:

$$\begin{aligned} V_{\mathrm{SLJ}}(r) &= 4\varepsilon\left[\left(\frac{\sigma}{r-\Delta}\right)^{12} - \alpha\left(\frac{\sigma}{r-\Delta}\right)^{6}\right] \quad for\, r < (r_{\mathrm{c}} + \Delta) \\ &= 0 \quad\quad for\, r \geq (r_{\mathrm{c}} + \Delta) \end{aligned} \quad \text{(Equation b)}$$

where ε, σ, Δ and r_{c} are the interaction strength, bead size, diameter difference and truncated radius, respectively. $\Delta = (d_i + d_j)/2 - \sigma$, d_i and d_j are the diameters of particles i and j. All simulations are performed with reduced units, the energy, bead diameter, and bead mass are set as $\varepsilon_0 = \sigma_0 = m_0 = 1$. Therefore, the time has a unit of $\tau_0 = \sqrt{m\sigma_0^2/\varepsilon_0}$. In our simulation, d_A and d_B are set as $1\,\sigma_0$ and $1.39\,\sigma_0$, respectively. And the r_{c} is fixed at $2.5\,\sigma_0$. For bond potential, k is fixed at $30\ \varepsilon_0/\sigma_0{}^2$, $r_{\mathrm{m}} = 1.5\,\sigma_0$ and $r_0 = 1.0\,\sigma_0$. Interaction strength parameter ε_{AA}, ε_{BB} and ε_{AB} are set as 1.0, 0.81 and 0.72 respectively. This is determined based on the solubility parameter (SP) of the two blocks as well as the simulation experience.

For each block copolymer, 1000 chains were randomly generated in the simulation box with the initial number density of 0.6 σ^{-3}. Next, the cycles occurred three times between the temperatures of $T = 3.0\ \varepsilon_0/k_{\mathrm{B}}$ and $1.0\ \varepsilon_0/k_{\mathrm{B}}$ under NPT condition and the Andersen barostat. Finally, additional simulations with 1.2×10^6 τ were run and 200 frames of conformation were output for analysis of the structure factors. All simulations in this work were performed with the GALAMOST package[17].

Supplementary Table 12. Summary of physical parameters for acrylic acid (simplified as A) and butyl methacrylate (simplified as B) monomers.

Parameters	Acrylic acid (A)	Butyl methacrylate (B)
ρ (g/mL)	1.44	1.06
M (g/mol)	72.06	142.2
SP (MPa$^{1/2}$)	21.3	18

Supplementary Table 13. Calculated volume fraction of various PABEs samples.

Sample	n	m	n:m	A	B	φ_A
PABE	210	293	1:1.4	6	8	0.22
PABE-b		958	1:4.6		29	0.07
PABE-d		1805	1:8.6		49	0.0436

Herein, n and m represent the degree of polymerization for each block calculated by M_n of PBBE, PBBE-b, and PBBE-d, respectively. φ_A is the volume fraction of the PAA segment in a block copolymer. According to the following formula:

$$\varphi_{\mathrm{A}} = \frac{N_{\mathrm{A}}V_{\mathrm{A}}}{N_{\mathrm{A}}V_{\mathrm{A}} + N_{\mathrm{B}}V_{\mathrm{B}}} \quad \text{(Equation c)}$$

we calculated that in equimolar PAA and PBMA blocks, φ_A was 0.22, 0.07, and 0.0436 for PABE, PABE-b, and PABE-d, respectively. According to the theoretically proposed phase diagram of a linear block copolymer of AB type, the calculated volume fractions were in the spherical phase range[18-20], consistent with the TEM results.

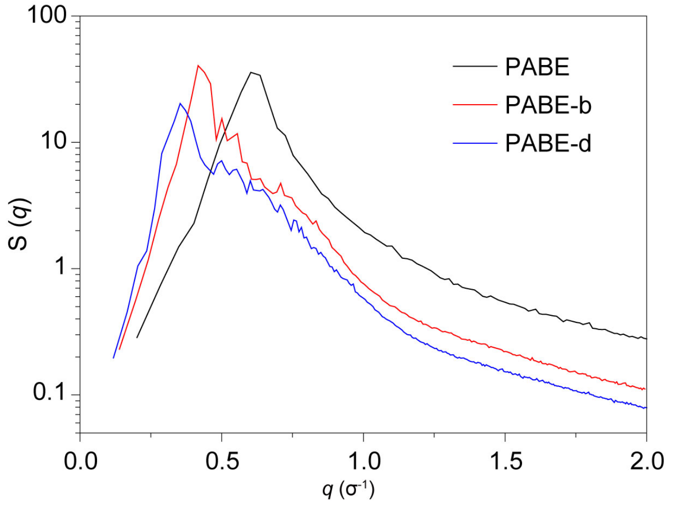

Supplementary Figure 78. Calculated structure factor curves for PABE, PABE-b, and PABE-d, respectively, which match well with SAXS curves of corresponding PABEs samples in experimental systems.

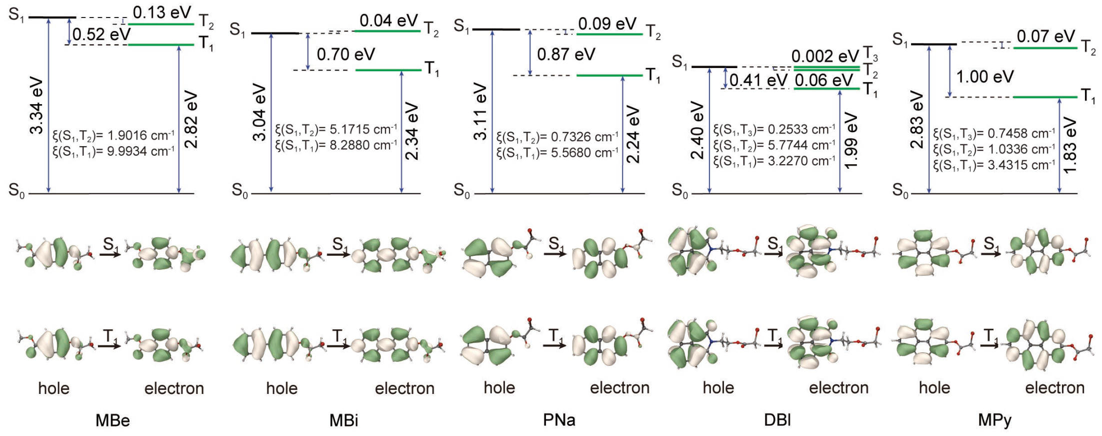

Supplementary Figure 79. Natural transition orbitals (NTOs) for the lowest singlet and triplet states, calculated excitation energies, and SOC constants (ξ) of initiators MBe, MBi, PNa, DBI, and MPy.

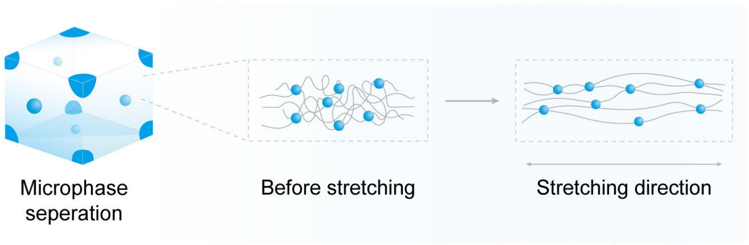

Supplementary Figure 80. Plausible chain arrangement of copolymer films before and during stretching.

2.4 Additional experiments for universality of multiphase engineering

Functional materials with color-tunable and long-lived luminescence have recently gained considerable attention for their potential in multicolor displays. Multiphase engineering also provides a facile platform to design stretchable polymers with color-tunable RTP properties by tailoring the initiator structures and components. To establish the generality of our multiphase design strategy, we expand the binary initiator system PBIEs, which also showed controllable molecular weight, low dispersity, and good uniformity (Supplementary Figure 81). As varying excitation wavelength from 230 to 380 nm, the RTP emission showed a dramatic bathochromic shift from 420 to 548 nm. Notably, under 290 nm excitation, pure white phosphorescence color can be achieved due to the suitable proportion of long-lived blue and yellow phosphorescence mixture (Supplementary Figures 82-84). Besides, the ternary initiator system PNPys can further expand the RTP color tunability ranging from 476 to 600 nm, with stretchability increasing from 278% to 712% (Supplementary Figures 85-88). Interestingly, in addition to the excitation-dependent property, time-dependent afterglow can be observed. This was attributed to the different but comparable phosphorescence intensity and lifetimes between the red and yellow-green emission (Supplementary Figure 89). The color-tunable RTP and good stretchability of these copolymers lay foundations for the next application investigation.

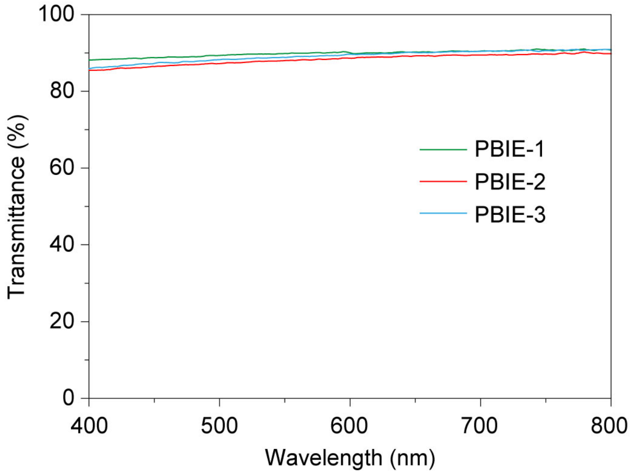

Supplementary Figure 81. Transmittance of PBIE-1, PBIE-2, and PBIE-3 films in the visible range.

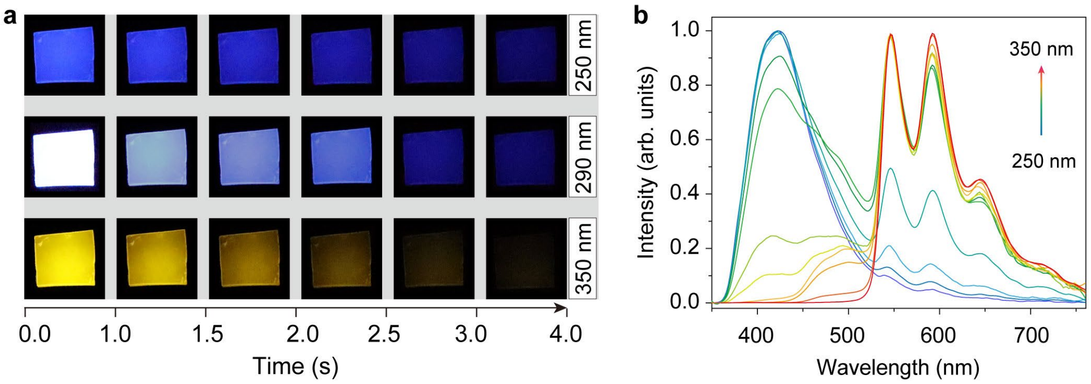

Supplementary Figure 82. (a) Ultralong phosphorescence photographs of PBIE-1 film at different excitation wavelengths after turning off the UV lamps. (b) Phosphorescence spectra of PBIE-1 film at different excitation wavelengths changing from 250 to 350 nm.

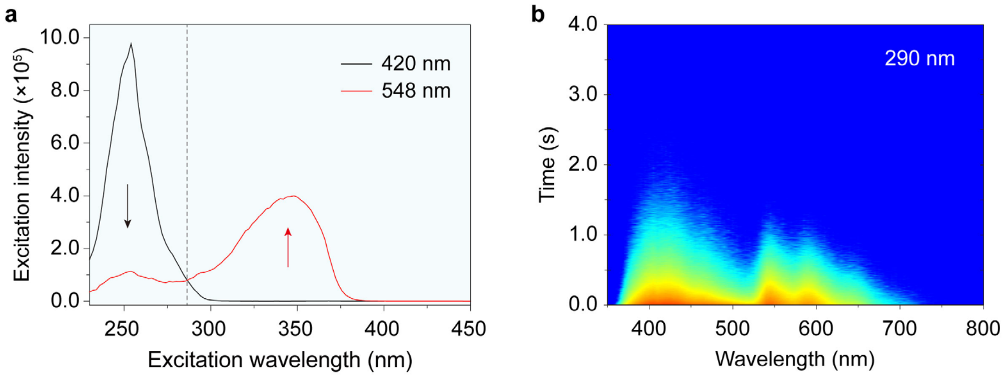

Supplementary Figure 83. (a) Phosphorescence excitation spectra of polymer PBIE-1 at 420 and 548 nm, respectively. (b) Time-resolved emission spectra of PBIE-1 upon 290 nm excitation under ambient conditions.

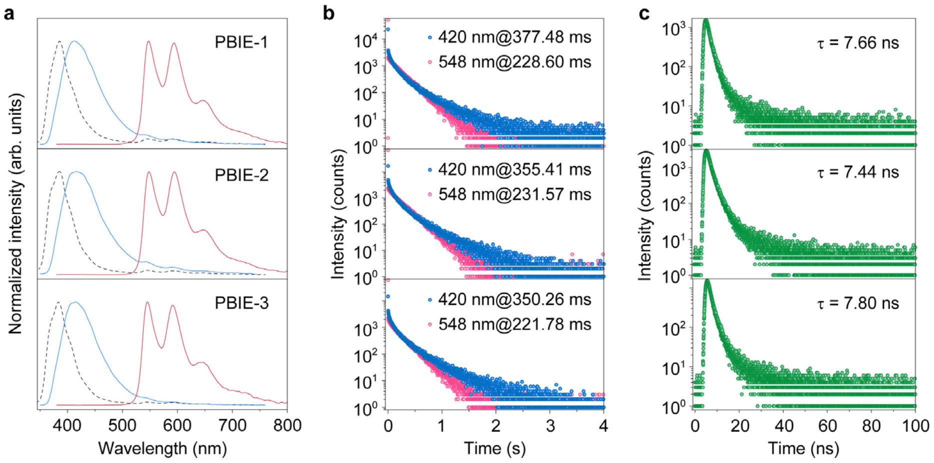

Supplementary Figure 84. Normalized steady-state photoluminescence (dashed lines) and phosphorescence (solid lines) spectra under 250 and 350 nm excitation, and lifetime profiles of emission bands at 420, 548, and 385 nm for PBIE-1 to PBIE-3 films, respectively.

Supplementary Table 14. Luminescence lifetimes (τ) of various PBIE films under ambient conditions.

Polymer	Wavelength (nm)	Fluorescence				Phosphorescence			
		τ_1 (ns)	A_1 (%)	τ_2 (ns)	A_2 (%)	τ_1 (ms)	A_1 (%)	τ_2 (ms)	A_2 (%)
PBIE-1	385	1.71	84.97	7.66	15.03				
	420					100.68	43.4	377.48	56.6
	548					228.60	100		
PBIE-2	385	1.79	83.07	7.44	16.93				
	420					80.57	40.94	355.41	59.06
	548					231.57	100		
PBIE-3	385	1.74	83.89	7.80	16.11				
	420					76.45	40.70	350.26	59.30
	548					221.78	100		

Supplementary Table 15. Summary of mechanical properties of PBIE-1 to PBIE-3 films.

Sample	Young's modulus (MPa)	Strain-at-break (%)	Strength-at-break (MPa)	Toughness (MJ m^{-3})
PBIE-1	107.4	280.5	11.5	27.89
PBIE-2	63.0	537.4	8.4	31.46
PBIE-3	25.4	636.8	4.1	18.1

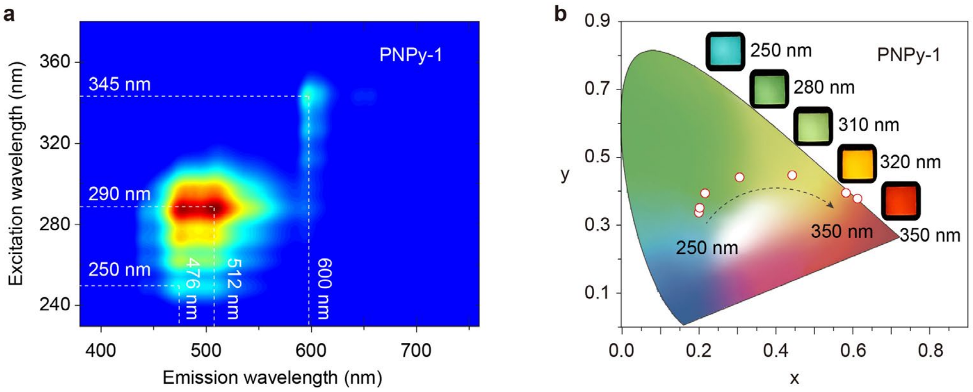

Supplementary Figure 85. (a) Excitation-phosphorescence mapping of copolymer PNPy-1 under ambient conditions. (b) CIE chromaticity diagram of phosphorescence color with excitation varying from 250 to 350 nm. Inset: long-lived luminescence photographs at different excitation wavelengths after turning off the UV lamps.

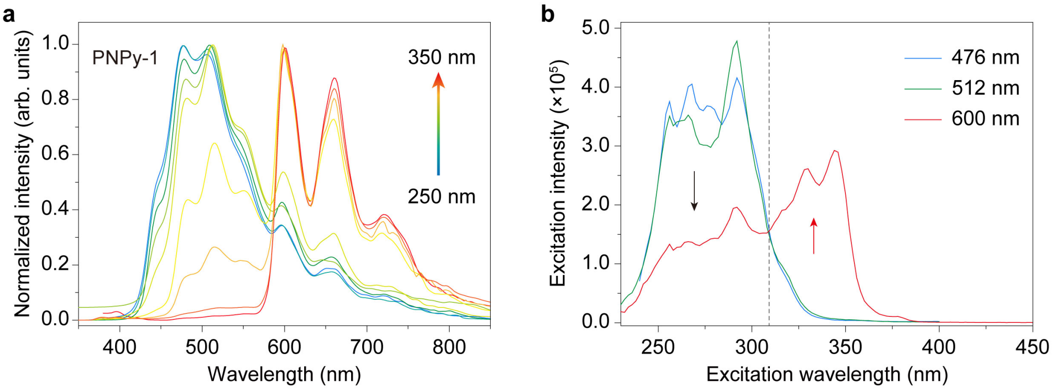

Supplementary Figure 86. (a) Phosphorescence spectra of PNPy-1 film under different excitation wavelengths. (b) Phosphorescence excitation spectra of PNPy-1 at 476, 512, and 600 nm, respectively.

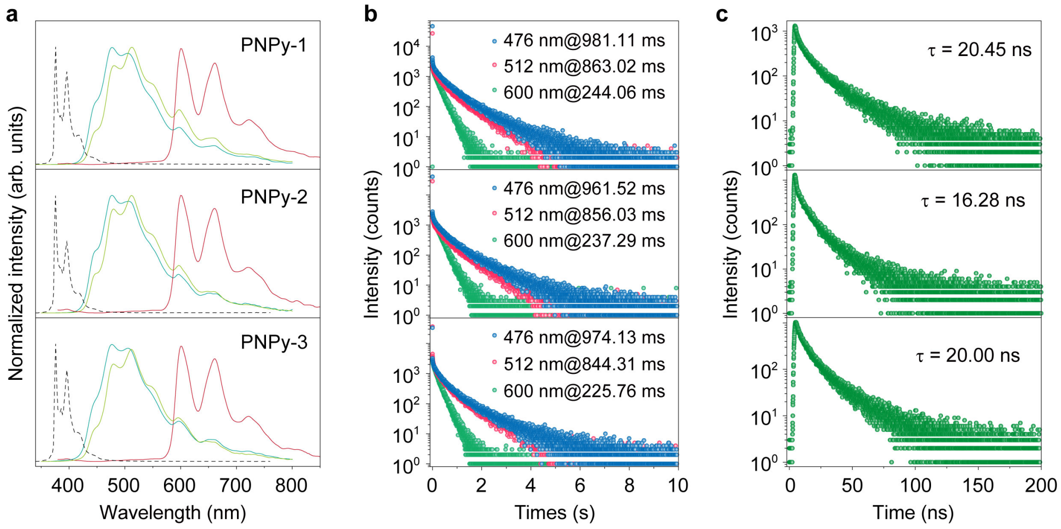

Supplementary Figure 87. Normalized steady-state photoluminescence (dashed lines) and phosphorescence (solid lines) spectra under 250, 290, and 350 nm excitation, and related lifetime profiles of the emission bands at 476, 512, 600, and 376 nm for PNPy-1 to PNPy-3 films, respectively.

Supplementary Table 16. Luminescence lifetimes (τ) of various PNPy films under ambient conditions.[a]

Polymer	Wavelength (nm)	Fluorescence				Phosphorescence			
		τ_1 (ns)	A_1 (%)	τ_2 (ns)	A_2 (%)	τ_1 (ms)	A_1 (%)	τ_2 (ms)	A_2 (%)
PNPy-1	376	4.16	33.2	20.45	66.8				
	476					329.82	40.49	981.11	59.51
	512					260.30	34.71	863.02	65.29
	600					244.06	100		
PNPy-2	376	2.97	31.36	16.28	68.64				
	476					261.70	36.17	961.52	63.83
	512					217.78	30.84	856.03	69.16
	600					237.30	100		
PNPy-3	376	5.41	42.89	20.00	57.11				
	476					251.04	44.81	974.13	55.19
	512					200.62	39.21	844.31	60.79
	600					225.76	100		

[a] Determined from the fitting function of $I(t) = A_1e^{-t/\tau_1} + A_2e^{-t/\tau_2}$ according to the luminescence decay curves.

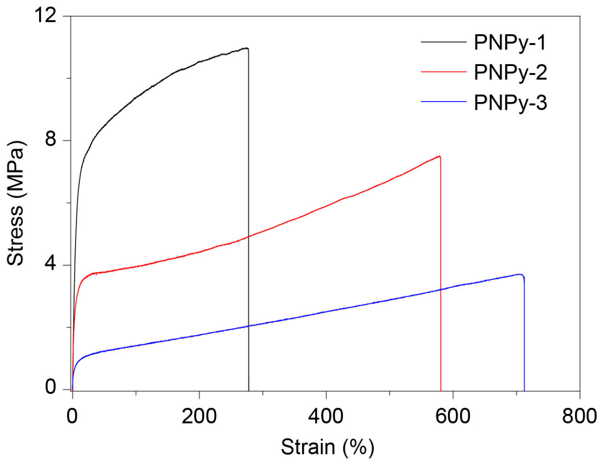

Supplementary Figure 88. Typical stress-strain curves of PNPy-1 to PNPy-3 films.

Supplementary Table 17. Summary of mechanical properties of PNPy-1 to PNPy-3 films.

Sample	Young's modulus (MPa)	Strain-at-break (%)	Strength-at-break (MPa)	Toughness (MJ m^{-3})
PNPy-1	97.8	277.5	10.5	26.4
PNPy-2	54.6	580.5	7.1	30.1
PNPy-3	15.4	712.3	3.4	16.7

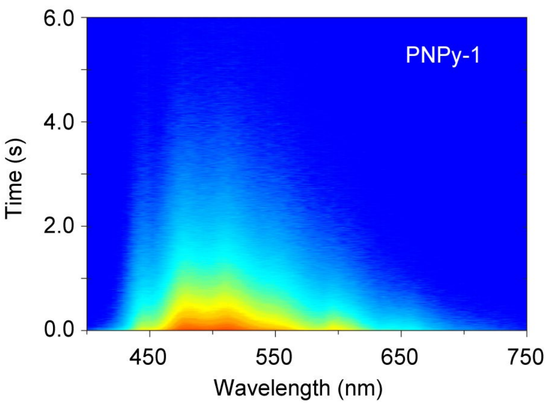

Supplementary Figure 89. Time-resolved emission spectra of copolymer PNPy-1 excited by 300 nm.

The above-discussed block copolymers exhibit long-lived RTP originating from the selected initiators. According to Equation d-f,

$$\Phi_{\mathrm{p}} = \Phi_{\mathrm{isc}} k_{\mathrm{p}} \tau_{\mathrm{p}} \quad \text{(d)}$$

$$\Phi_{\mathrm{isc}} = \frac{k_{\mathrm{isc}}}{k_{\mathrm{f}}+k_{\mathrm{ic}}+k_{\mathrm{isc}}} \quad \text{(e)}$$

$$\tau_{\mathrm{p}} = \frac{1}{k_{\mathrm{p}}+k_{\mathrm{nr}}} \quad \text{(f)}$$

due to the weak spin-orbit coupling (SOC) and rapid nonradiative decay rate in purely organic compounds, it is challenging to achieve phosphors with simultaneous efficiency and lifetime enhancement[21-23]. Therefore, these block copolymers display lower phosphorescence quantum yield. Benefitting from multiphase design strategy, stretchable polymer with efficient RTP can also be achieved through tailoring or replacing the initiators.

To further demonstrate the generality of multiphase engineering, block copolymers PABr and PPBr were synthesized by utilizing Br-modified initiators to improve the phosphorescence quantum yield. As shown in Supplementary Figure 91, after introducing Br atom to the initiators, the phosphorescence proportion in PL spectra showed a dramatic increase up to 91% and 96% for PABr and PPBr films, respectively. Benefitting from the heavy atom effect for enhanced SOC and thus promoted intersystem crossing (ISC) process, the RTP quantum yields of PABr and PPBr films increased to 35.90% and 13.85%, maintaining a good stretchability of 653.8% and 633.1%, respectively (Supplementary Table 18). These data indicated that our proposed multiphase design principle can also prepare stretchable copolymer films with efficient RTP, demonstrating the generality of our strategy.

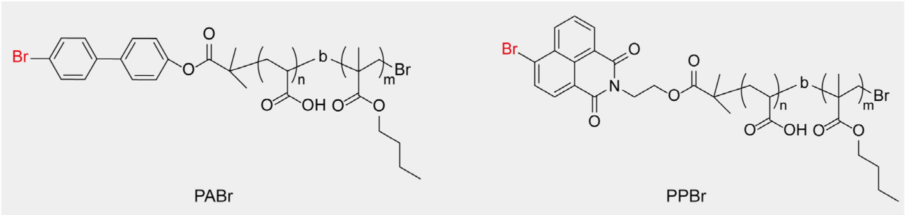

Supplementary Figure 90. Chemical structures of block copolymers PABr and PPBr.

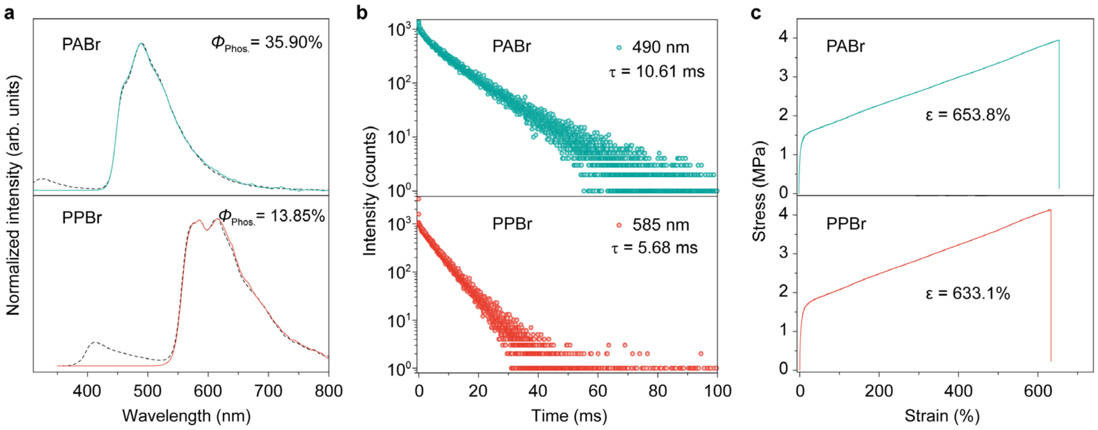

Supplementary Figure 91. (a) Normalized steady-state PL (dotted lines) and phosphorescence (solid lines) spectra for PABr and PPBr under ambient conditions. (b) Lifetime decay curves of emission bands at 490 and 585 nm for PABr and PPBr, respectively. (c) Stress-strain curves of PABr and PPBr at room temperature.

Supplementary Table 18. Photoluminescence efficiencies of PABr and PPBr films under ambient conditions.

Polymers	$\lambda_{\mathrm{Fluor.}}$ (nm)	$\Phi_{\mathrm{Fluor.}}$ (%)	$\lambda_{\mathrm{Phos.}}$ (nm)	$\Phi_{\mathrm{Phos.}}$ (%)
PABr	328	1.50	490	35.90
PPBr	413	1.35	585	13.85

2.5 Applications

Combining the color-tunable RTP and good stretchability of our designed block copolymers, we explored their potential in data encryption, information storage, and flexible multicolor afterglow display. These results will lay foundation for extending the application potential of phosphorescence polymers to new domains.

Multi-level volumetric data encryption

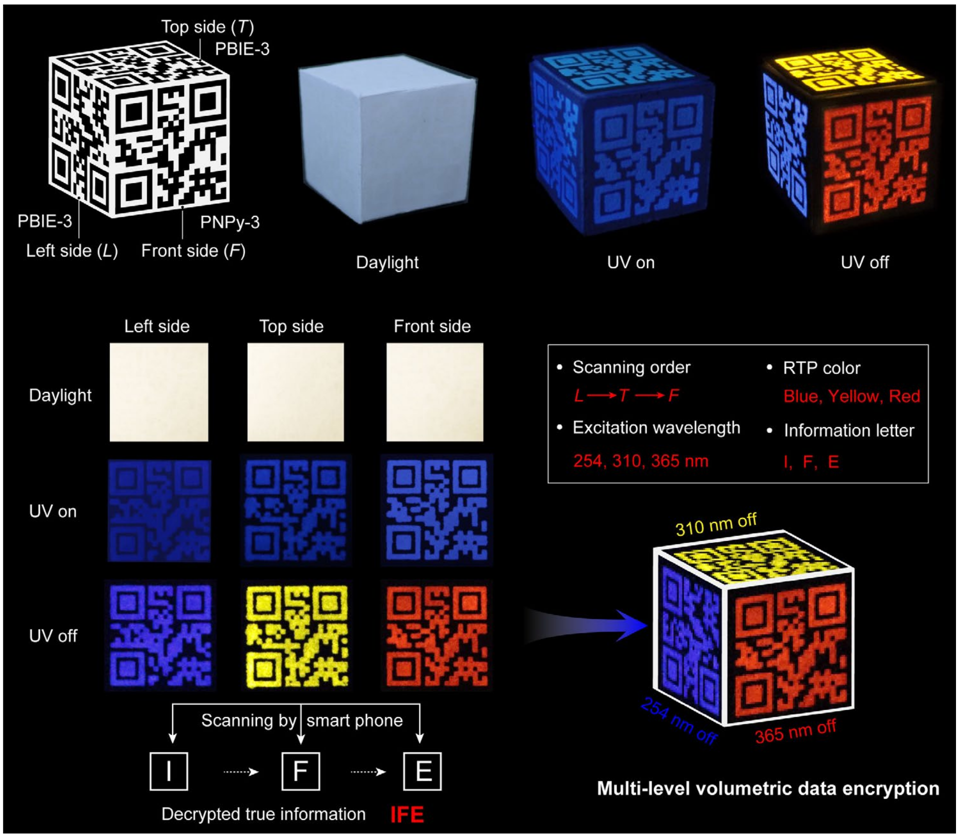

Supplementary Figure 92. Schematic illustration for the potential application of color-tunable flexible RTP copolymers in multi-level volumetric data encryption.

With the rapid development of the information age, more dimensional encryption techniques are urgently needed for data anti-counterfeiting because of the higher security and availability, and larger storage capacity. Based on screen printing, three quick response (QR) codes carrying “I”, “F”, and “E” information were painted on different sides of a cube utilizing PBIE-1 and PNPy-1 samples (Supplementary Figure 92). Under daylight, there is no pattern can be observed on the three sides of the cube by the naked eye. Under the irradiation of UV lamps of 254, 310, and 365 nm, respectively, three blue QR codes can be observed. Due to the similar color, the true information is difficult to recognize. After switching off these UV lamps, three clear QR codes with blue, yellow, and red colors appeared. Three distinct colors spanning the entire visible range help to distinguish the different QR codes by the naked eye in a dark environment conveniently. Only when we obey the right scanning order from blue to yellow and then to red QR codes can we get the decrypted true data “IFE” quickly. Unsuitable excitation wavelengths will make the different QR codes hard to identify quickly in a dark environment, increasing decoding difficulty and even resulting in the wrong data. Meanwhile, the wrong scanning order would lead to the false information “IEF”, “EFI”, “EIF”, etc. Therefore, based on a 3D information carrier, the scanning order, excitation wavelength, and afterglow colors would effectively improve the encryption levels. Utilizing the excitation-dependent RTP features of the designed copolymers, we can realize multi-level volumetric data encryption and identification in darkness.

Information storage

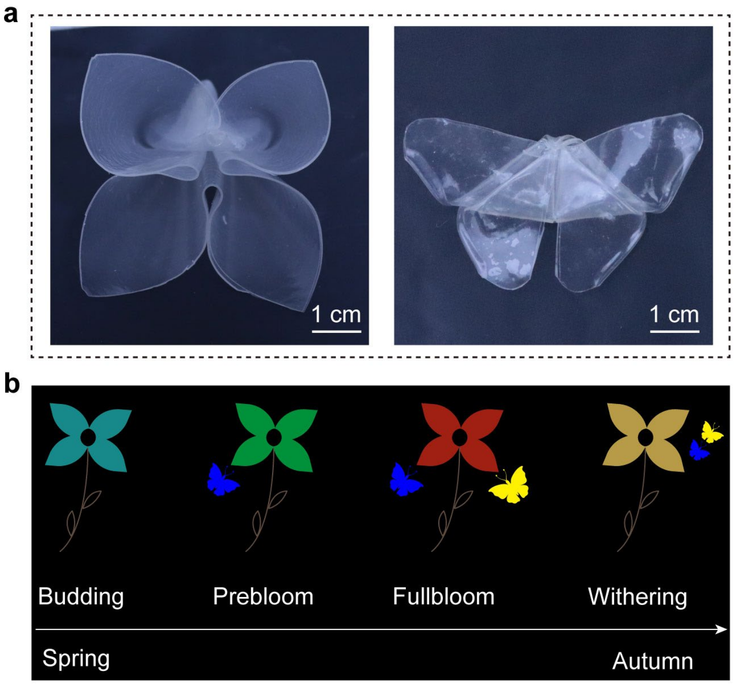

Supplementary Figure 93. (a) Photographs of the hand-folded flower (PNPy-3 film) and butterfly (PBIE-3 film) taken under daylight. (b) Schematic illustration of potential application in information storage combining the excitation- and time-dependent afterglow of different flexible copolymer films.

The flexibility and multicolor RTP of copolymer films endow potential in information storage. As shown in Supplementary Figure 93, the paintings record the color variation of a flower in different seasons, accompanied by the butterflies attracted and then flying away. The blue, green, red, and yellow flowers drive from the multicolor afterglow of PNPy-3 film after stopping 254 nm, 310 nm (delay 1 s), 365 nm, and 310 nm UV light excitation, respectively. The blue and yellow butterflies originate from the afterglow of PBIE-3 film after stopping 254nm and 365 nm excitation, respectively. These paintings demonstrated the potential of multicolor flexible copolymers in instant information recording and reading.

Afterglow display

Beyond the above applications, the afterglow display of flexible RTP copolymers can be realized via combining electrical excitation. The photoelectric devices with various copolymer films were fabricated based on light-emitting diode (LED) arrays via a circuit diagram (Supplementary Figure 94). Meanwhile, the switch of LED arrays can be controlled by the relative computer programs for realizing digit display and conversion, radar detection, and stretchable afterglow display.

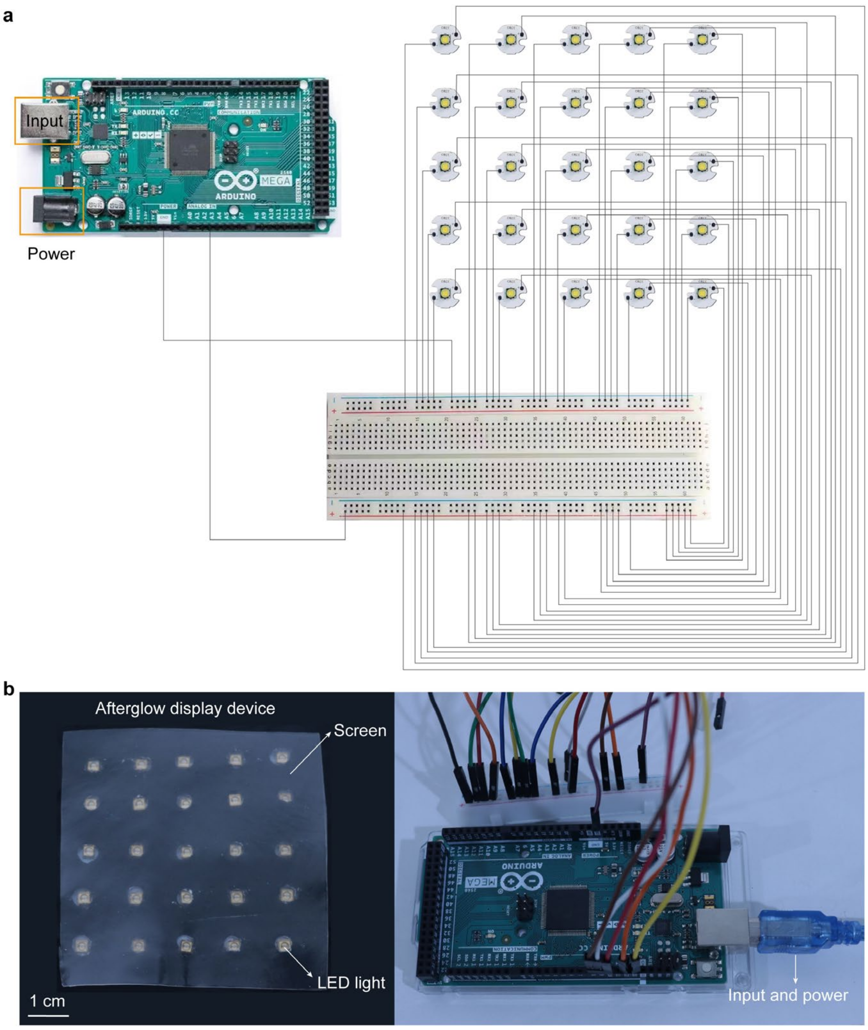

Supplementary Figure 94. Demonstration of afterglow display with electricity. (a) Circuit diagram of the display device for digit and radar detection displays mimicry. (b) Photographs of the afterglow display device taken under daylight.

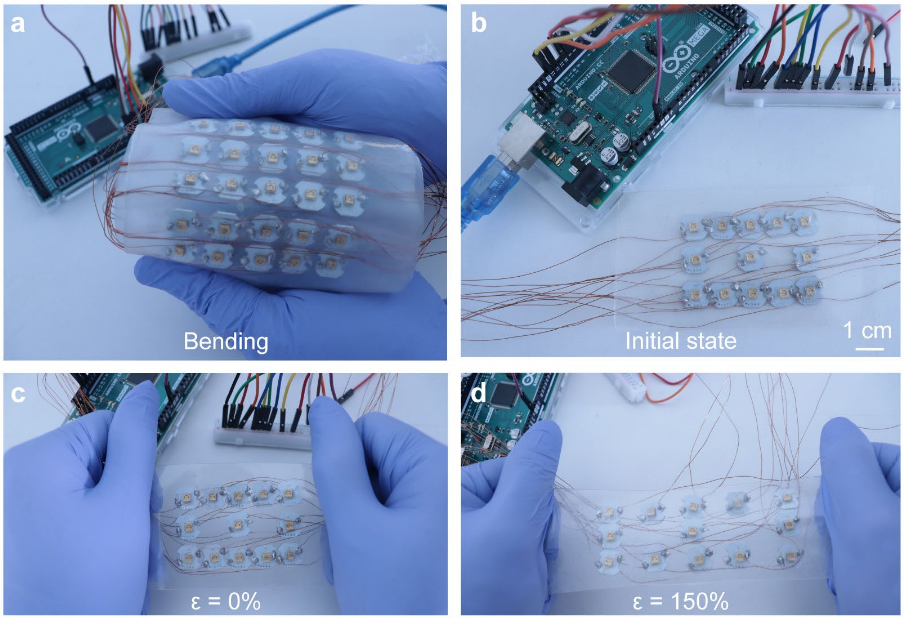

Supplementary Figure 95. Photographs of the afterglow display pixels subjected to (a) bending and (b-c) stretching taken under daylight.

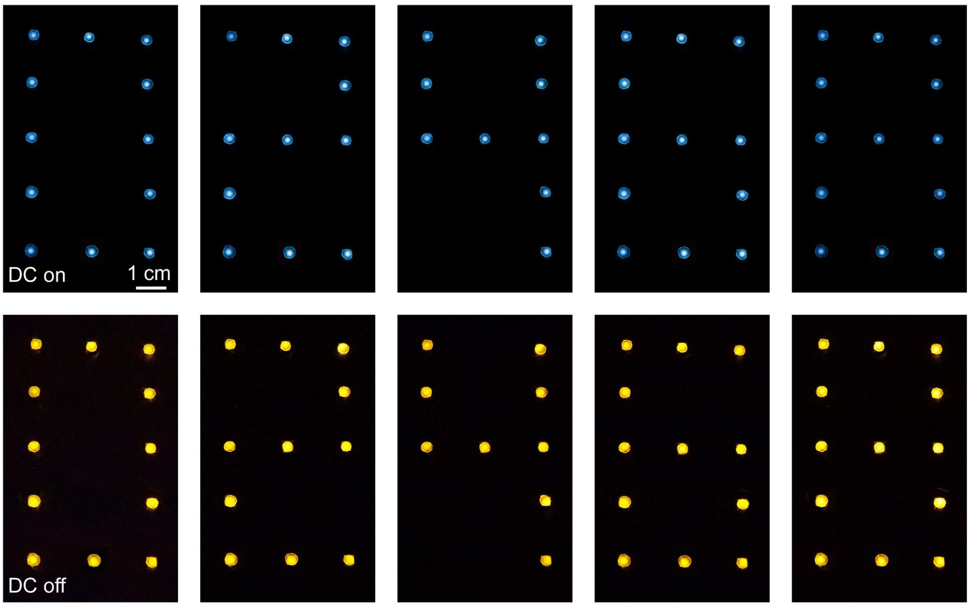

Supplementary Figure 96. Digit display under direct current (DC) switched on and off utilizing PABE-d film as a display screen. Using the afterglow display device, even numbers from 0 to 8 can be displayed and converted by manipulating the DC on and off.

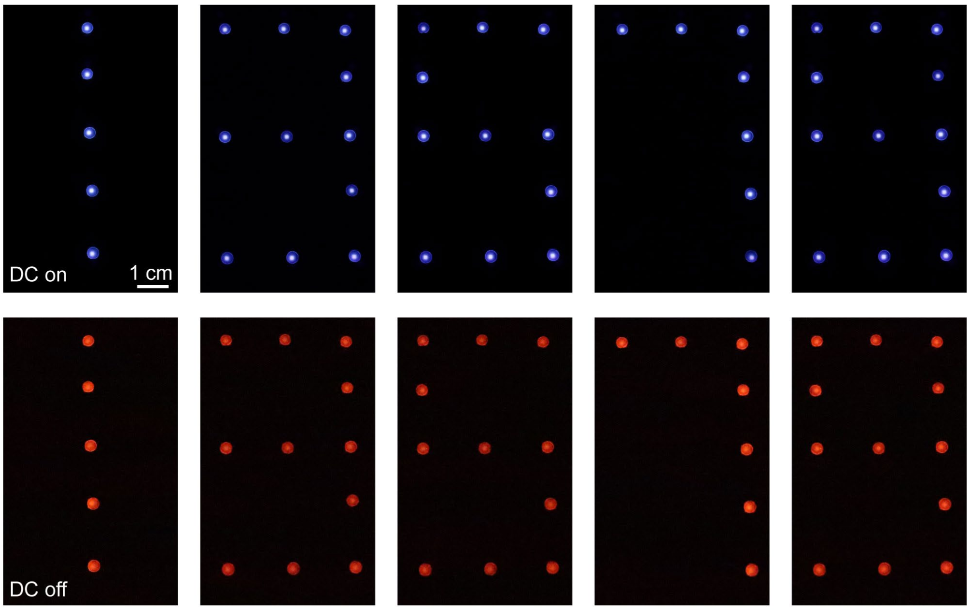

Supplementary Figure 97. Digit display under direct current (DC) switched on and off utilizing PNPy-3 film as a display screen. Using the afterglow display device, odd numbers from 1 to 9 can be displayed and converted by manipulating the DC on and off.

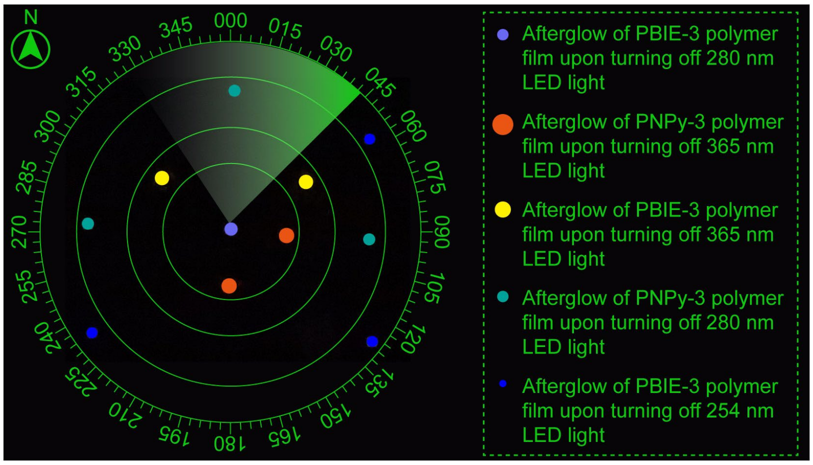

Supplementary Figure 98. Schematic illustration for the potential of color-tunable flexible polymer films in radar detection by controlling the DC off. Blue-white, red, yellow, green, and blue afterglow originated from PBIE-3 or PNPy-3 polymer film upon turning off 280, 365, or 254 nm LED lights controlled by direct current.

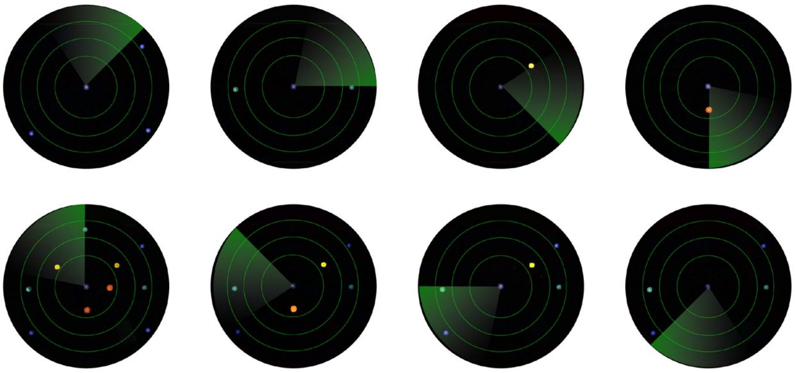

Supplementary Figure 99. Photographs of radar all-round detection of the targets at different distances by controlling the DC off.

Color-tunable flexible RTP polymer films can be potentially utilized as display screens for simulating radar all-round detection (Supplementary Figures 98-99). When the detected targets are in the free zone, a blue afterglow will be displayed. When the detected targets are in the safe zone, a green afterglow will be displayed. Targets in the two zones would not attract much attention. When the detected targets are in the danger zone, a bright yellow afterglow will be displayed, and our forces must maintain a high degree of vigilance. When the targets are closer to the warning zone, a red afterglow will be displayed, and our forces must act swiftly to respond to the emergency. Colorful afterglow in different zones can be realized by controlling the switch of different LED arrays through computer programs.

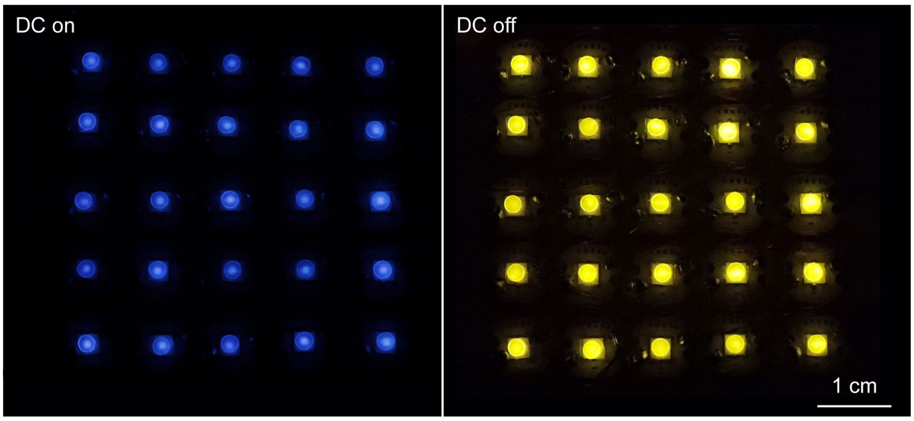

Supplementary Figure 100. Afterglow display array based on PABE-d film under DC switched on and off.

Color-tunable flexible copolymer films can also be potentially utilized for full-color afterglow display by controlling electrical excitation. PBIE-3 and PNPy-3 films were applied as display screens. Under DC switched off, when the LEDs vary from 255 to 370 nm, the display colors can change from blue (0.17, 0.10) to yellow (0.50, 0.50) utilizing PBIE-3 screen, and from blue-green (0.20, 0.34) to red (0.62, 0.63) using PNPy-3 screen. The related CIE chromaticity diagram showed a wide gamut coverage (Supplementary Figure 101 and Supplementary Table 19). Based on one copolymer film, an on-demand information display with various colors can be realized without needing to synthesize complex materials but change excitation sources, which can also meet pure white emission in the same material.

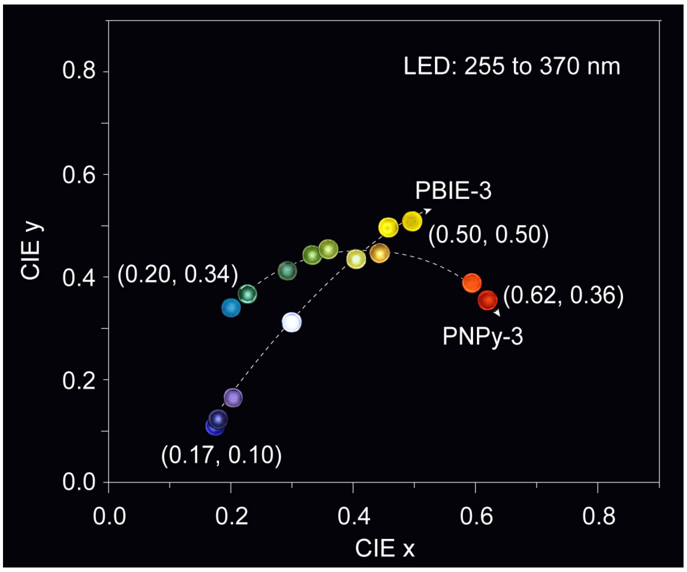

Supplementary Figure 101. Potential application in full-color afterglow display based on PBIE-3 and PNPy-3 films under direct current (DC) switched off.

Supplementary Table 19. Calculated gamut coverage relative to different standards using the multi-color afterglow of PBIE-3 and PNPy-3 films.

Afterglow	Standard	Gamut area coverage/color saturation (%) in 1931 CIEx,y
(0.17, 0.10)	ITU-R BT 2020	38.1
(0.20, 0.34)	ITU-R BT 709	72.0
(0.50, 0.50)	NTSC	51.0
(0.62, 0.36)		

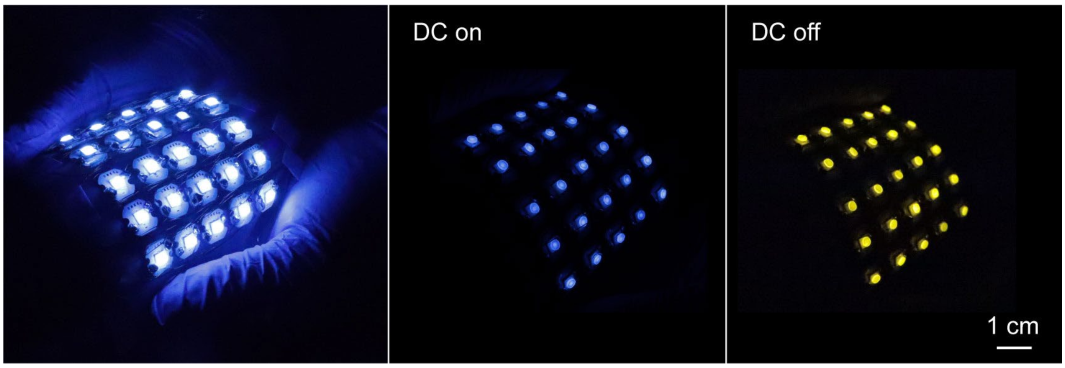

Supplementary Figure 102. Flexible display array based on PABE-d film under direct current (DC) switched on and off at bent state.

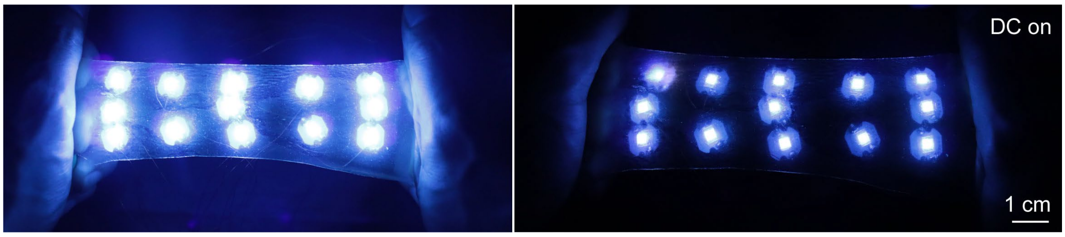

Supplementary Figure 103. Flexible display array based on PABE-d film under direct current (DC) switched on during stretching.

III. Supplementary References

1. Zheng, Y., Zhang, S., Tok, J. B. H. & Bao, Z. Molecular design of stretchable polymer semiconductors: current progress and future directions. *J. Am. Chem. Soc.* **144**, 4699–4715 (2022).
2. Chiang, Y. C. et al. Tailoring carbosilane side chains toward intrinsically stretchable semiconducting polymers. *Macromolecules* **52**, 4396–4404 (2019).
3. Xie, R. et al. Glass transition temperature from the chemical structure of conjugated polymers. *Nat. Commun.* **11**, 893 (2020).
4. Zhou, Y. et al. Cucurbit[8]uril mediated ultralong purely organic phosphorescence and excellent mechanical strength performance in double-network supramolecular hydrogels. *Dyes Pigm.* **195**, 109725 (2021).
5. Tian, R., Gao, S., Li, K. & Lu, C. Design of mechanical-robust phosphorescence materials through covalent click reaction. *Nat. Commun.* **14**, 4720 (2023).
6. Gao, Y., Deng, Z., Wang, F. & Sun, P. Achieving long lifetime of room-temperature phosphorescence via constructing vitrimer networks. *Mater. Chem. Front.* **6**, 1068–1078 (2022).
7. Wei, J. et al. Full-color persistent room temperature phosphorescent elastomers with robust optical properties. *Nat. Commun.* **14**, 4839 (2023).
8. Zhang, Y. et al. Large-area, flexible, transparent, and long-lived polymer-based phosphorescence films. *J. Am. Chem. Soc.* **143**, 13675–13685 (2021).
9. Sun, Z. et al. Shape-memorable, self-healable, recyclable, and full-color emissive ultralong organic phosphorescence vitrimers with exchangeable covalent bonds. *Adv. Optical Mater.* **10**, 2201558 (2022).
10. Zhao, Z. et al. Tough, reprocessable, and recyclable dynamic covalent polymers with ultrastable long-lived room-temperature phosphorescence. *Angew. Chem. Int. Ed.* **62**, e202301993 (2023).
11. Li, T. et al. Crosslink-enhanced strategy to achieve multicolor long-lived room temperature phosphorescent films with excellent photostability. *Chin. Chem. Lett.* **33**, 4238–4242 (2022).
12. Tao, W. et al. Strain-responsive persistent room-temperature phosphorescence from halogen-free polymers for early damage reporting through phosphorescence lifetime and image analysis. *Adv. Optical Mater.* **10**, 2102449 (2022).
13. Petersilka, M., Gossmann, U. J. & Gross, E. K. U. Excitation energies from time-dependent density-functional theory. *Phys. Rev. Lett.* **76**, 1212–1215 (1996).
14. Frisch, M. et al. Gaussian 09, Revision C.01. Gaussian, Inc., Wallingford CT (2009).
15. Liu, W., Wang, F. & Li, L. The Beijing density functional (BDF) program package: methodologies and applications. *Theor. Comput. Chem.* **2**, 257 (2003).
16. Liu, W. et al. The Beijing four-component density functional program package (BDF) and its application to EuO, EuS, YbO and YbS. *Theor. Chem. Acc.* **96**, 75 (1997).
17. Zhu, Y. L. et al. GALAMOST: GPU-accelerated large-scale molecular simulation toolkit. *J. Comput. Chem.* **34**, 2197–2211 (2013).
18. Matsen, M. W. & Schick, M. Stable and unstable phases of a diblock copolymer melt. *Phys. Rev. Lett.* **72**, 2660–2663 (1994).
19. Groot, R. D. & Madden, T. J. Dynamic simulation of diblock copolymer microphase separation. *J. Chem. Phys.* **108**, 8713–8724 (1998).
20. Ryan, A. J., Mai, S., Patrick A. Fairclough, J., Hamley, I. W. & Booth, C. Ordered melts of block copolymers of ethylene oxide and 1,2-butylene oxide. *Phys. Chem. Chem. Phys.* **3**, 2961–2971 (2001).
21. Zhao, W. et al. Rational molecular design for achieving persistent and efficient pure organic room-temperature phosphorescence. *Chem* **1**, 592–602 (2016).
22. Zhao, W., He, Z. & Tang, B. Z. Room-temperature phosphorescence from organic aggregates. *Nat. Rev. Mater*. **5**, 869–885 (2020).
23. Ma, H. et al. Room-temperature phosphorescence in metal-free organic materials. *Ann. Phys. (Berlin)* **531**, 1800482 (2019).
