# Supplementary material for: Stretchable phosphorescent polymers by multiphase engineering
Source: Nat Commun. 2024 May 15;15:4113. doi: 10.1038/s41467-024-47673-y (PMC11096371; doi:10.1038/s41467-024-47673-y)
Supplement: Supplementary file 3 — Description of Additional Supplementary Files [file 41467_2024_47673_MOESM3_ESM.pdf]

Description of Additional Supplementary Files

File Name: Supplementary Data 1
Description: Initial and final configurations of the molecular dynamics trajectories of block copolymers PABE, PABE -b, and PABE – d.

File Name: Supplementary Data 2
Description: Atomic coordinates of the optimized computational models of five initiators.

File Name: Supplementary Movie 1
Description: Ultralong phosphorescence of PABE film under 365 nm UV lamp excitation. After switching off the excitation, a bright yellow afterglow was observed by the naked eye for several seconds.

File Name: Supplementary Movie 2
Description: Demonstration of the optical and mechanical stability of PABE-d film when stretching on a universal testing machine. During the whole tensile test, PABE -d film can maintain intense ultralong phosphorescence under different stress and strain (from 0% to 600%) conditions.

File Name: Supplementary Movie 3
Description: Demonstration of multi -level volumetric data encryption. Three QR codes on a cube with blue, yellow, and red colors can be captured after switching off different UV lamps. Following a certain scanning order by smartphone, the decrypted true information IFE can be obtained.

File Name: Supplementary Movie 4
Description: Demonstration of radar detection displays mimicry. By controlling the DC off, the targets in various areas can be captured with colorful indicator lights.

File Name: Supplementary Movie 5
Description: Stretchable display based on PABE - d film. Benefitting from good optical and mechanical stability, afterglow displays can maintain function even under large mechanical deformation.
